# Down-Regulated Receptor Interacting Protein 140 Is Involved in Lipopolysaccharide-Preconditioning-Induced Inactivation of Kupffer Cells and Attenuation of Hepatic Ischemia Reperfusion Injury

**DOI:** 10.1371/journal.pone.0164217

**Published:** 2016-10-10

**Authors:** Guo Yuan, You Yu, Li Ji, Xu Jie, Li Yue, Yang Kang, Gong Jianping, Liu Zuojin

**Affiliations:** 1 Department of Infection, the Second Affiliated Hospital of Chongqing Medical University, Chongqing, 400010, China; 2 Department of Hepatobiliary Surgery, the Second Affiliated Hospital of Chongqing Medical University, Chongqing, 400010, China; University of Missouri Health Care, UNITED STATES

## Abstract

**Background:**

Lipopolysaccharide (LPS) preconditioning is known to attenuate hepatic ischemia/reperfusion injury (I/RI); however, the precise mechanism remains unclear. This study investigated the role of receptor-interacting protein 140 (RIP140) on the protective effect of LPS preconditioning in hepatic I/RI involving Kupffer cells (KCs).

**Methods:**

Sprague—Dawley rats underwent 70% hepatic ischemia for 90 minutes. LPS (100 μg/kg) was injected intraperitoneally 24 hours before ischemia. Hepatic injury was observed using serum and liver samples. The LPS/NF-κB (nuclear factor-κB) pathway and hepatic RIP140 expression in isolated KCs were investigated.

**Results:**

LPS preconditioning significantly inhibited hepatic RIP140 expression, NF-κB activation, and serum proinflammatory cytokine expression after I/RI, with an observation of remarkably reduced serum enzyme levels and histopathologic scores. Our experiments showed that protection effects could be effectively induced in KCs by LPS preconditioning, but couldn’t when RIP140 was overexpressed in KCs. Conversely, even without LPS preconditioning, protective effects were found in KCs if RIP140 expression was suppressed with siRNA.

**Conclusions:**

Down-regulated RIP140 is involved in LPS-induced inactivation of KCs and hepatic I/RI attenuation.

## Introduction

Hepatic ischemic/reperfusion injury (I/RI) is a key reason for liver dysfunction and failure after hepatic trauma, resection, and liver transplantation [1, 2]. Consequently, many methods attempting to attenuate I/RI, such as endotoxin tolerance induction, have been investigated [3–6]. Endotoxin tolerance is usually induced by single or repeated application of small amounts of lipopolysaccharide (LPS), an integral component of the cell wall of gram-negative bacilli which is closely involved in priming and deterioration in hepatic I/RI. It is known that the induction of endotoxin tolerance can render animals and humans resistant to the lethal secondary effects of LPS [7–9]. Moreover, endotoxin tolerance induction seems to be effective not only in alleviating the toxic effects of LPS but also in protecting against noninfectious challenge such as I/RI. This cross-tolerant effect is recognized as LPS preconditioning [6, 10]. It has been reported that endotoxin tolerance can attenuate I/RI in various organs including liver [11–13], while the precise molecular mechanism remains unclear. Nevertheless, in liver, it is known that endotoxin tolerance induction is closely correlated with LPS-related TLRs (Toll-like receptors)/NF-κB signal pathways of Kupffer cells (KCs) [14, 15].

KCs are resident hepatic macrophages, known to play a critical role in the pathogenesis of liver parenchyma cell damage during reperfusion phase [16]. Activated KCs are capable of releasing numerous mediators leading to the disturbance of hepatic microcirculation, which is known as an important promoter of hepatic I/RI [16, 17]. It is known that blockade of KCs’ activation can reduce hepatic I/RI in human and animal models while how to induce the blockade of KCs’ activation effectively and securely remains a problem [18, 19]. Endotoxin tolerance, induced by LPS preconditioning, has been found to inhibit the activation of macrophages which share many similarities with KCs, possibly through the suppression of NF-κB expression [13, 14]. However, due to the unclear molecular mechanism by which LPS preconditioning acts and the safety risks involved with LPS, it is not feasible to induce endotoxin tolerance through preoperative LPS preconditioning clinically. Therefore, investigating the mechanism of LPS preconditioning inducing endotoxin tolerance, and exploring the possible approach with which endotoxin tolerance can be induced without the use of LPS, can contribute to inducing blockade of KCs’ activation clinically so as to alleviate hepatic I/RI.

Recent reports suggest that receptor-interacting-protein 140 (RIP140), a co-activator for NF-κB in macrophages, may be involved with the endotoxin tolerance induction through modulating TLR(Toll-like receptor)-induced inflammatory cytokines including TNF-α, IL-1β, and IL-6 [20, 21]. Moreover, it has been found that LPS signals mediated by TLRs are severely impaired in RIP140-deficient mice [22–24]. Thus, the RIP140/LPS/TLR/NF-κB signaling cascade seems to be a potential effective regulator about the inflammatory responses against hepatic I/RI. Based on these results, we believed that RIP140 might be a novel target for endotoxin tolerance induction and hepatic I/RI therapy. Therefore, in this study, we tried to investigate the potential role of RIP140 in endotoxin tolerance induction and hepatic I/RI.

## Materials and Methods

### Animal Experiment Design

Adult male Sprague—Dawley rats (8–12 weeks old, 250–270 g in weight) were obtained from the Experimental Animal Center of Chongqing Medical University. All animals received humane care in accordance with the National Institute of Health guideline requirements in China. The animals were kept in an animal room at a constant temperature (22±1°C) and a 12-h light/dark cycle with free access to food and water. After the operation, the physical condition of the animals was monitored every half an hour. All the animals were euthanized (broken neck to death after anesthesia) in the research within 6 hours after the operation. And none of the animals became severely ill or died at anytime prior to the experimental endpoint. For anesthesia and post-surgical analgesia, all of the surgeries were performed under isoflurane anesthesia (gaseous anesthesia, 3% for anesthesia induction and 1.5% for maintenance of anesthesia and analgesia), and all efforts were made to minimize animal discomfort. Animals were randomly subjected to one of the following three groups: sham operation group, I/RI group, and LPS + I/RI group. The LPS + I/RI group (n = 20) were administered a 100 μg/kg LPS intraperitoneal injection. The sham (n = 20) and I/RI (n = 20) groups received a normal saline intraperitoneal injection of the same volume, as Sano T. et al. described elsewhere [6]. After 24 h of pretreatment, a partial warm hepatic I/RI model was established for the I/RI and LPS + I/RI groups. Briefly, following median incision, 70% partial hepatic ischemia was induced by occlusion of the vessels perfusing the left lateral and median lobes with a microvascular clip [6]. After 90 minutes of ischemia, reperfusion was accomplished by removal of the clip. The abdomen was immediately closed with a continuous suture. The sham group received a sham procedure without vascular occlusion. Then, as described in a previous work [16], animals were euthanized at 0 h and 6 h after reperfusion (n = 10 for each time point), and liver tissue samples, as well as blood from inferior vena, were collected for analysis [5].

### KC Isolation and Experimental Design

KCs were isolated from liver tissue by collagenase digestion, differential centrifugation, and selective adherence, as we described previously [25]. The viability of the isolated cells was determined by 0.1% trypan blue (Sigma, USA) exclusion and routinely exceeded 90%. To identify the KCs, immunofluorescence (ED-1 and ED-2) and phagocytic (latex beads and Dil-LDL) assays were used [25, 26]. Adenovirus expressing RIP140 (Ad-RIP140) and lentivirus with RIP140-specific siRNA (Jikai Gene Company, China) were used for transfection, manipulated according to the manufacturer’s instructions. The success of the transfection was confirmed and then the KCs were seeded in 24-well plates at 1 × 10^6^ cells per well and randomly divided into four groups as follows:

1. non-endotoxin tolerance group (media alone): the KCs were incubated in DMEM for 24 h;
2. Endotoxin tolerance group (LPS preconditioning): the KCs were incubated in DMEM containing 10 ng/ml LPS (low dose of LPS) for preconditioning for 24 h;
3. siRIP140 group (siRNA without LPS preconditioning): the KCs were transfected with siRNA-RIP140 and then incubated in DMEM for 24 h;
4. Ad-RIP140 group (Ad-RNA with LPS preconditioning): the KCs were transfected with Ad-RIP140 and then incubated in DMEM for 24 h, then 10 ng/ml LPS was added for preconditioning for another 24 h.

After that, fresh DMEM containing 100 ng/ml LPS (high dose of LPS) was added to all four groups and incubated for an additional 6 h. The additional incubation of 6 h was performed because NF-κB gave an intermediate rate of induction 6 h after LPS stimulation, as described in other studies [14]. Then the cells and supernatants were collected for further study.

### Hepatic Injury

To determine the severity of hepatic I/RI, 4 μm hematoxylin/eosin-stained liver sections were evaluated under 200 × magnification. A semi-quantitative score (0–4) was used for the assessment of necrosis, hepatocellular vacuolization, sinusoidal congestion, and ballooning degeneration, as described by Suzuki [27]. The serum level of ALT was also quantified with an automatic biochemical analyzer (Beckman CX7, USA) as an indicator of hepatocellular injury.

### Immunohistochemical Analysis

The livers were fixed in 10% buffered formalin and dehydrated through increasing concentrations of ethanol in xylene and embedded in paraffin, and were then cut into blocks of 3 mm × 3 mm × 3 mm. Immunohistochemical staining was performed using rabbit anti-mouse RIP140 polyclonal antibody, sc-8997 (Santa Cruz, USA), as the primary antibodies. Cells showing brown grain were defined as positive.

### Immunofiuorescence Assay

To identify the location and expression level of RIP140 in KCs under different circumstances, an immunofiuorescence assay was performed. After exposure to the 100 ng/ml LPS stimuli for 6 h, RIP140 observation was performed with a Leica TCS SP2 laser scanning confocal microscope, using excitation spectral laser lines at 405 nm and 594 nm, as described elsewhere [16]. DAPI was used to show the position of KC nuclei, which appeared as blue dye cell nuclei. RIP140 expression showed a distinct nuclear pattern in green.

### Western Blot Analysis

RIP140 protein levels in livers and KCs were measured by western blot analysis. In brief, cell and liver samples were lysed with RIPA buffer containing a combination of protease inhibitors. The protein concentrations of the lysates were measured using a BCA assay kit. Equal amounts of protein from different samples were then electrophoretically separated using 12% SDS-PAGE and transferred to 0.45-mm PVDF membranes. After being blocked with 5% non-fat dried milk for 1 h, membranes were incubated with RIP140 antibodies (sc-8997; Santa Cruz, USA) overnight at 4°C. After washing, secondary antibody was applied for 1 h at 37°C. The relative amount of RIP140 protein was quantified from relative absorption of the band of the protein of interest to β-actin, using Bandscan (v. 5.0).

### Real Time Polymerase Chain Reaction

Total RNA was extracted from cultured cells and liver samples using RNAiso Plus reagent (TaKaRa, Japan) according to the manufacturer’s instructions, and cDNA was synthesized using PrimeScript RT reagent kit (TaKaRa, Japan). Real-time PCR analyses were performed using a CFX96TM real-time system with SYBR Green II Master Mix (TaKaRa, Japan). The gene-specific primer sets were synthesized by Invitrogen and included the following: RIP140, 5′-CCA TCA ATC TTT CCC AGC AC-3′, and 5′-GGA CTC TTT GCC TTT CGT GA- 3′; TNF- α, 5′-GAC CCT CAC ACT CAG ATC ATC-3′, and 5′-GAA CCT GGG AGT AGA TAA GG-3′; IL-1β, 5′-CAG AAG AAT CTA GTT GTC CGT G-3′, and 5′-CAG AAG AAT CTA GTT GTC CGT G-3′; IL-6, 5′-GTA TGA ACA ACG ATG ATG CAC TTG-3′, and 5′-ATG GTA CTC CAG AAG ACC AGA GGA-3′; β-actin, 5′-CAG TGC CAG CCT CGT CTC AT -3′, and 5′-AGG GGC CAT CCA CAG TCT TC-3′. The relative quantities of RIP140 and TNF-α mRNA were normalized to the level of the house-keeping gene β-actin.

### Enzyme-Linked Immunosorbent Assay

The TNF-α levels in serum and supernatants were measured using commercial enzyme-linked immunosorbent assay (ELISA) kits, according to manufacturer’s instructions (Santa Cruz, USA). NF-κB activity in nuclear extracts was measured using the Trans-AM NF-κB p65 ELISA kit (Active Motif Europe, Belgium), as described in our previous study [16]. Briefly, 5 μg of nuclear extract was added to a 96-well plate on which oligonucleotide containing NF-κB-consensus binding site had been immobilized. The NF-κB complex was detected by adding a specific p65 subunit mAb. A secondary horseradish-peroxydase conjugated mAb was added and developed with tetramethylbenzidine substrate. After an optimal development time, the reaction was stopped using H_2_SO_4_ 0.5 mol/l, and absorbance was measured at 450 nm.

### Statistical Analysis

Results were expressed as mean ± standard deviation (SD). Comparative analyses were conducted using one-way ANOVA tests and independent *t* tests, followed by Tukey post hoc testing. All analyses were carried out with the SPSS 19.0 software (SPSS Inc, USA). P < 0.01 was considered significant.

## Results

### LPS Preconditioning Significantly Alleviated Hepatic I/RI

**Fig 1:**
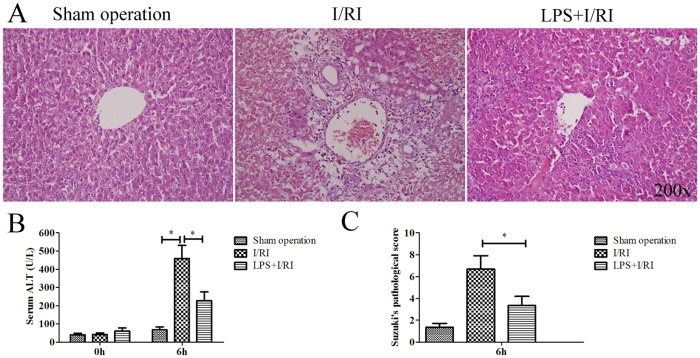
The influence of LPS preconditioning dose on hepatic ischemia/reperfusion injury. Animals subjected to 90 minutes of 70% hepatic ischemia, followed by 6 h reperfusion, then the hepatic I/RI was evaluated. **(A)** The H&E staining of the liver from sham group, LPS + I/RI group and I/RI group. **(B)** ALT serum level. **(C)** Suzuki’s pathological score (*, *P* < 0.01).

Hepatic I/RI was determined using Suzuki’s pathological score and the serum levels of ALT, 6 h after reperfusion. We found that disorder of the hepatic lobular architecture of the I/RI group was more severe than that of the sham group, reflected by the presence of more necrotic cells, ballooning degeneration, and infiammatory cell infiltration in the I/RI group. Simultaneously, as with pathological changes of the liver (Fig 1A and 1B), serum enzyme data from the I/RI group indicated significantly higher ALT levels (*P* < 0.01) than the sham group. Moreover, compared with I/RI group, LPS+I/RI group (LPS preconditioning group) showed significantly attenuated I/RI, with lower ALT levels and less hepatic lobule architecture changes (*P* < 0.01). The Suzuki scores in the sham, I/RI, and LPS + I/RI groups were: 1.35 ± 0.36, 6.68 ± 1.23, and 3.35 ± 0.84, respectively (Fig 1C) (*P* < 0.01). These data suggested that administration of LPS preconditioning could significantly attenuate liver I/RI.

### LPS Preconditioning Depressed RIP140 Expression and NF-κB Activity

**Fig 2:**
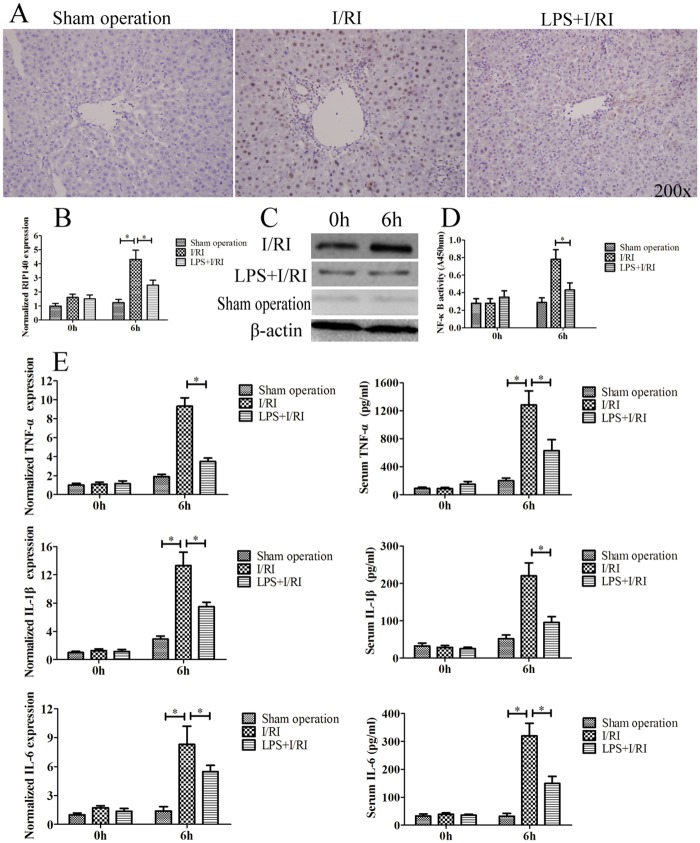
The influence of LPS preconditioning on RIP140 expression and NF-κB signal transduction. Hepatic RIP140 expression in the rats from different groups was examined with immunohistochemical analysis **(A)**, RT-PCR **(B)**, and western blot **(C)**. Hepatic NF-κB activity **(D)**, and TNF-α, IL-1β, and IL-6 expression levels **(E)** were examined with ELISA and RT-PCR (*, *P* < 0.01).

Many studies have reported that the mechanism of LPS preconditioning-induced endotoxin tolerance is closely correlated with the suppression of NF-κB activation, and that RIP140 may act as a co-activator for NF-κB to modulate the TLR4 signaling pathway to produce inflammatory cytokines such as TNF-α, IL-1β, and IL-6 (20–24). Therefore, we examined RIP140 expression in the liver with immunohistochemical analysis, RT-PCR, and western blot (Fig 2A and 2B). Hepatic NF-κB activity and involved cytokine expression (TNF-α, IL-1β, and IL-6) were also examined with PCR and ELISA (Fig 2C and 2D). The results showed that, compared with I/RI group, LPS preconditioning in LPS+I/RI group could significantly reduce the expression of RIP140 and NF-κB activity and that cytokine expression was involved (p < 0.01). These data suggested that pre-ischemic administration of LPS could down-regulate RIP140 expression, interfere with NF-κB activity to modulate inflammatory responses, and initiate protection against hepatic I/RI.

### The Influence of RIP140 on Endotoxin Tolerance Induction in KCs

**Fig 3:**
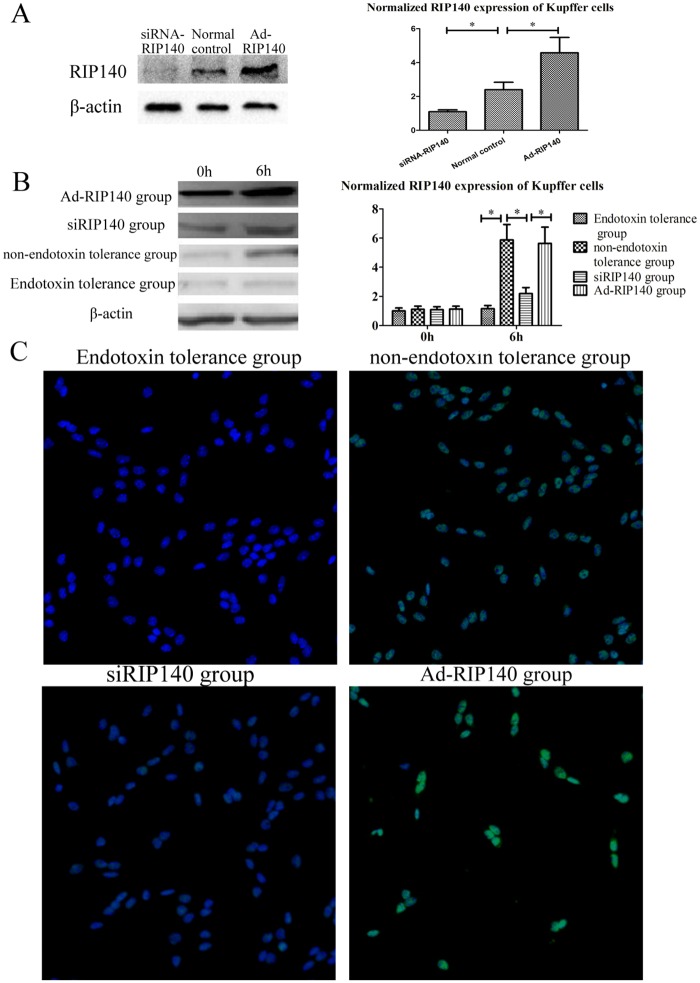
The influence of LPS on RIP140 expression of KCs. The RIP140 knockdown and overexpression in KCs were assessed using RT-PCR and WB **(A)**. KC RIP140 expression was examined with RT-PCR, western blot **(B)**, and immunofluorescence assay **(C)** (*, *P* < 0.01).

**Fig 4:**
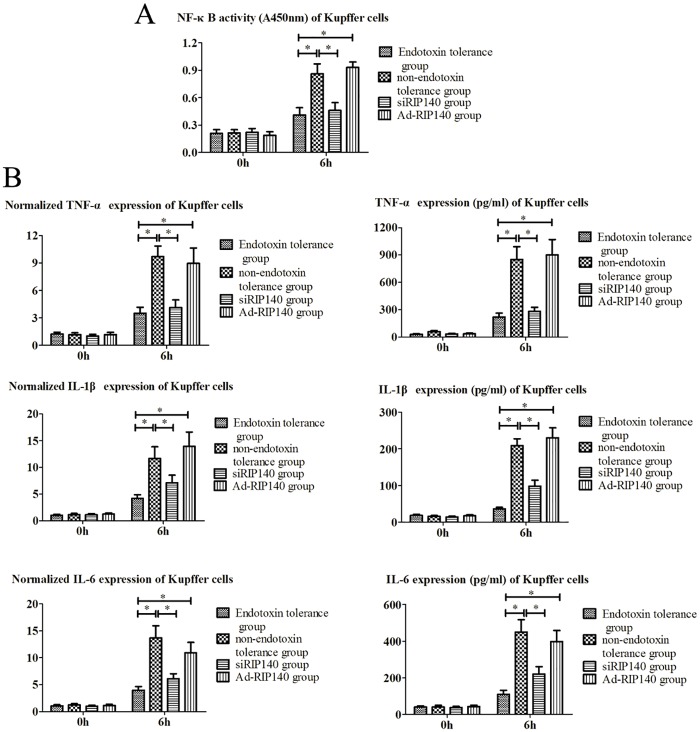
The influence of RIP140 expression on NF-κB signal transduction in KCs after a high dose of LPS. After the high dose of LPS (100 ng/ml) stimulation, the NF-κB activity of KCs **(A)** and the supernatant inflammatory cytokine (including TNF-α, IL-1β, and IL-6) expression **(B)** of different groups were examined with ELISA and RT-PCR (*, *P* < 0.01).

Activated KCs are known to play a major role in the pathogenesis of liver parenchyma cell damage during the reperfusion phase, and inducing the endotoxin tolerance of KCs has been shown to be an effective approach to attenuation of hepatic I/RI [16]. For this reason, the role of RIP140 in endotoxin tolerance induction of KCs was investigated with the purpose of exploring the role of RIP140 in hepatic I/RI. The knockdown and overexpression of RIP140 in KCs were determined using PCR and WB (Fig 3A). The RIP140 expression of KCs of different groups was examined with RT-PCR, Western blot analysis, and immunofiuorescence assay (Fig 3B and 3C). NF-κB activity of KCs and the supernatant levels of TNF-α, IL-1β, IL-6 were measured using PCR and ELISA (Fig 4). In the Endotoxin tolerance group, the RIP140 expression and NF-κB activity of KCs and the supernatant inflammatory cytokine expression (including TNF-α, IL-1β, and IL-6) were all much lower than those in the non-endotoxin tolerance group (Figs 3 and 4). These results indicated that preconditioning with a low dose of LPS (10 ng/ml) could induce endotoxin tolerance effectively in KCs, which markedly attenuated KCs’ response to the subsequent high dose of LPS (100 ng/ml) stimulation. However, with higher RIP140 expression, although the KCs in the Ad-RIP140 group were preconditioned with a low dose of LPS, similar to the Endotoxin tolerance group, the NF-κB activity and inflammatory cytokine expression were still much higher than in the Endotoxin tolerance group. Conversely, the siRIP140 group did not receive low-dose LPS pretreatment, but the RIP140 expression, NF-κB activity, and the inflammatory cytokine expression in this group were significantly lower than in the non- endotoxin tolerance group after high-dose LPS stimulation. These results indicated that endotoxin tolerance in KCs, induced with a low dose of LPS, was closely correlated with RIP140. Like the inhibition of NF-kB activity, the down-regulation of RIP140 was found to prevent the inflammatory response of KCs driven by a high-dose of LPS effectively.

## Discussion

It is known that LPS preconditioning-induced endotoxin tolerance can attenuate hepatic I/RI [28, 29]. Although the details of the underlying mechanisms remain unclear, blocking the LPS/TLR4/NF-κB signal pathway is believed to be a key step [28–31]. Concerning this signal pathway, the discovery of RIP140 was reported recently [20, 21]. RIP140 is a co-activator for NF-κB, interacting with the nuclear factor κ light-chain enhancer of NF-κB subunit RelA, and RIP140 degradation, triggered by the exposure of TLR (Toll-like receptor) ligands, has been shown to negatively regulate specific genomic targets in inflammatory response to promote endotoxin tolerance [22]. The SOCS (suppressor of cytokine signaling)-associated SCF (Skp1-Cullins- F-box) E3 ligase complex not only promotes ubiquitination and proteasome mediated degradation of RIP140 by binding to RelA, but also contributes to the alteration of NF-κB transcriptional activity on the promoters of pro-inflammatory cytokines including TNF-α, IL-1β, and IL-6. Through this complex, LPS was found to possess the capacity of down-regulating the expression of RIP140 [23, 24]. However, the correlation between expression of RIP140 and LPS preconditioning-induced endotoxin tolerance in hepatic I/RI remain unclear.

In this study, we firstly tested the role of RIP140 in hepatic I/RI using an animal experiment. Being consistent with other reports [6], our results showed that preconditioning with LPS (100 μg/kg, intraperitoneal injection) could effectively ameliorate rat liver ischemic/reperfusion injury: the pathological score and serum concentrations of ALT were significantly lower in the LPS+I/RI group than in the I/RI group after reperfusion. Besides, the results indicated that LPS preconditioning could significantly down-regulate RIP140 expression in the liver after reperfusion. Moreover, the downstream signaling cascades were also down-regulated, as indicated by the inactivation of NF-κB and lower level of pro-inflammatory cytokines. Taken together, these results suggested a potential correlation between down-regulation of RIP140 and LPS preconditioning-induced endotoxin tolerance in hepatic I/RI.

Macrophages are reported to play a crucial role in LPS preconditioning-induced endotoxin tolerance. Inducing the inactivation of KCs which are located in the liver and share many of the functions of macrophages, has been demonstrated to be the essential mechanism underlying the hepatic I/RI attenuation induced by endotoxin tolerance [16–19]. Thus, in order to confirm the role of RIP140 in hepatic I/RI, the correlation between RIP140 and inactivation of KCs was also explored. These observations showed that a low dose of (10 ng/ml) LPS preconditioning could induce endotoxin tolerance and hypo-response of KCs to the second high dose of LPS (100 ng/ml) challenge effectively. This was accompanied by down-regulated RIP140 expression. However, for the KCs with over-expressed RIP140, endotoxin tolerance could not be induced after low-dose LPS preconditioning. Conversely, after transfection with RIP140-specific siRNA, KCs could hardly activated by the second, high dose LPS, even without low-dose LPS preconditioning. Taken together, these results indicated the critical role of RIP140 between LPS-preconditioning-induced endotoxin tolerance and the inactivation of KCs.

KCs play a key role in the pathological process of hepatic I/RI and they are responsible for the production of various cytokines that affect LPS/TLRs/NF-κB signaling pathways, such as TNF-α, IL-1β, and IL-6 [32, 33]. Those cytokines possess pleiotropic activities (such as induction of adhesion molecules, activation of complements, and promotion of apoptotic or necrotic cell death) and their overexpression has been found to be the direct promoter of hepatic I/RI [34, 35]. Many reports have demonstrated the close correlation between KCs’ inactivation-induced inhibition of those cytokines and LPS-preconditioning-induced hepatic I/RI attenuation [36, 37], suggesting the key role of KCs’ inactivation in hepatic I/RI. In this study, our results showed that the down-regulation of RIP140 could lead to the inactivation of KCs. Thus, considering the key role of KC inactivation in hepatic I/RI, our results suggested the potential candidate role of RIP140 in hepatic I/RI therapy.

In conclusion, we demonstrated that (a) LPS preconditioning-induced endotoxin tolerance in hepatic I/RI is accompanied by down-regulated RIP140, and (b) RIP140 down-regulation plays a crucial role in LPS-preconditioning-induced inactivation of KCs. Safety considerations make it almost impossible to use LPS preconditioning in clinical treatment. Thus, based on our results and the key role of KCs’ inactivation in hepatic I/RI, RIP140 might be a suitable therapeutic target for hepatic I/RI.

## Supporting Information

S1 File The figures and statistical data for Fig 1. (ZIP)

S2 File The figures and statistical data for Fig 2. (ZIP)

S3 File The figures and statistical data for Fig 3. (ZIP)

S4 File The statistical data for Fig 4. (ZIP)

S5 File The supporting figures for Fig 3. (ZIP)
