# Supplementary figures and images for: Down-Regulated Receptor Interacting Protein 140 Is Involved in Lipopolysaccharide-Preconditioning-Induced Inactivation of Kupffer Cells and Attenuation of Hepatic Ischemia Reperfusion Injury
Source: PLoS One. 2016 Oct 10;11(10):e0164217. doi: 10.1371/journal.pone.0164217 (PMC5056758; doi:10.1371/journal.pone.0164217)

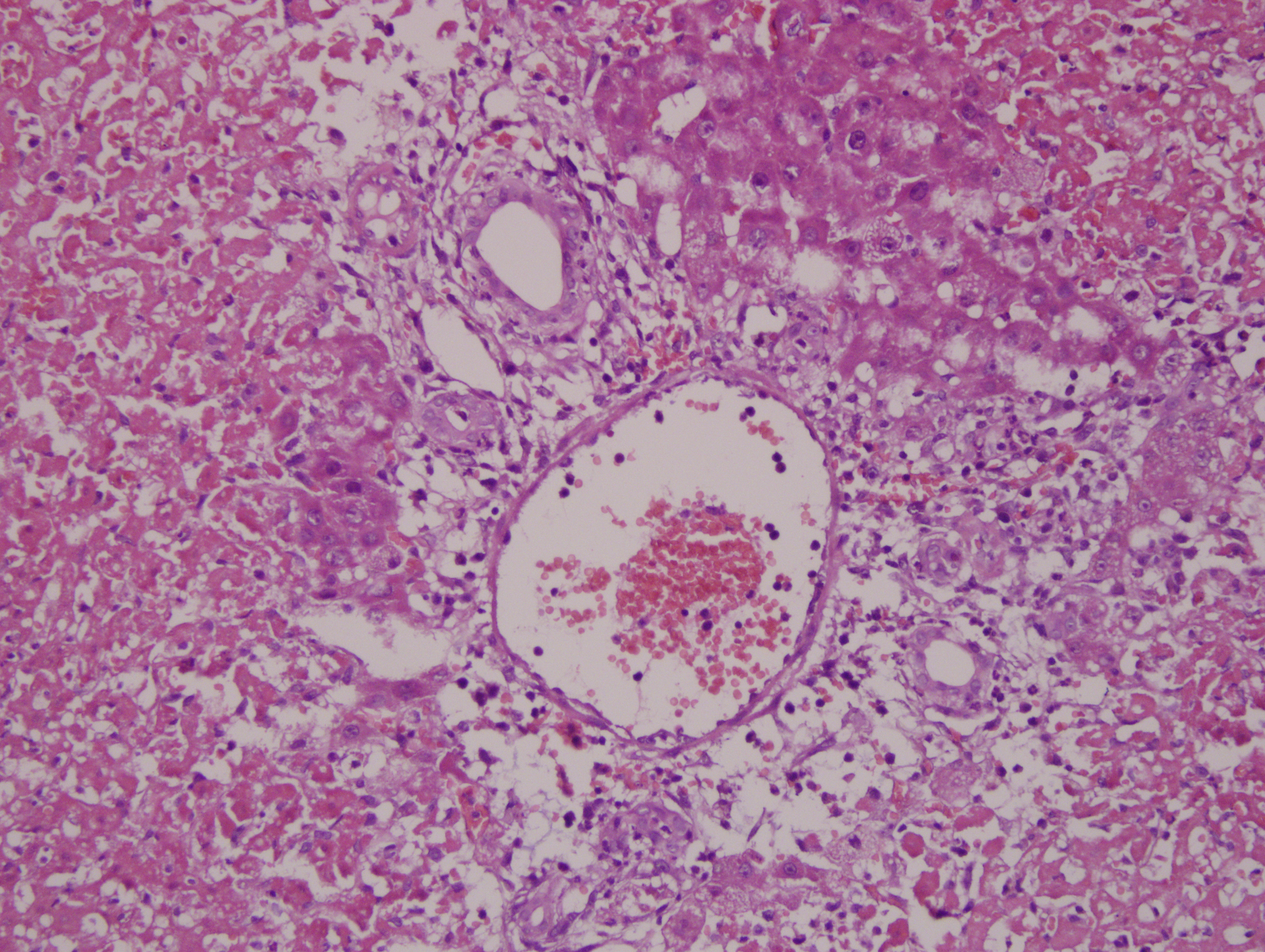

Supplement: S1 File — (ZIP) [file pone.0164217.s001.zip › S1_Fig 1/IRI.jpg]

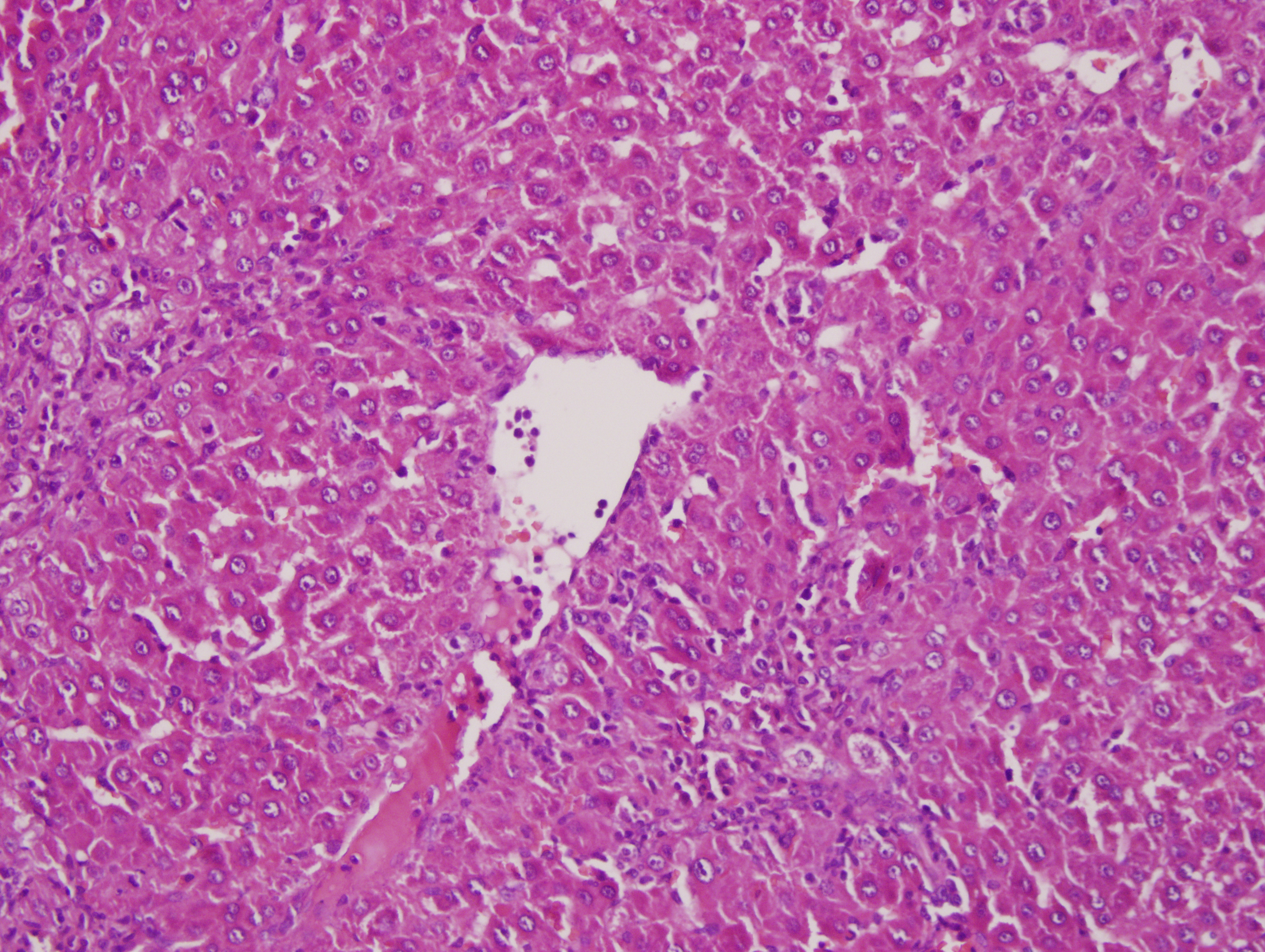

Supplement: S1 File — (ZIP) [file pone.0164217.s001.zip › S1_Fig 1/LPS+IRI.jpg]

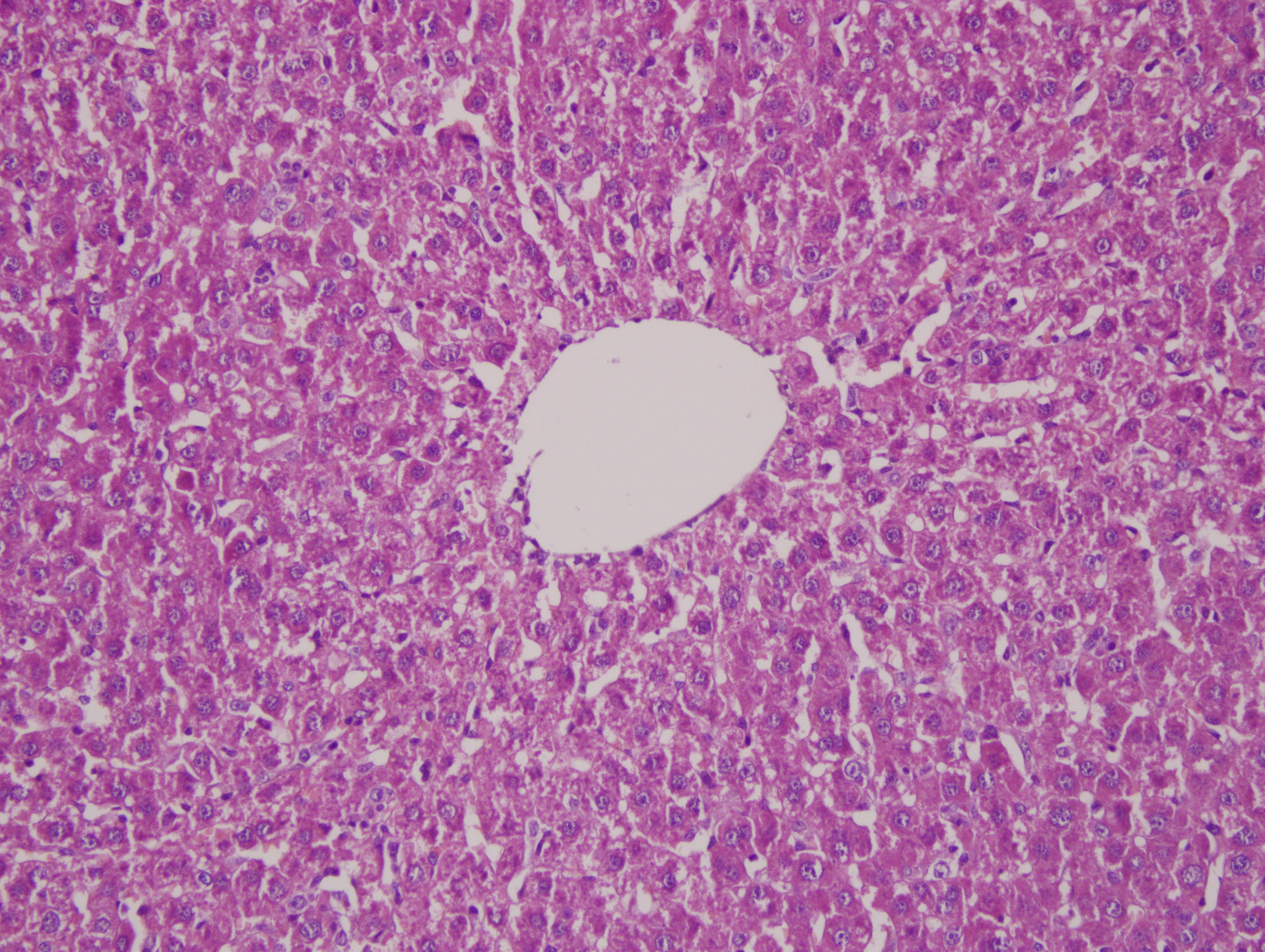

Supplement: S1 File — (ZIP) [file pone.0164217.s001.zip › S1_Fig 1/Sham operation.jpg]

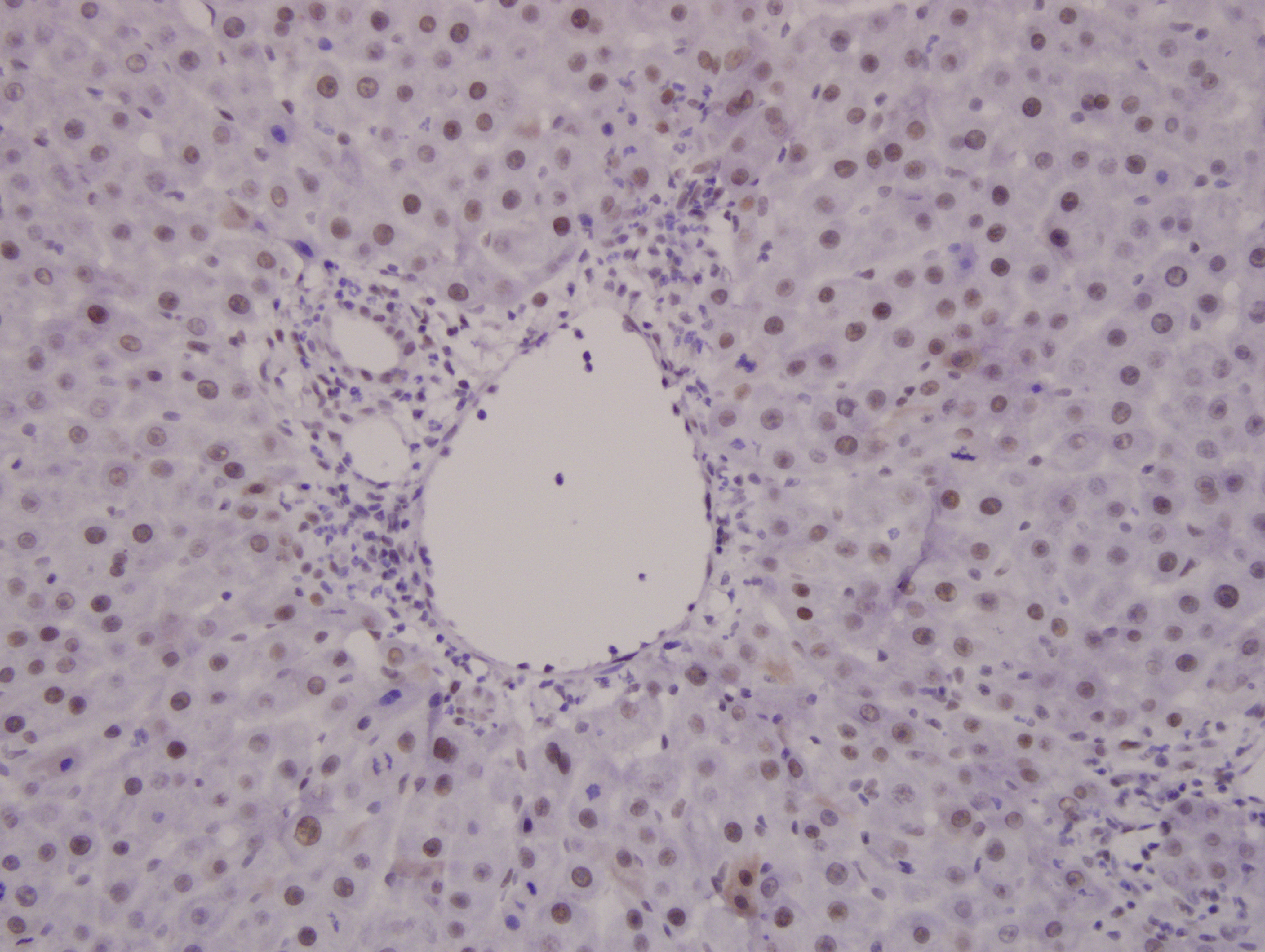

Supplement: S2 File — (ZIP) [file pone.0164217.s002.zip › S2_Fig 2/IRI.jpg]

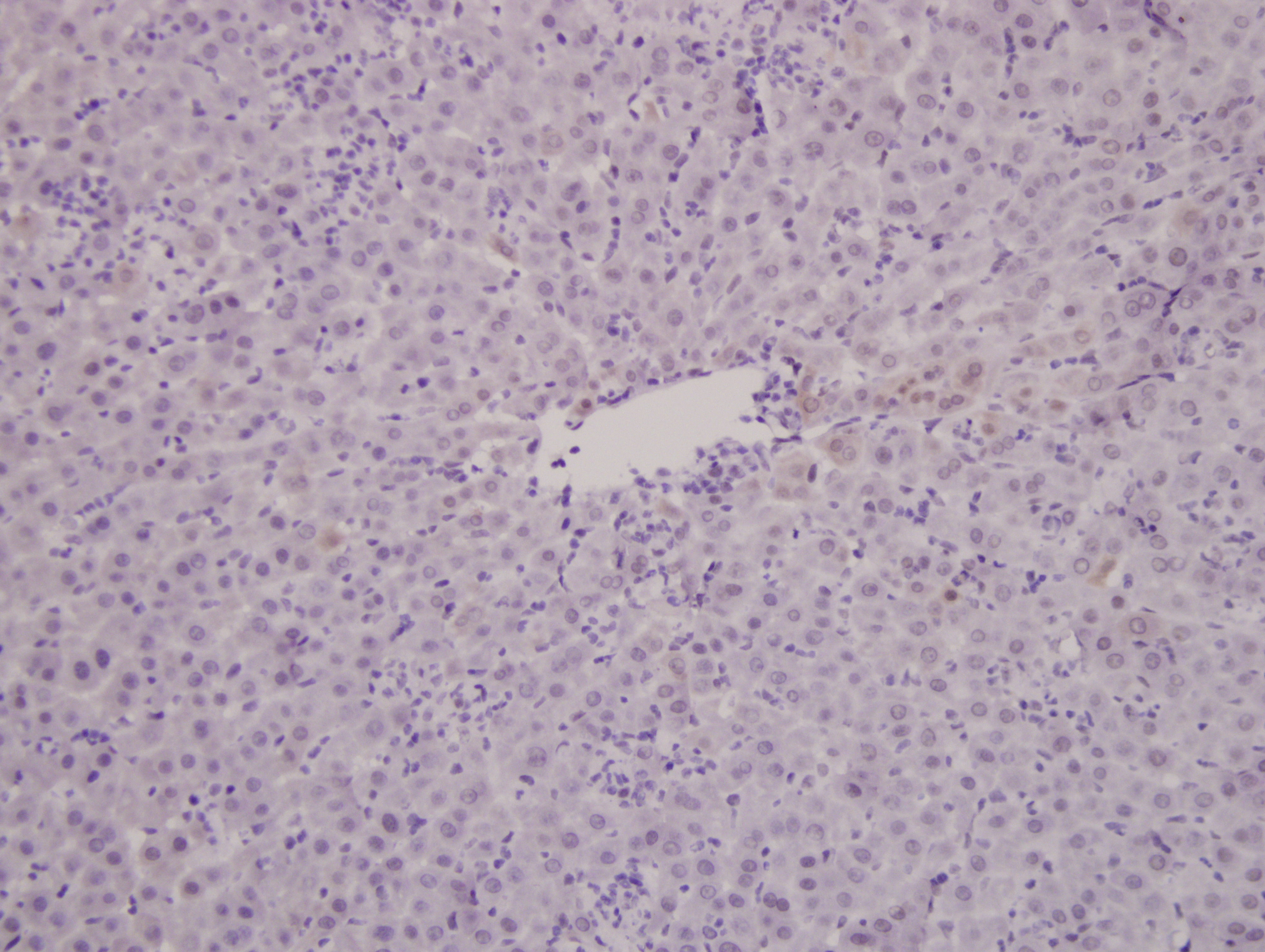

Supplement: S2 File — (ZIP) [file pone.0164217.s002.zip › S2_Fig 2/LPS+IRI.jpg]

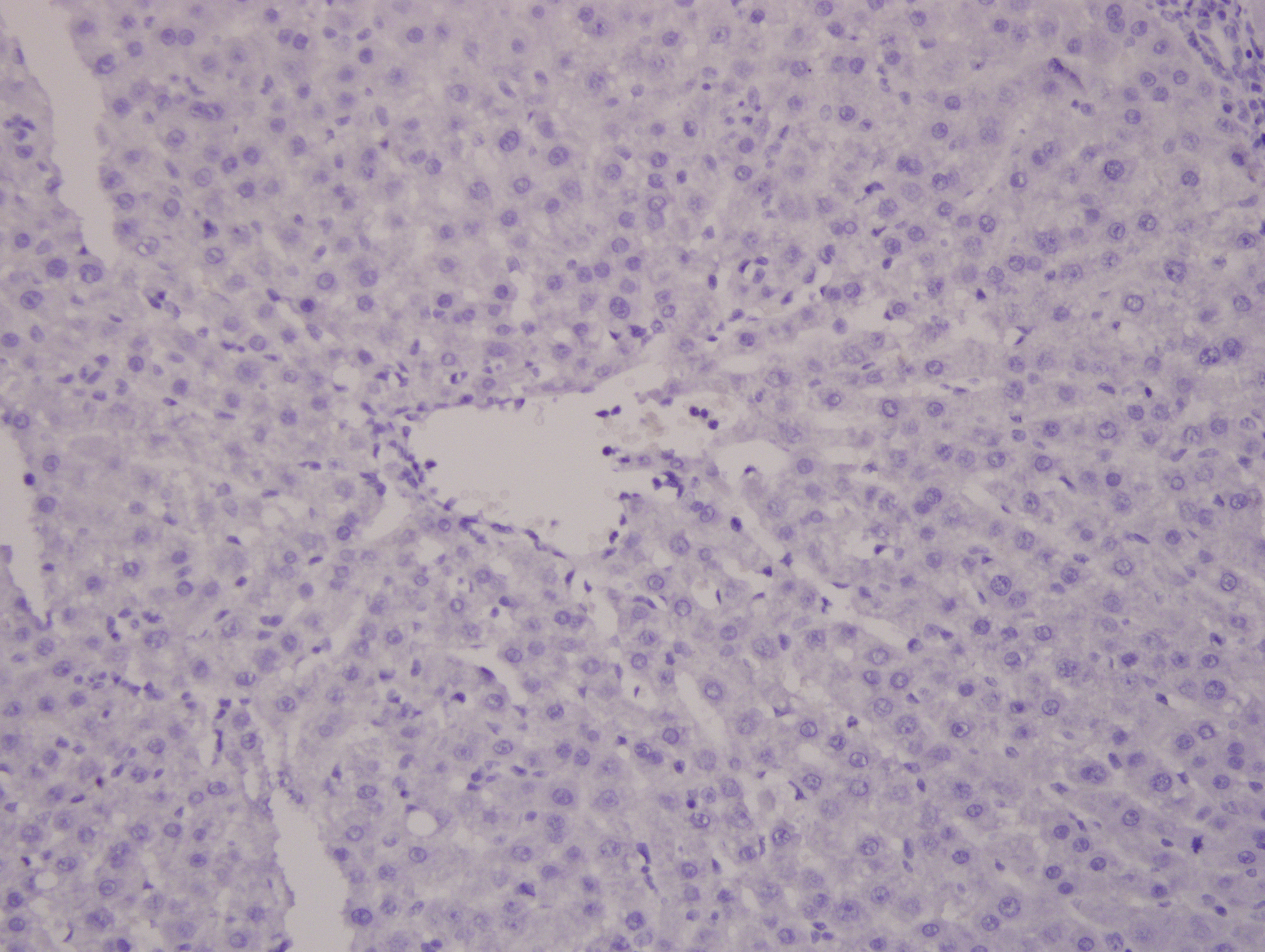

Supplement: S2 File — (ZIP) [file pone.0164217.s002.zip › S2_Fig 2/Sham operation.jpg]

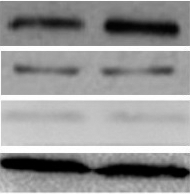

Supplement: S2 File — (ZIP) [file pone.0164217.s002.zip › S2_Fig 2/WB.tif]

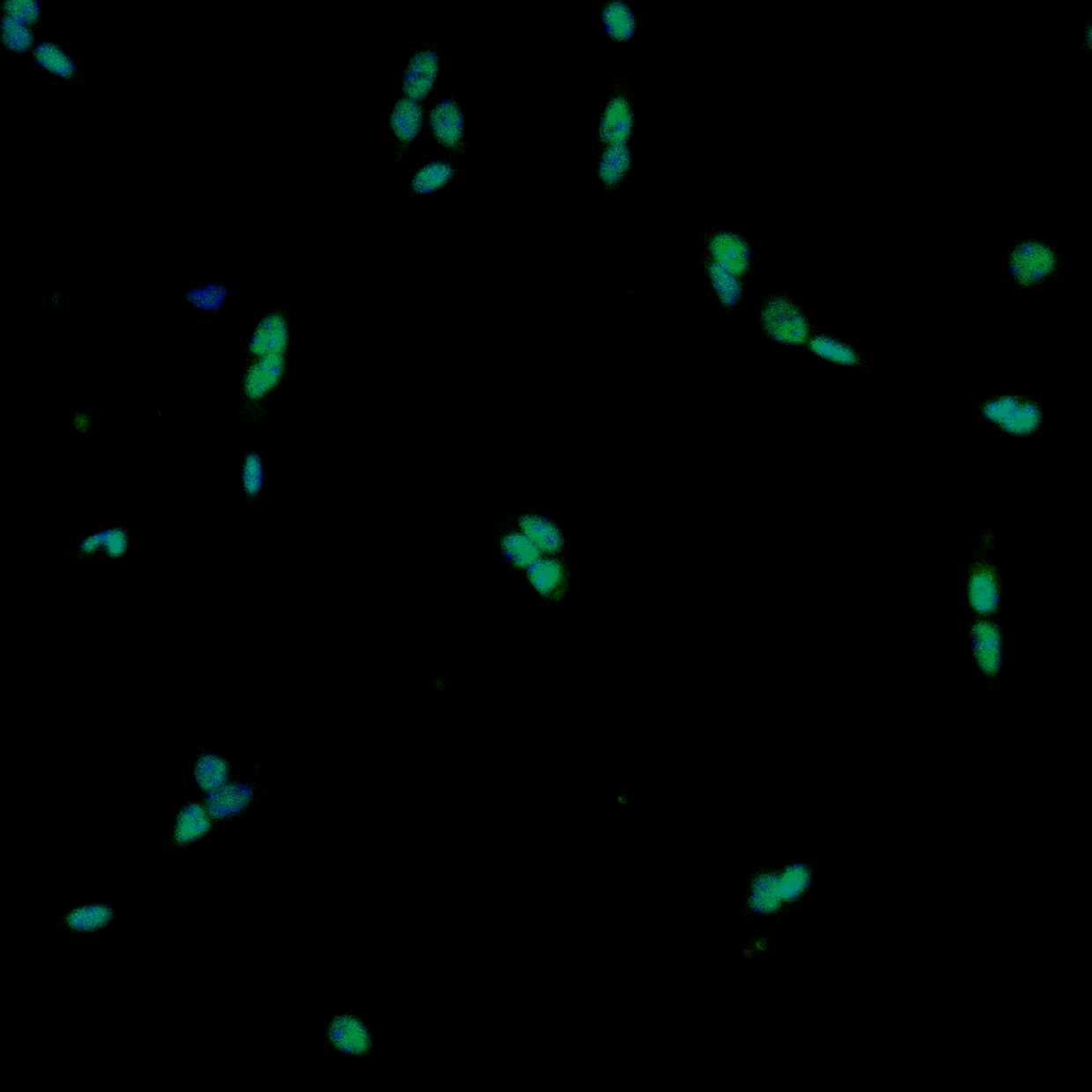

Supplement: S3 File — (ZIP) [file pone.0164217.s003.zip › S3_Fig 3/Ad-RIP140 group.tif]

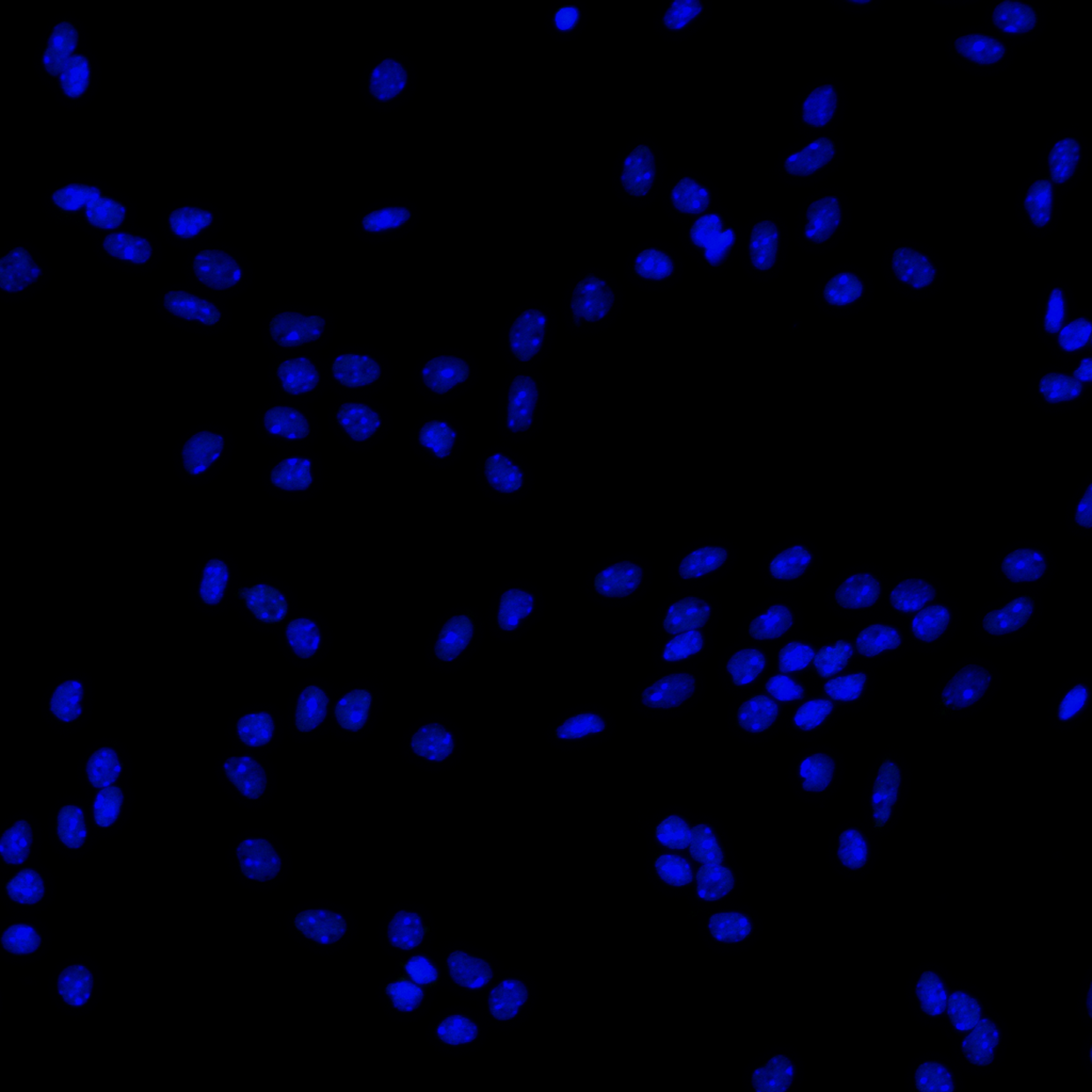

Supplement: S3 File — (ZIP) [file pone.0164217.s003.zip › S3_Fig 3/ET group.tif]

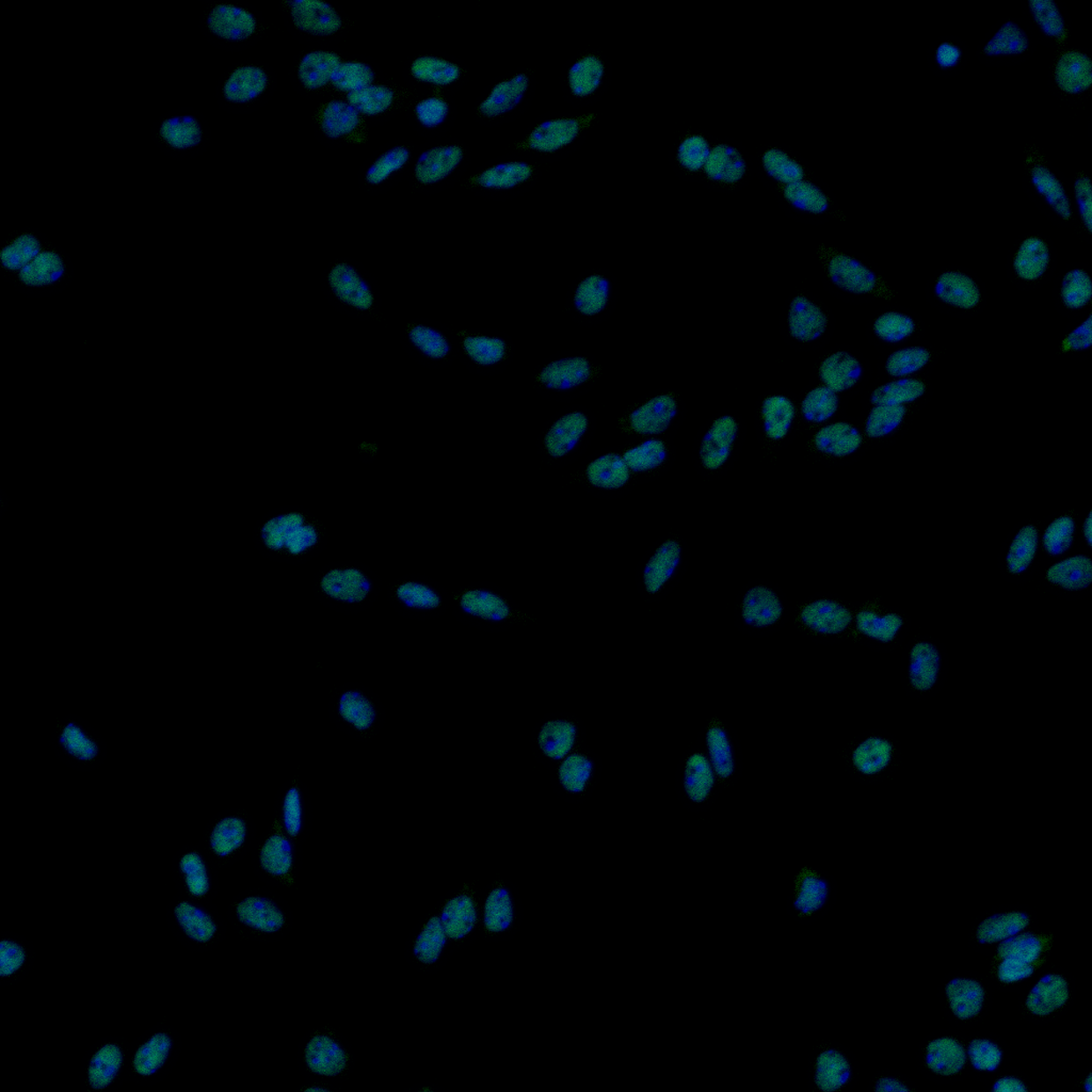

Supplement: S3 File — (ZIP) [file pone.0164217.s003.zip › S3_Fig 3/NET group.tif]

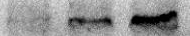

Supplement: S3 File — (ZIP) [file pone.0164217.s003.zip › S3_Fig 3/RIP140 knockdown and overexpression.tif]

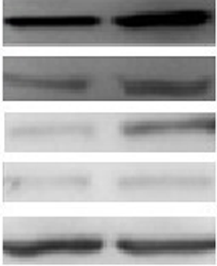

Supplement: S3 File — (ZIP) [file pone.0164217.s003.zip › S3_Fig 3/WB.tif]

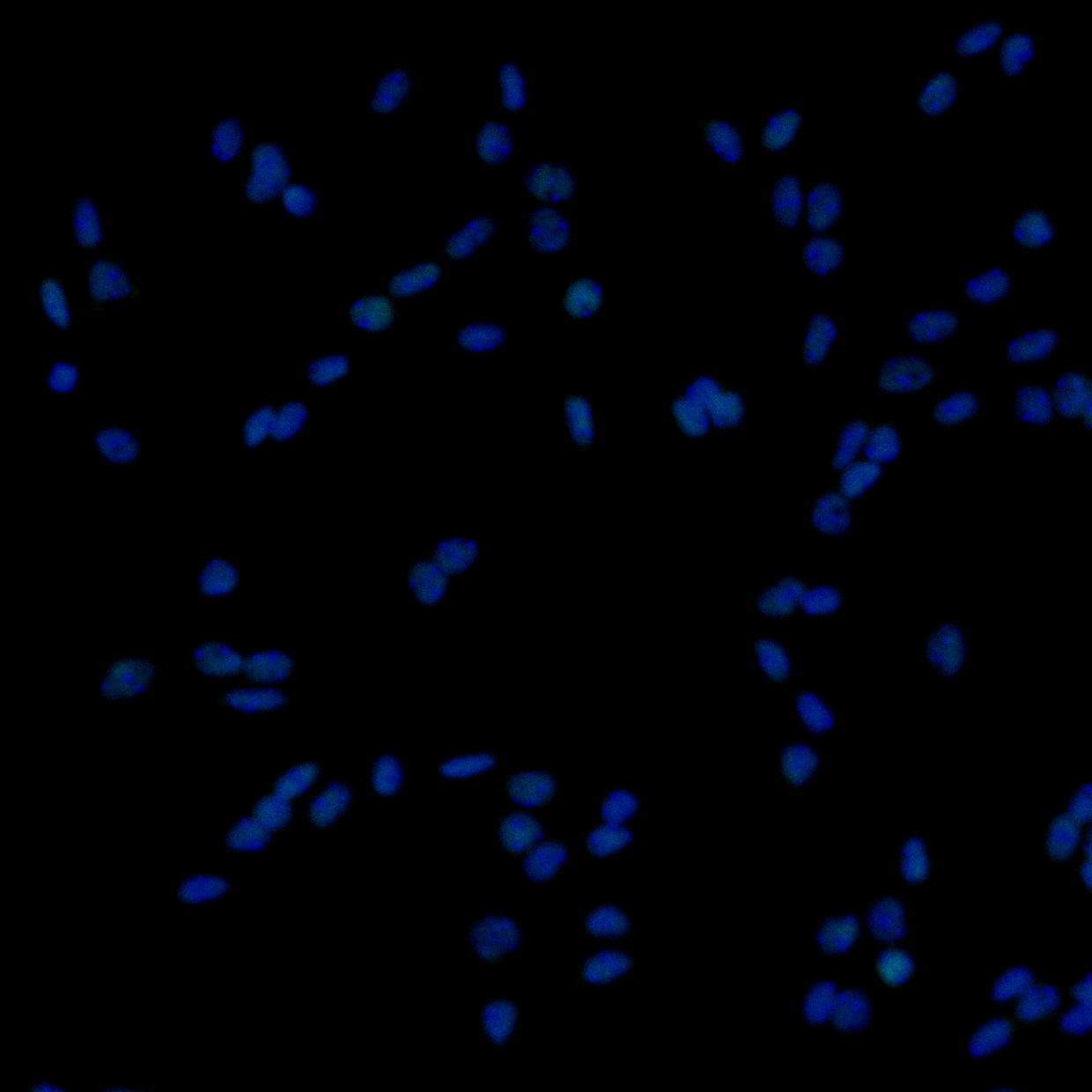

Supplement: S3 File — (ZIP) [file pone.0164217.s003.zip › S3_Fig 3/siRNA-RIP140 group.tif]

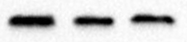

Supplement: S3 File — (ZIP) [file pone.0164217.s003.zip › S3_Fig 3/a┬-actin.tif]

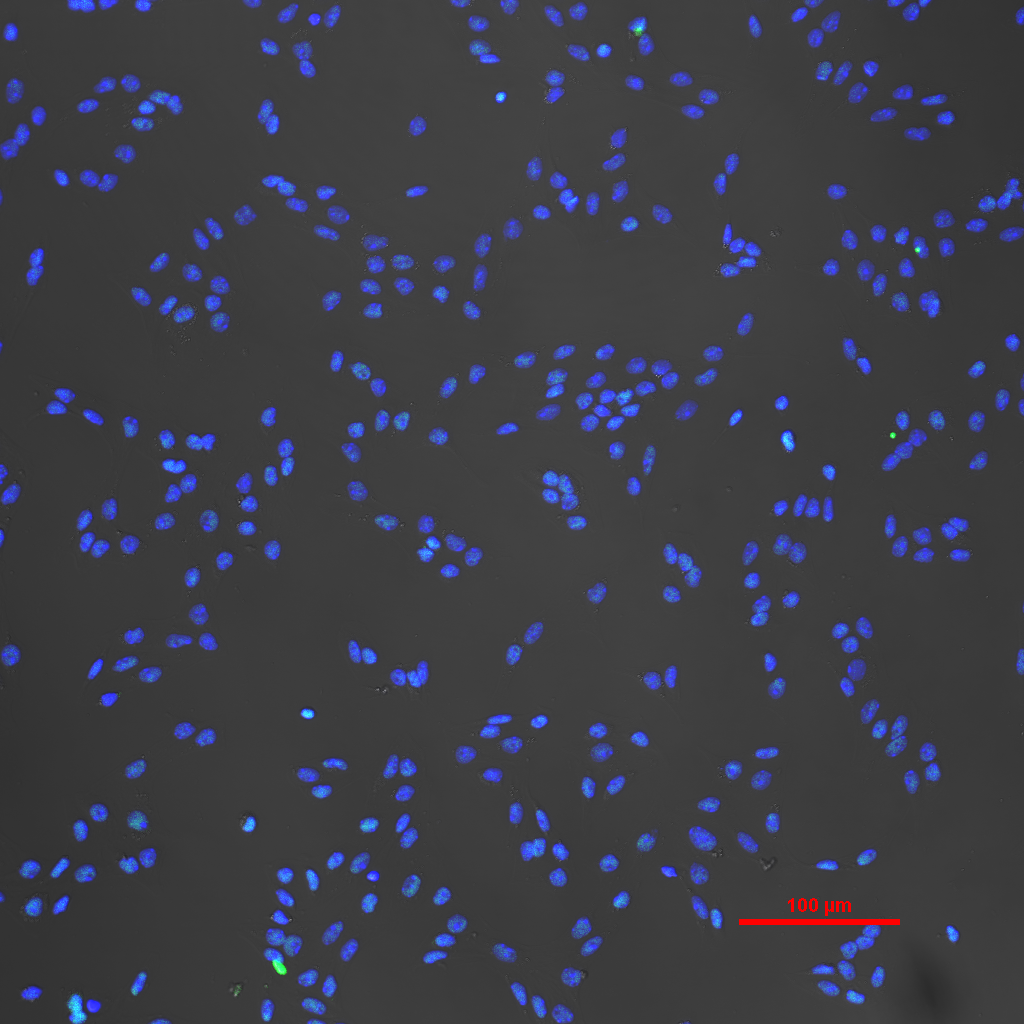

Supplement: S5 File — (ZIP) [file pone.0164217.s005.zip › Some other supplemental files (SOSF)/ICC.TIF/ET/et-200.tif]

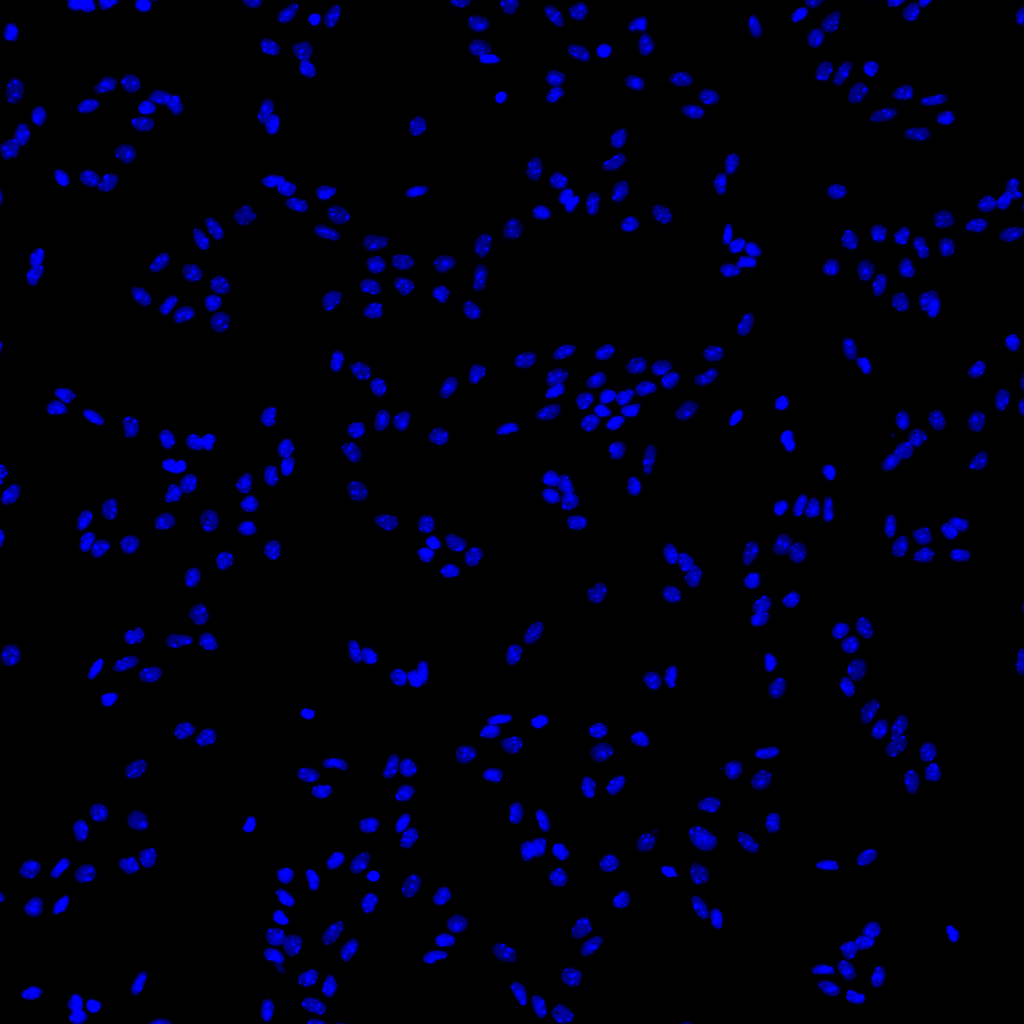

Supplement: S5 File — (ZIP) [file pone.0164217.s005.zip › Some other supplemental files (SOSF)/ICC.TIF/ET/et-200c1.tif]

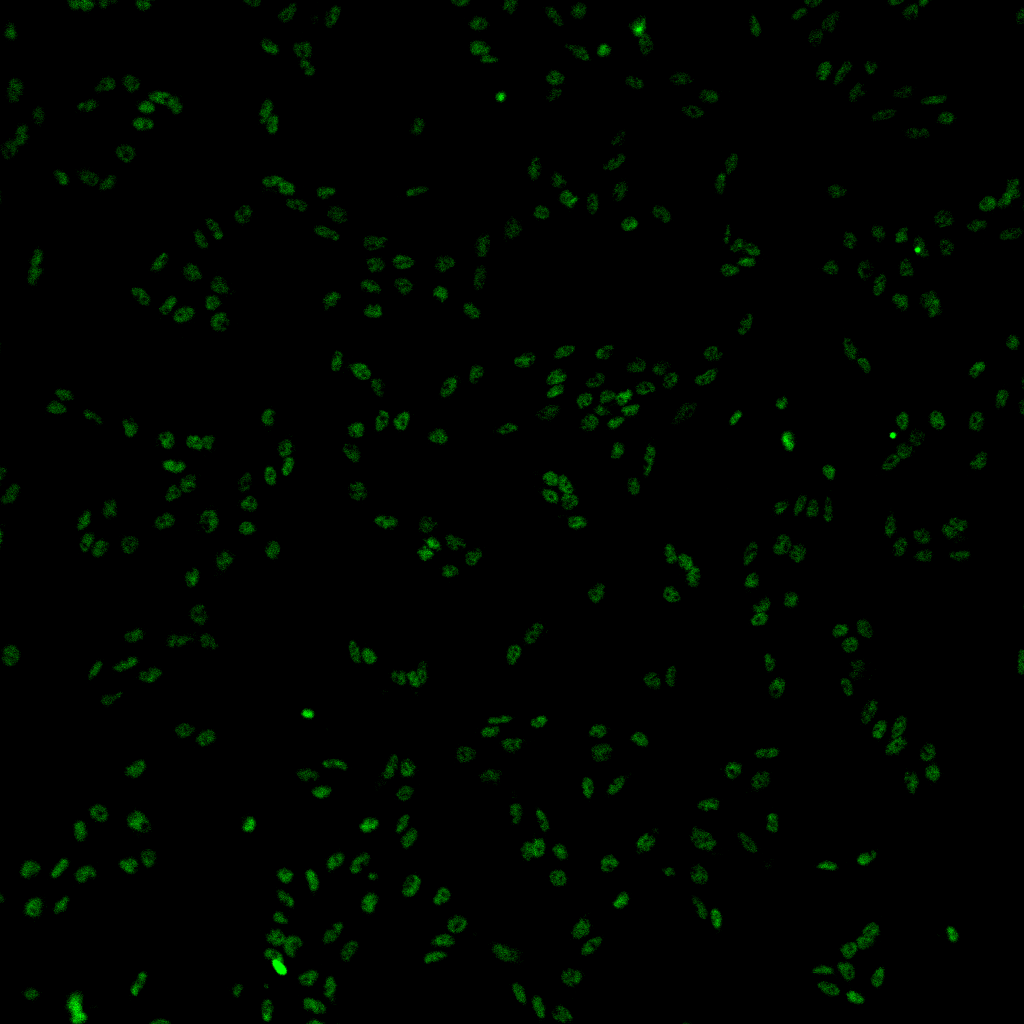

Supplement: S5 File — (ZIP) [file pone.0164217.s005.zip › Some other supplemental files (SOSF)/ICC.TIF/ET/et-200c2.tif]

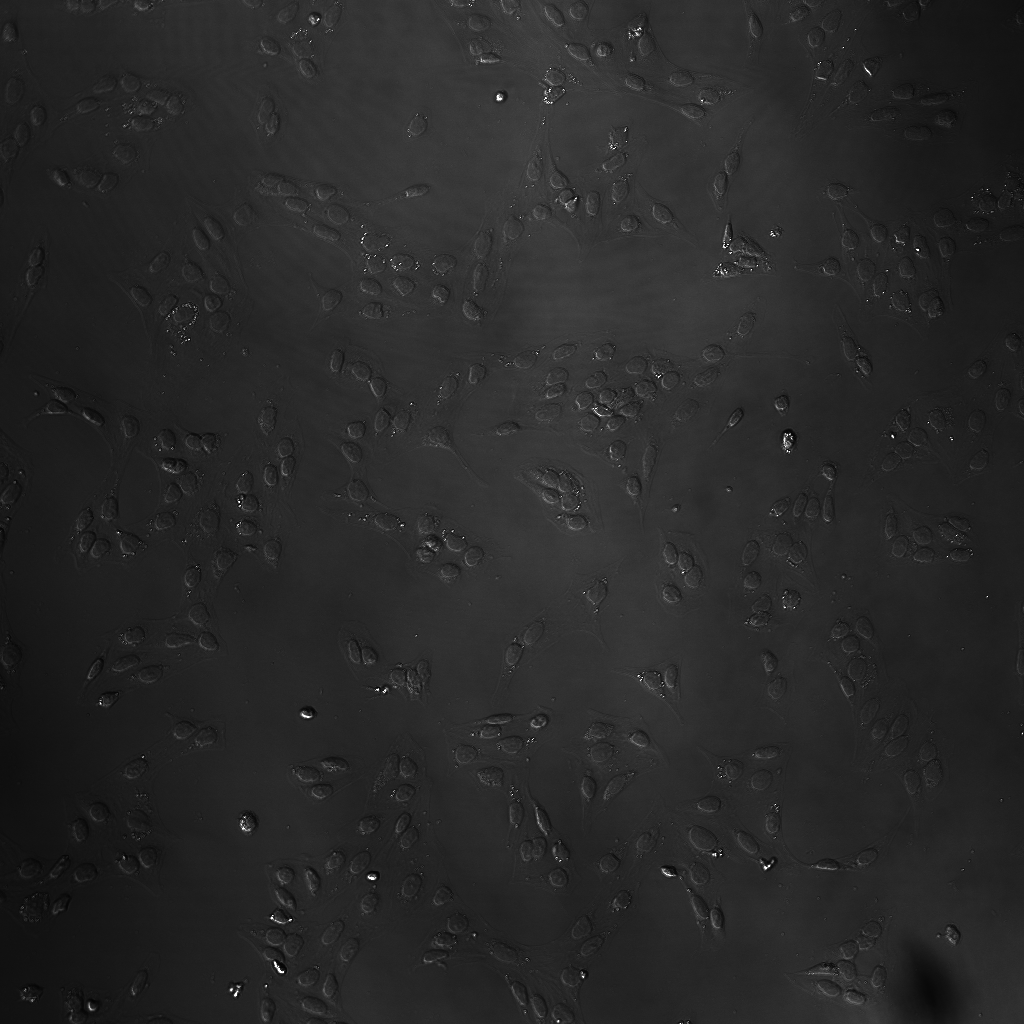

Supplement: S5 File — (ZIP) [file pone.0164217.s005.zip › Some other supplemental files (SOSF)/ICC.TIF/ET/et-200c3.tif]

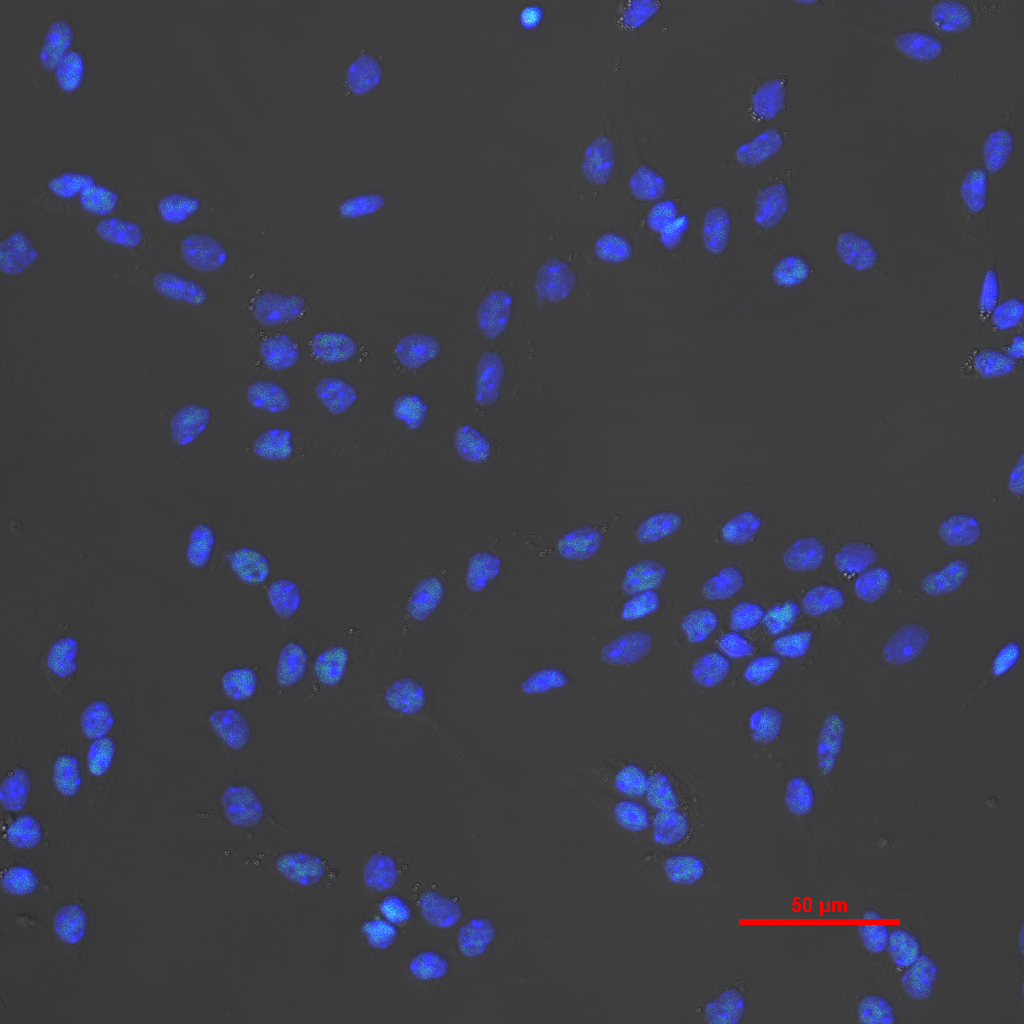

Supplement: S5 File — (ZIP) [file pone.0164217.s005.zip › Some other supplemental files (SOSF)/ICC.TIF/ET/et-400.tif]

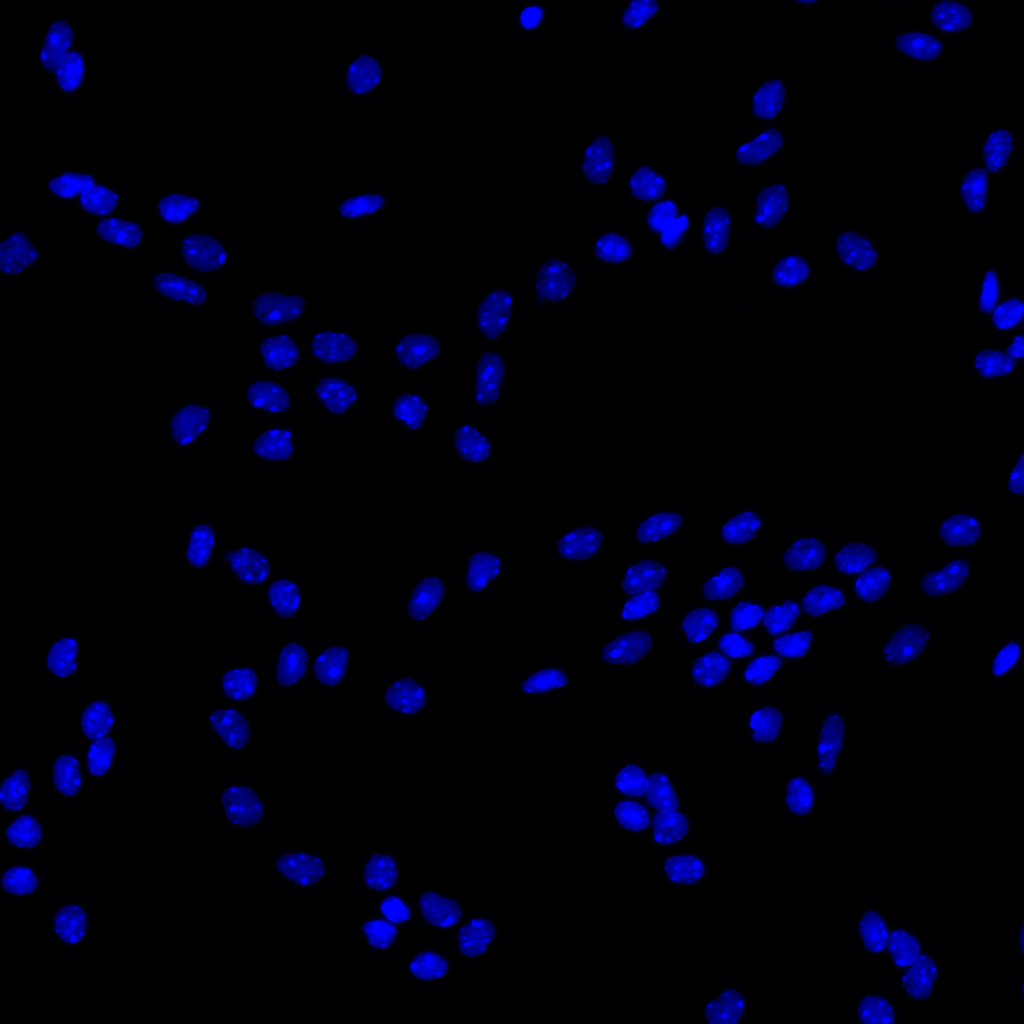

Supplement: S5 File — (ZIP) [file pone.0164217.s005.zip › Some other supplemental files (SOSF)/ICC.TIF/ET/et-400c1.tif]

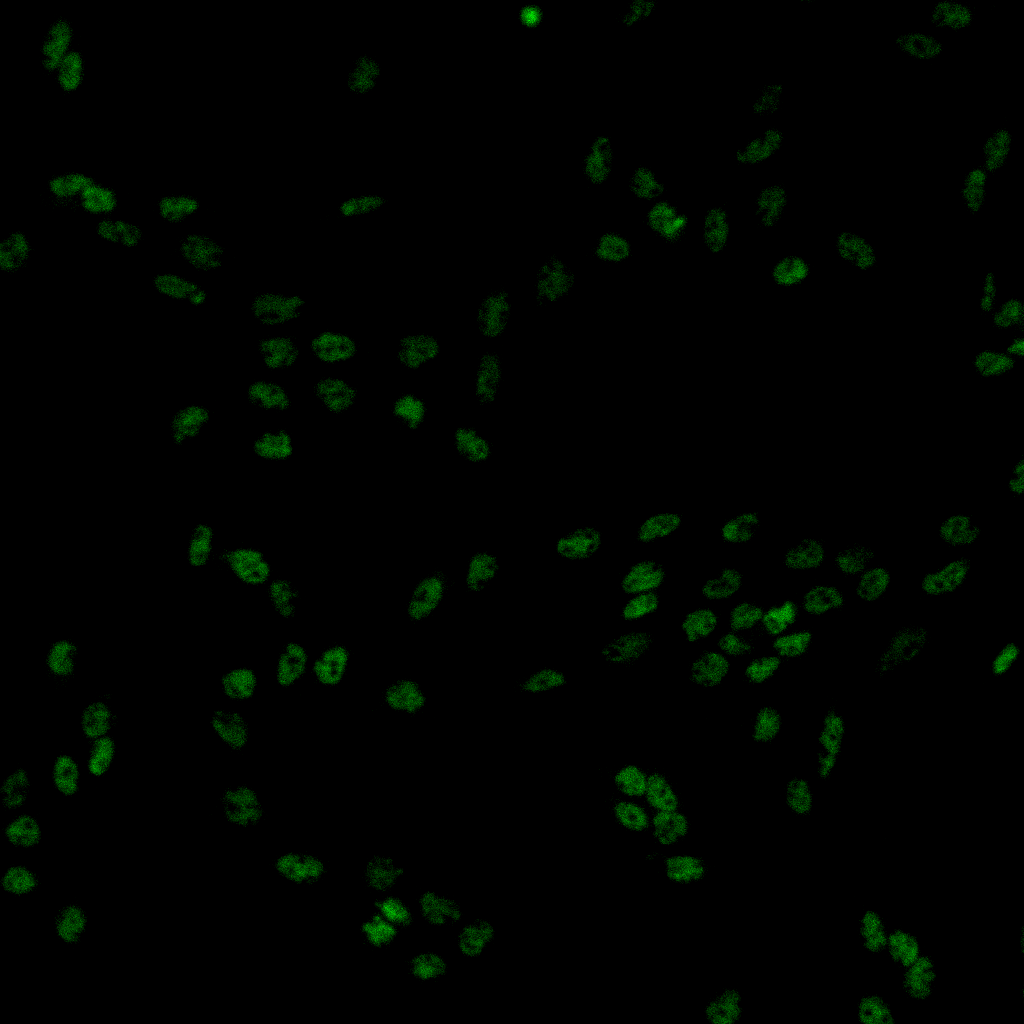

Supplement: S5 File — (ZIP) [file pone.0164217.s005.zip › Some other supplemental files (SOSF)/ICC.TIF/ET/et-400c2.tif]

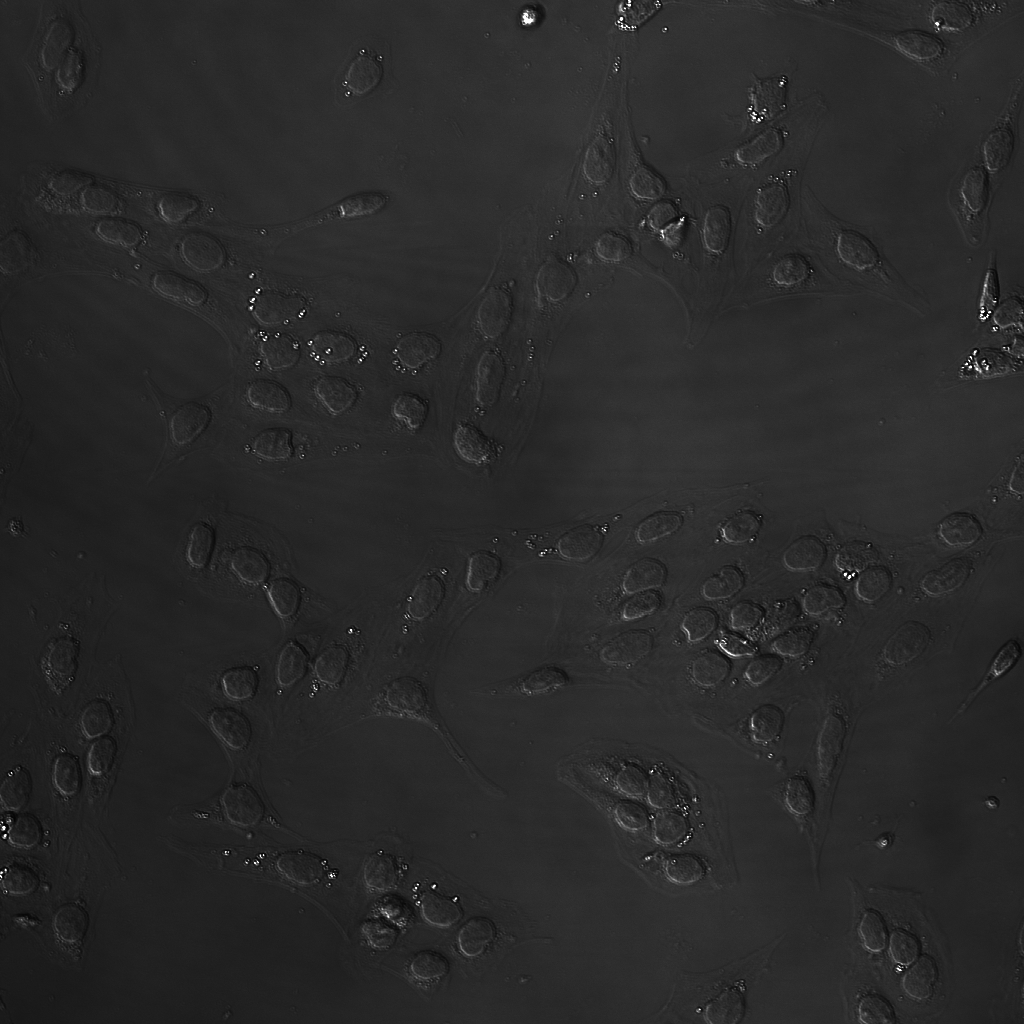

Supplement: S5 File — (ZIP) [file pone.0164217.s005.zip › Some other supplemental files (SOSF)/ICC.TIF/ET/et-400c3.tif]

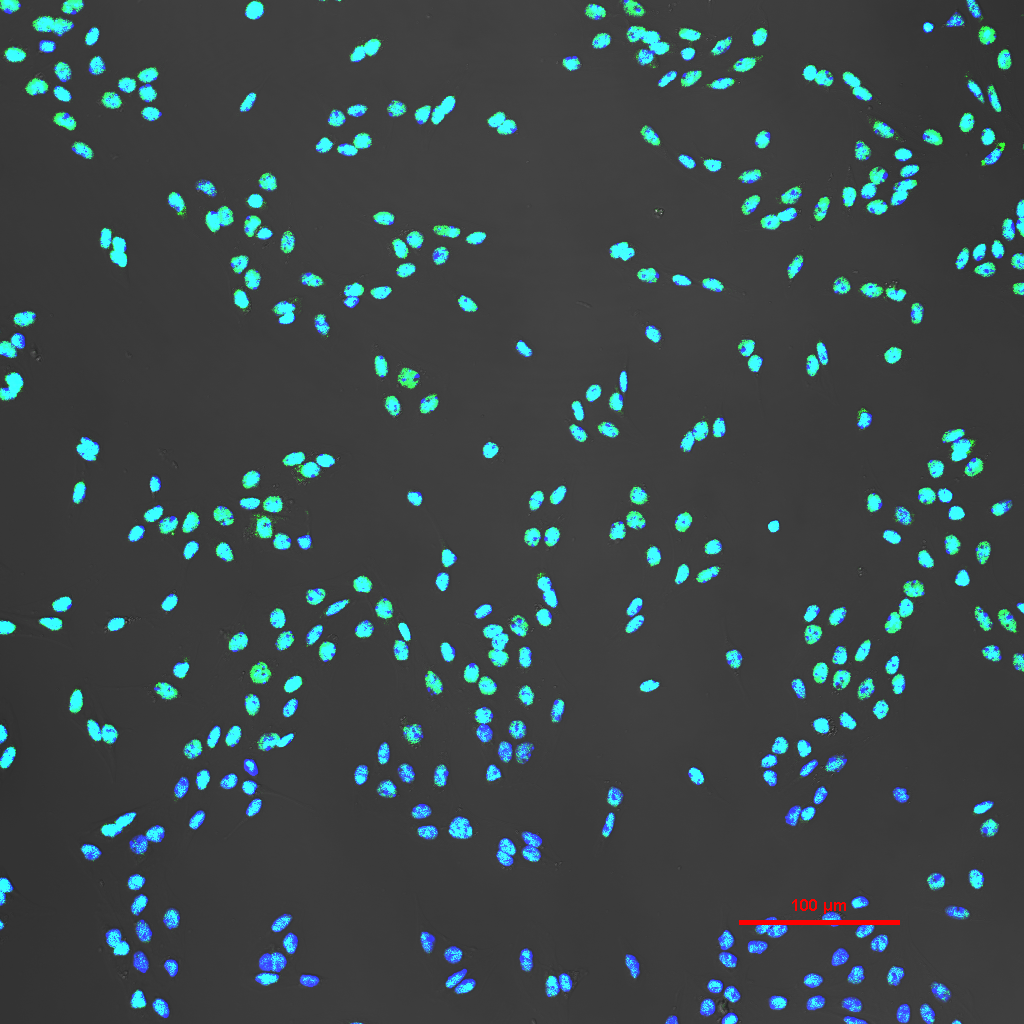

Supplement: S5 File — (ZIP) [file pone.0164217.s005.zip › Some other supplemental files (SOSF)/ICC.TIF/NET/net-200.tif]

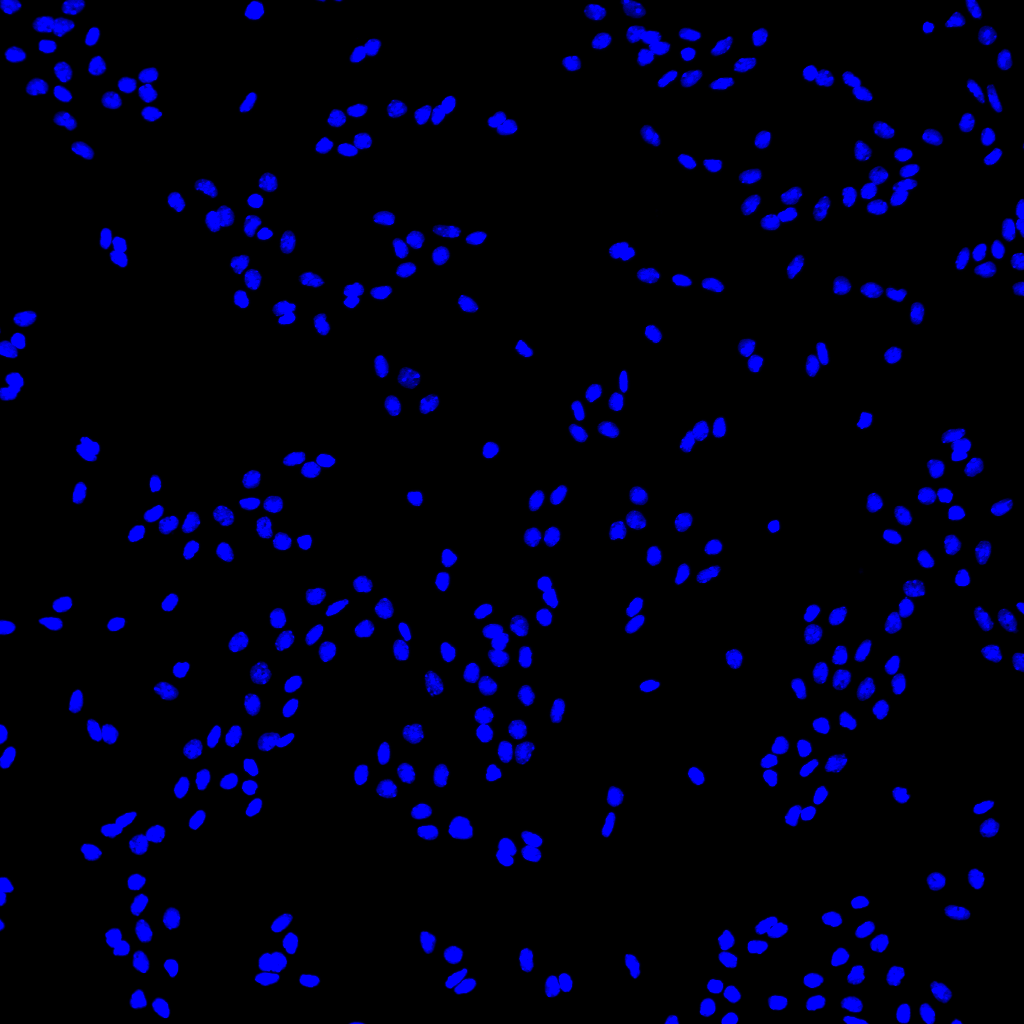

Supplement: S5 File — (ZIP) [file pone.0164217.s005.zip › Some other supplemental files (SOSF)/ICC.TIF/NET/net-200c1.tif]

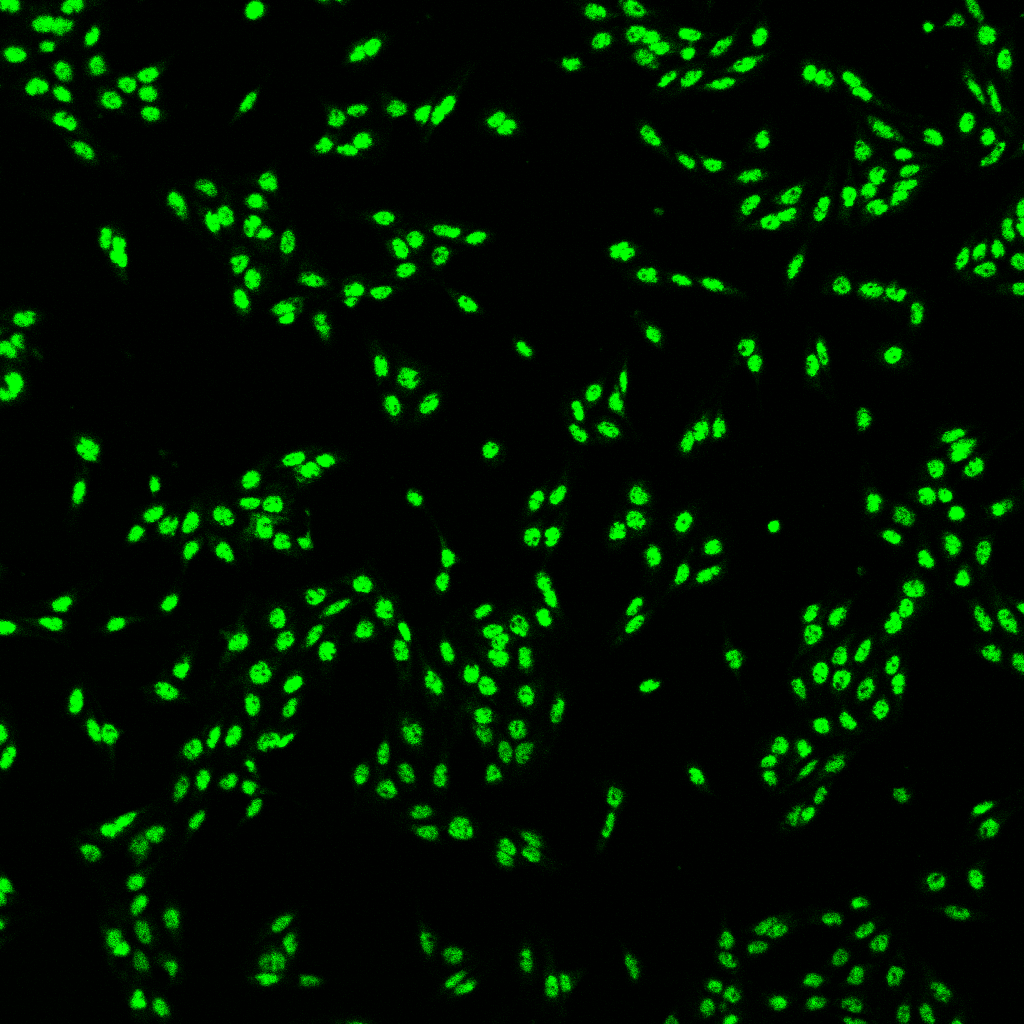

Supplement: S5 File — (ZIP) [file pone.0164217.s005.zip › Some other supplemental files (SOSF)/ICC.TIF/NET/net-200c2.tif]

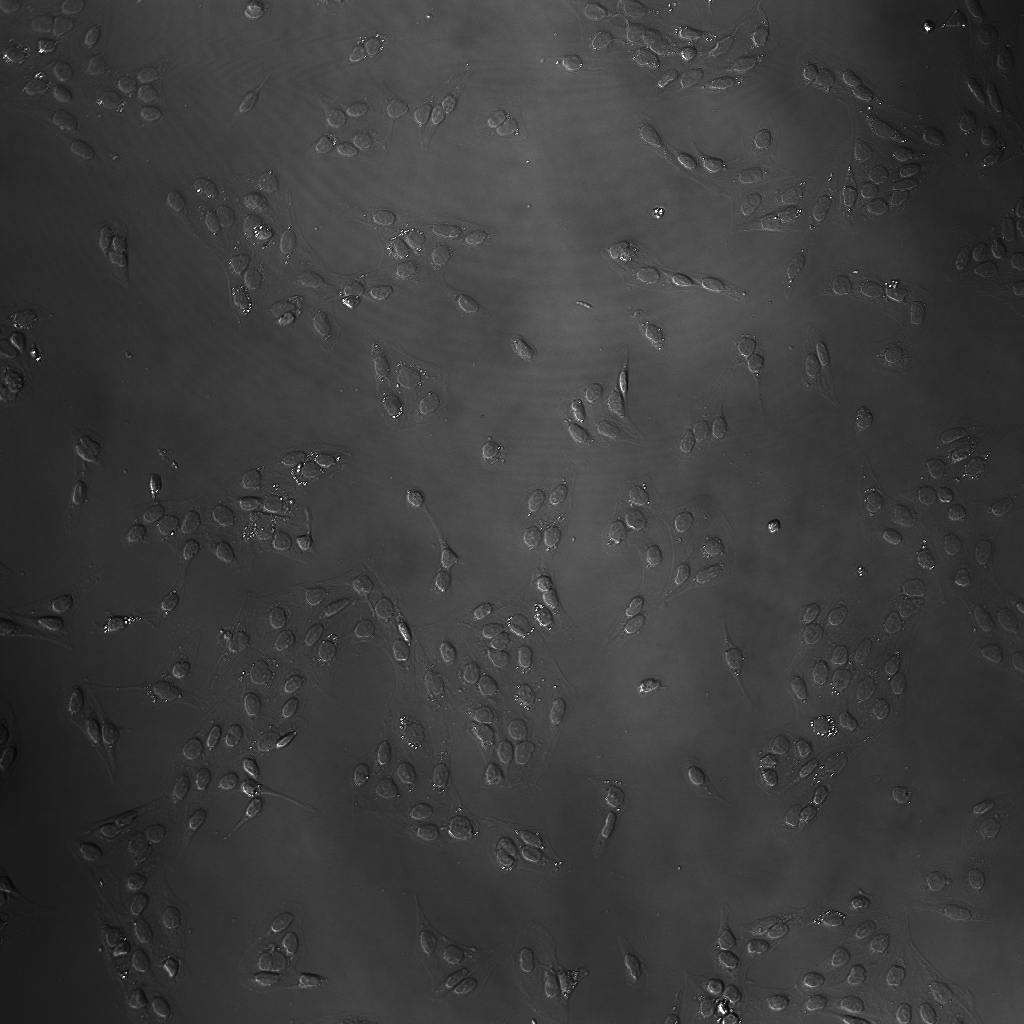

Supplement: S5 File — (ZIP) [file pone.0164217.s005.zip › Some other supplemental files (SOSF)/ICC.TIF/NET/net-200c3.tif]

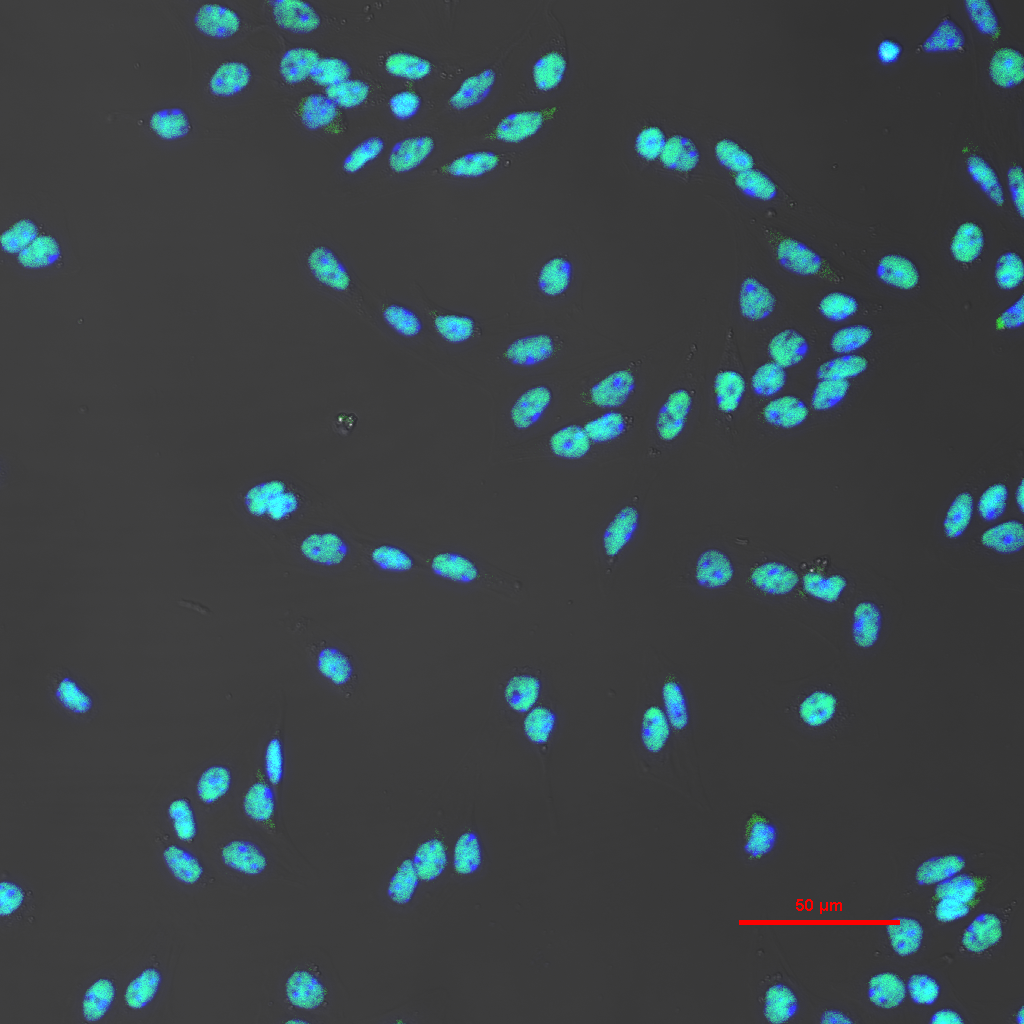

Supplement: S5 File — (ZIP) [file pone.0164217.s005.zip › Some other supplemental files (SOSF)/ICC.TIF/NET/net-400.tif]

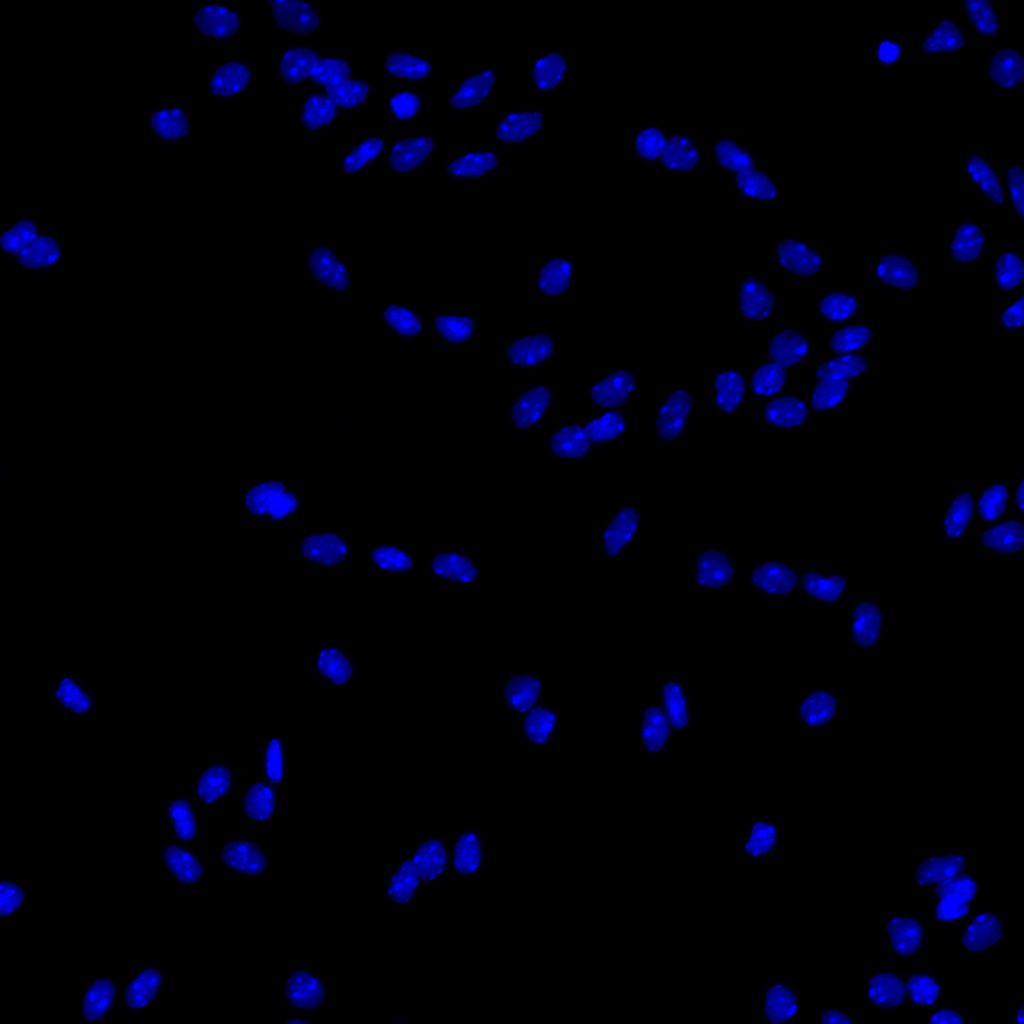

Supplement: S5 File — (ZIP) [file pone.0164217.s005.zip › Some other supplemental files (SOSF)/ICC.TIF/NET/net-400c1.tif]

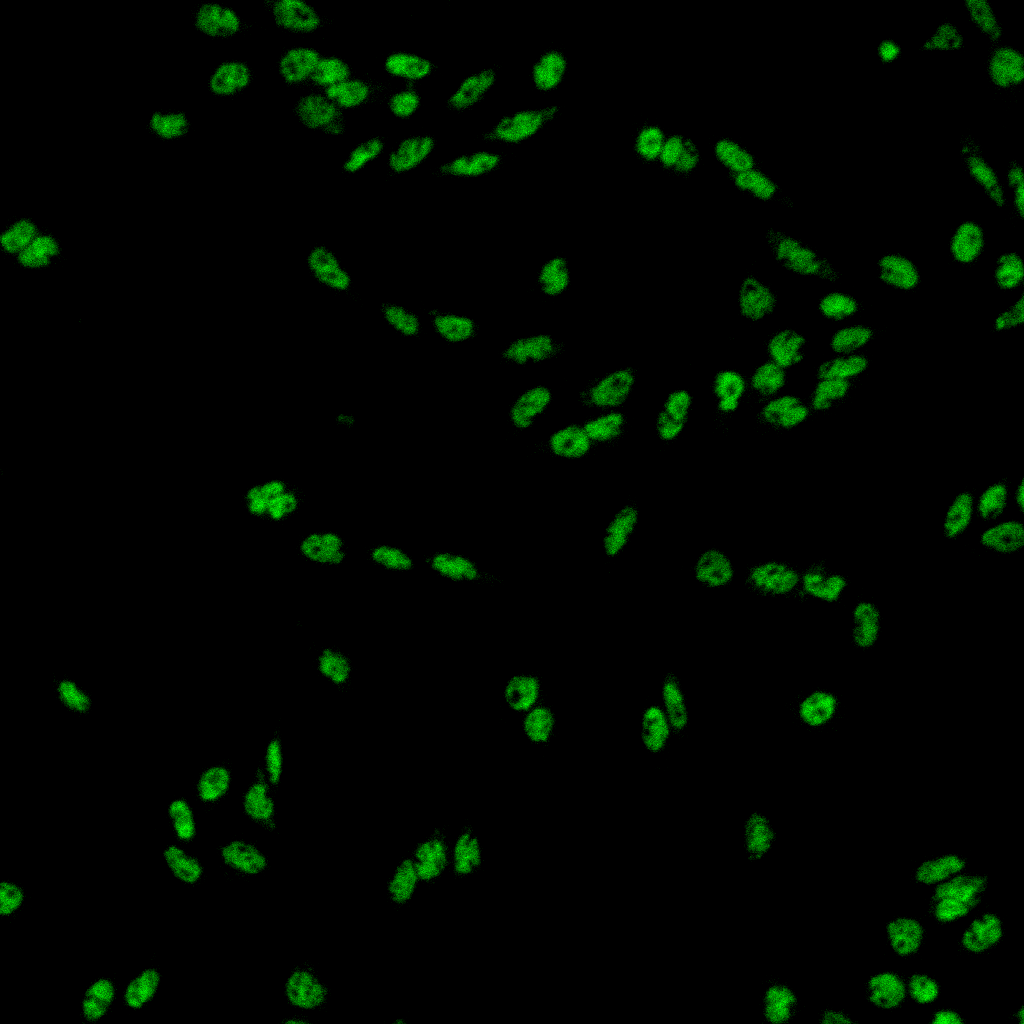

Supplement: S5 File — (ZIP) [file pone.0164217.s005.zip › Some other supplemental files (SOSF)/ICC.TIF/NET/net-400c2.tif]

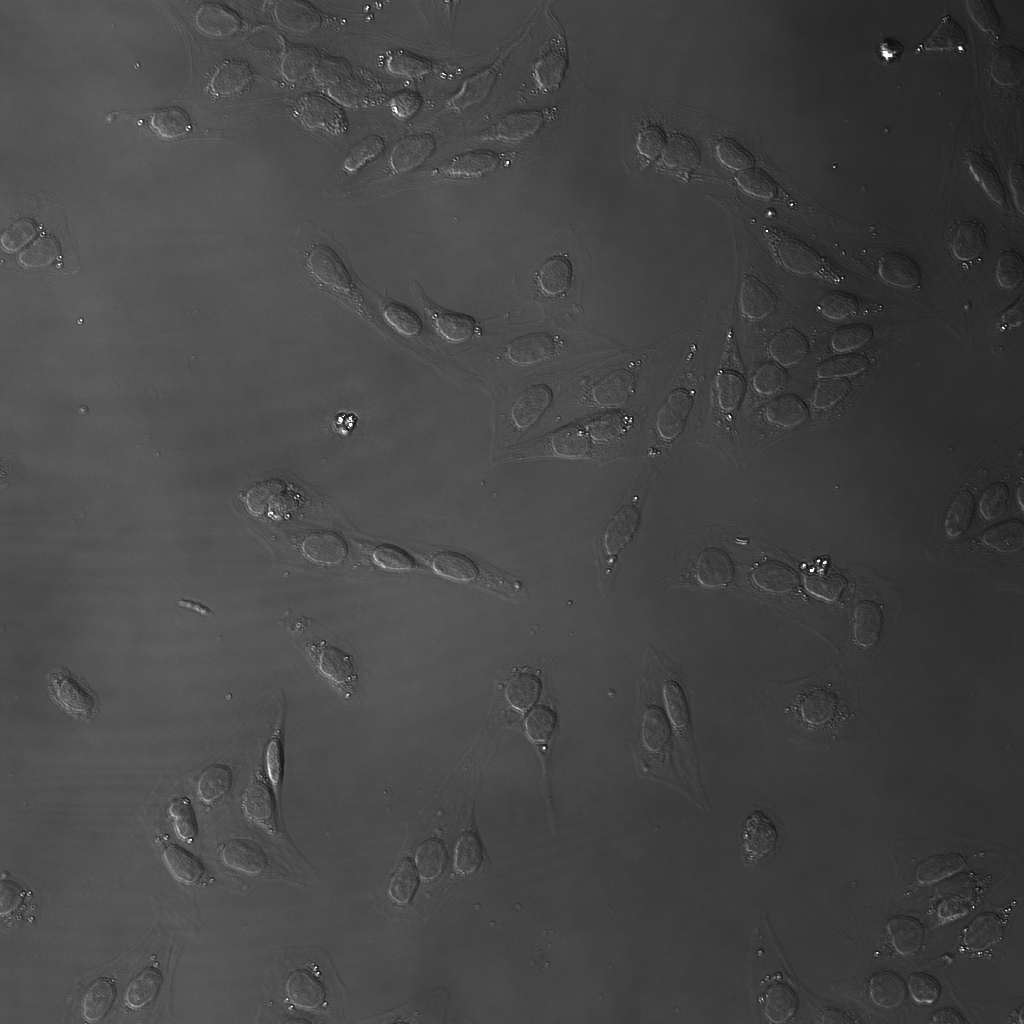

Supplement: S5 File — (ZIP) [file pone.0164217.s005.zip › Some other supplemental files (SOSF)/ICC.TIF/NET/net-400c3.tif]

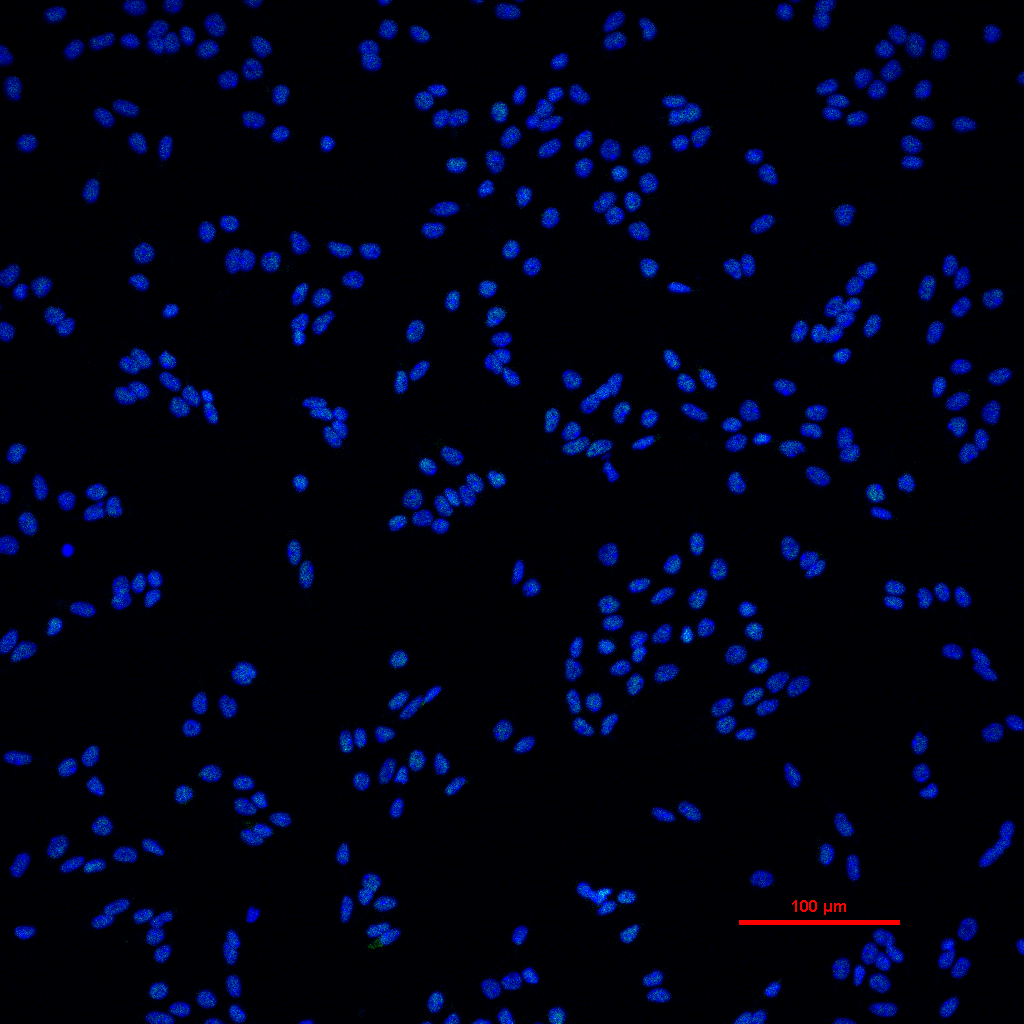

Supplement: S5 File — (ZIP) [file pone.0164217.s005.zip › Some other supplemental files (SOSF)/ICC.TIF/NT-ET/nt-et-200.tif]

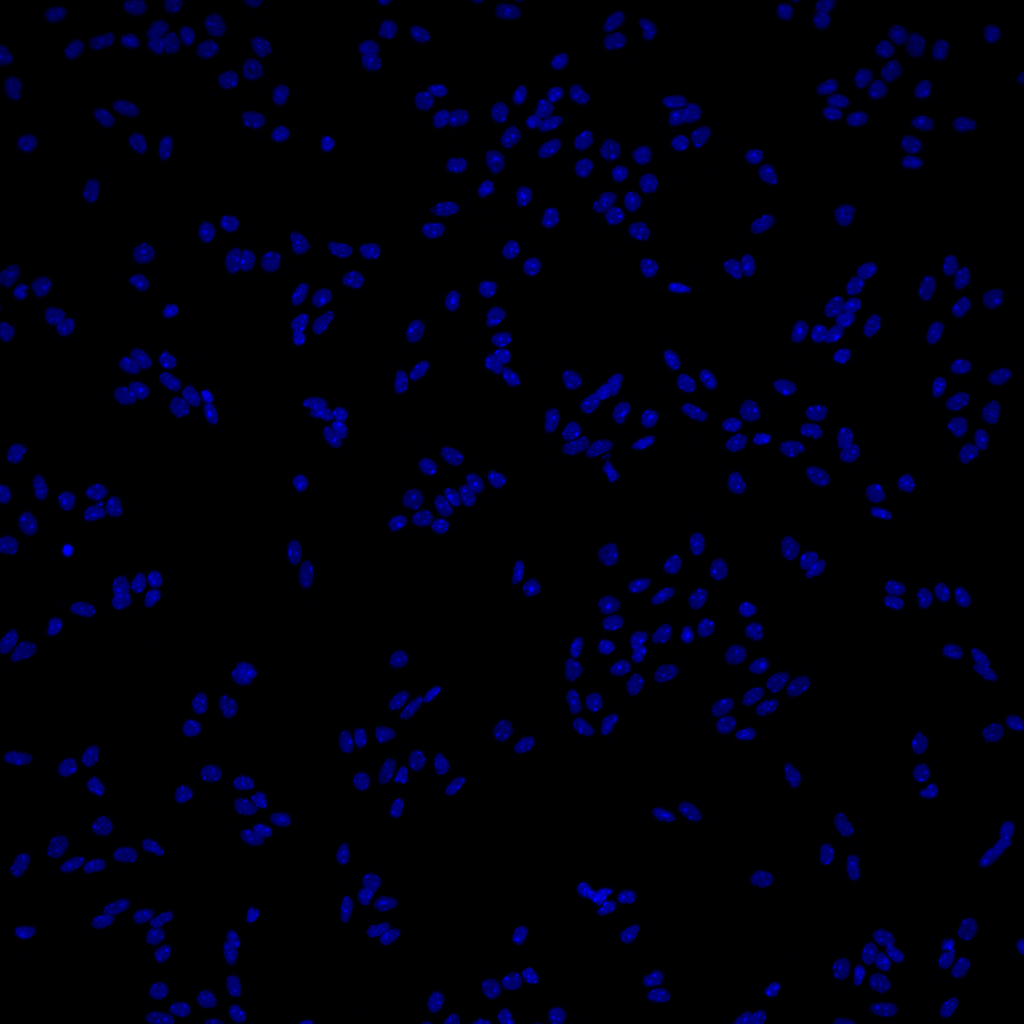

Supplement: S5 File — (ZIP) [file pone.0164217.s005.zip › Some other supplemental files (SOSF)/ICC.TIF/NT-ET/nt-et-200c1.tif]

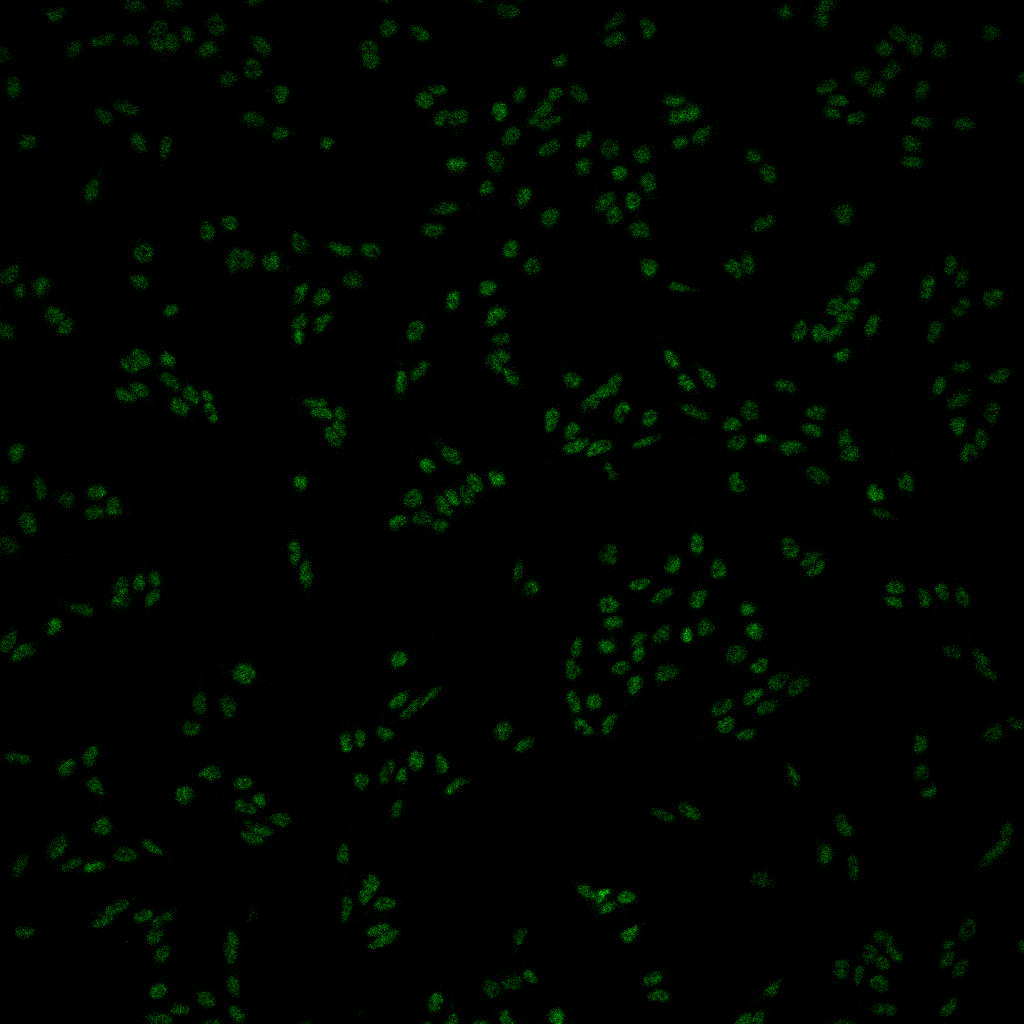

Supplement: S5 File — (ZIP) [file pone.0164217.s005.zip › Some other supplemental files (SOSF)/ICC.TIF/NT-ET/nt-et-200c2.tif]

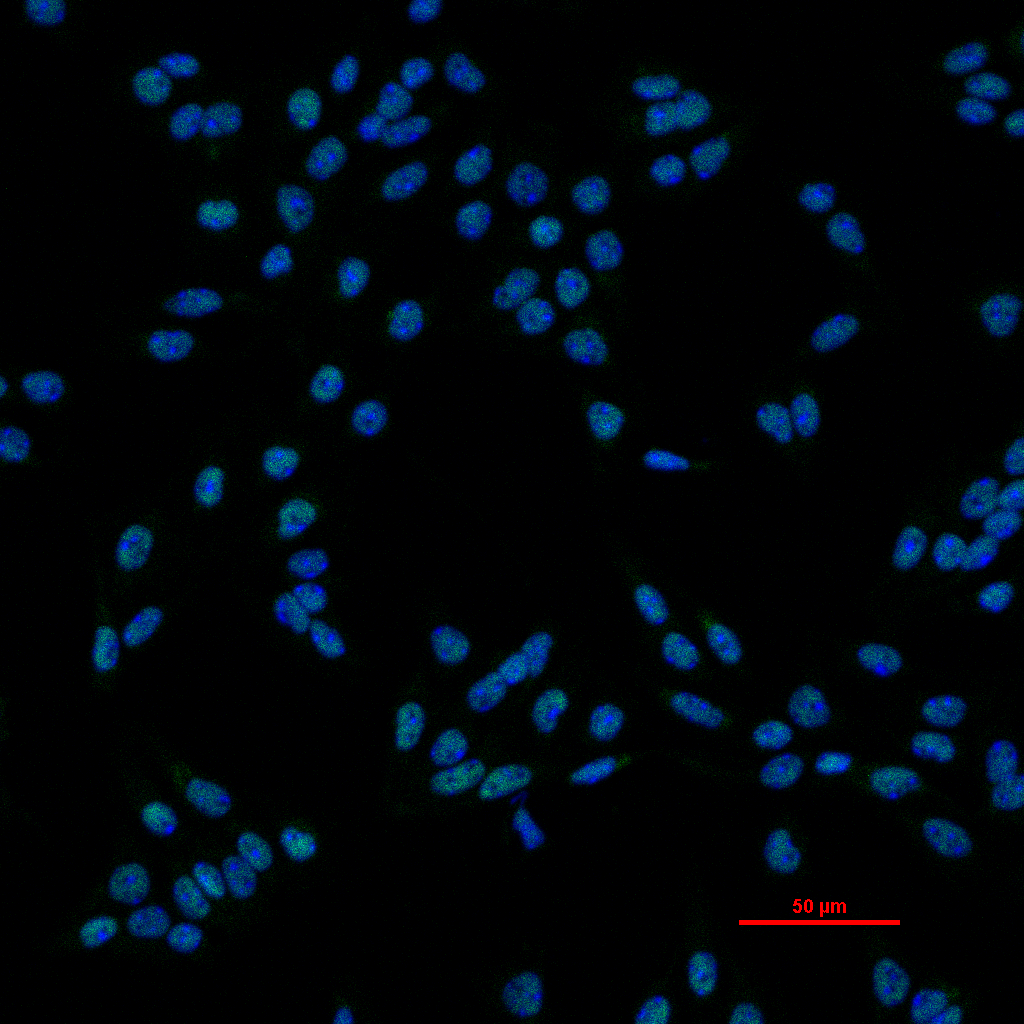

Supplement: S5 File — (ZIP) [file pone.0164217.s005.zip › Some other supplemental files (SOSF)/ICC.TIF/NT-ET/nt-et-400.tif]

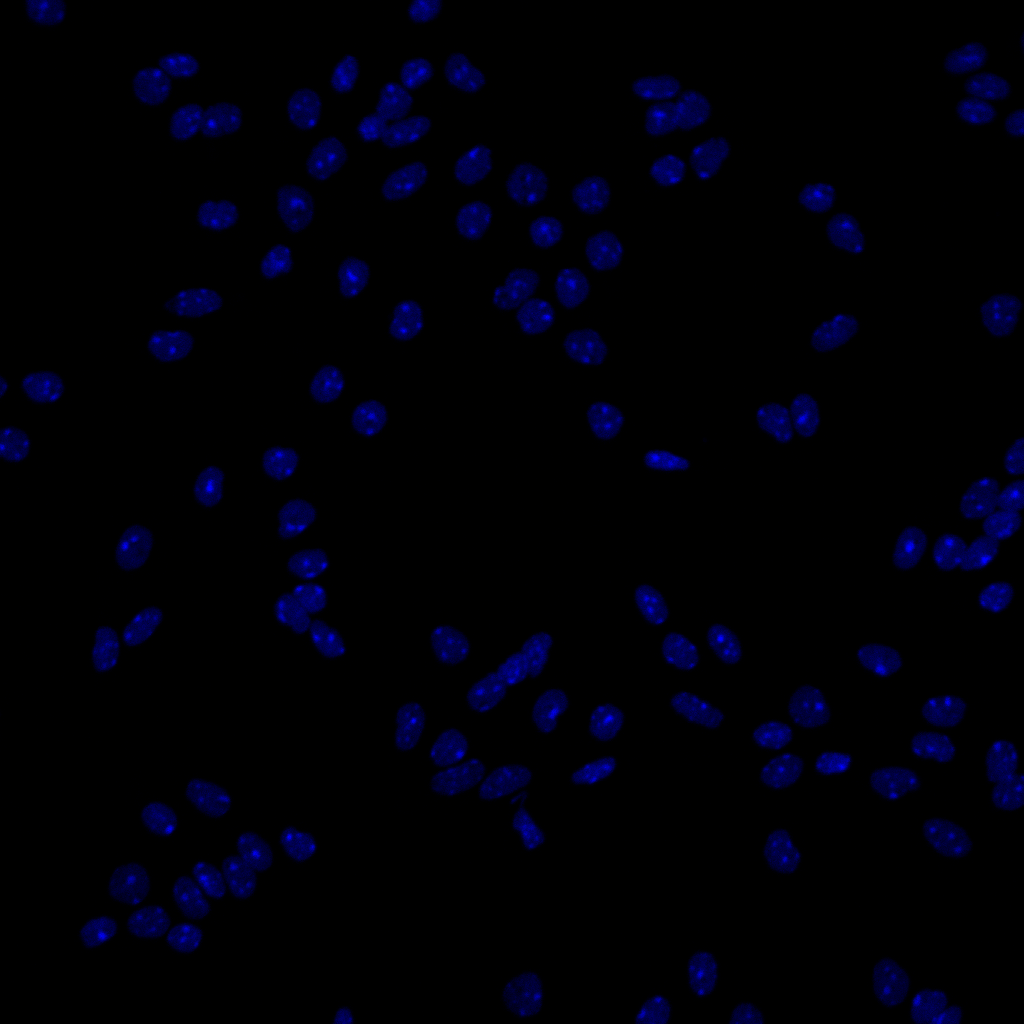

Supplement: S5 File — (ZIP) [file pone.0164217.s005.zip › Some other supplemental files (SOSF)/ICC.TIF/NT-ET/nt-et-400c1.tif]

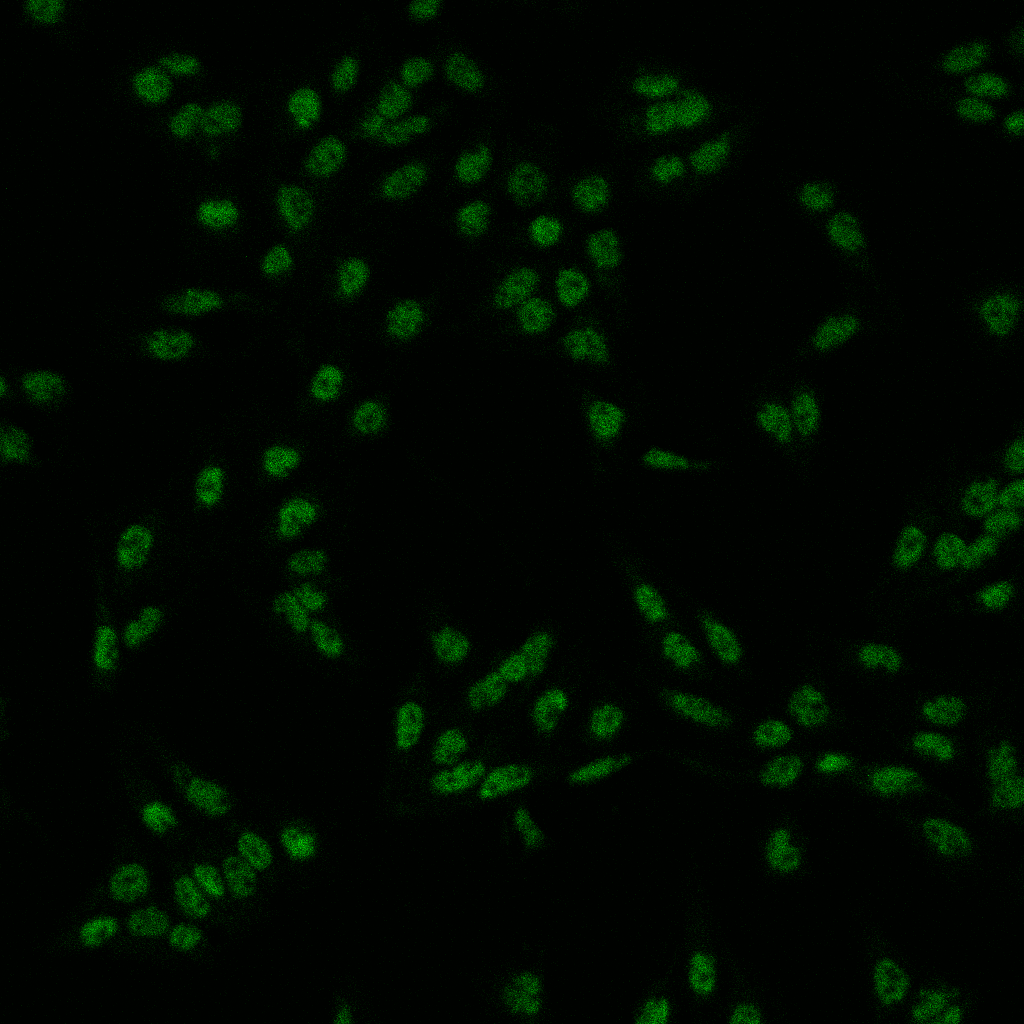

Supplement: S5 File — (ZIP) [file pone.0164217.s005.zip › Some other supplemental files (SOSF)/ICC.TIF/NT-ET/nt-et-400c2.tif]

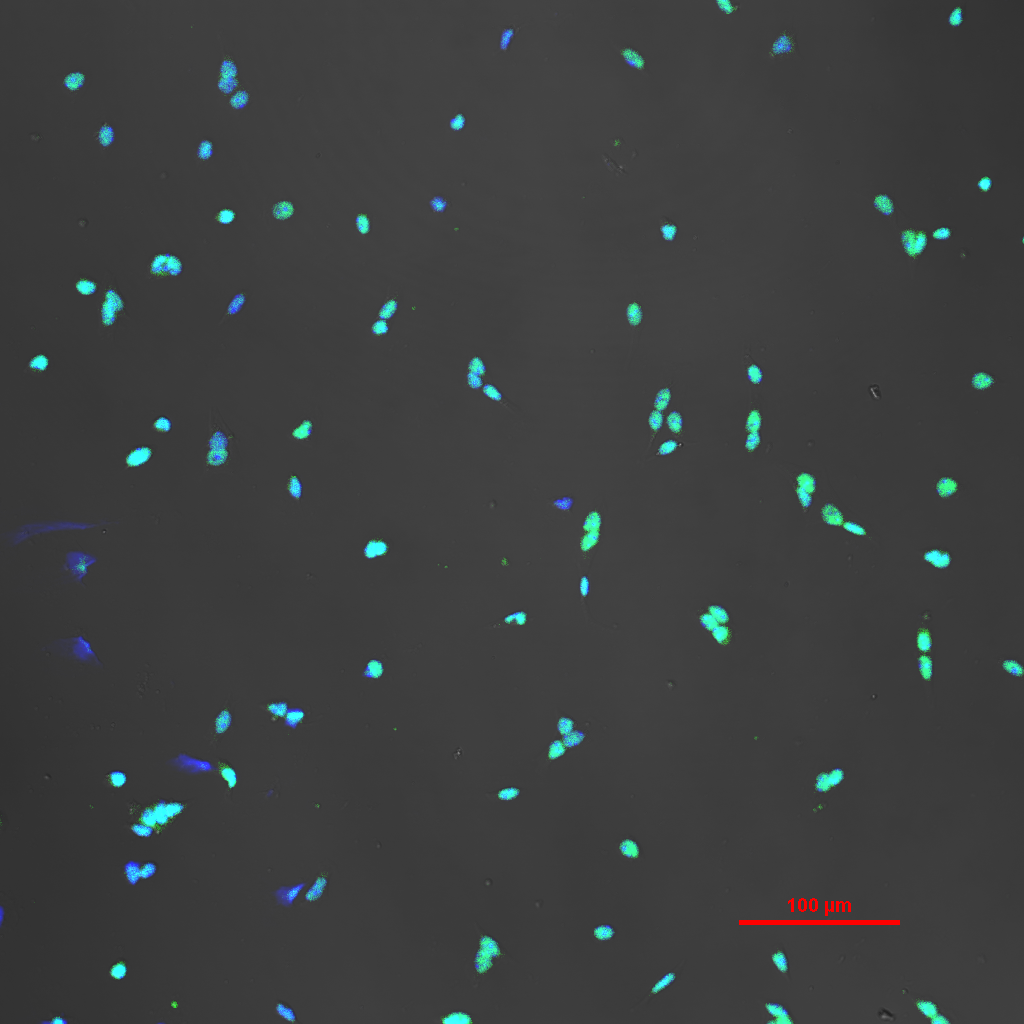

Supplement: S5 File — (ZIP) [file pone.0164217.s005.zip › Some other supplemental files (SOSF)/ICC.TIF/NT-NET/nt-net-200.tif]

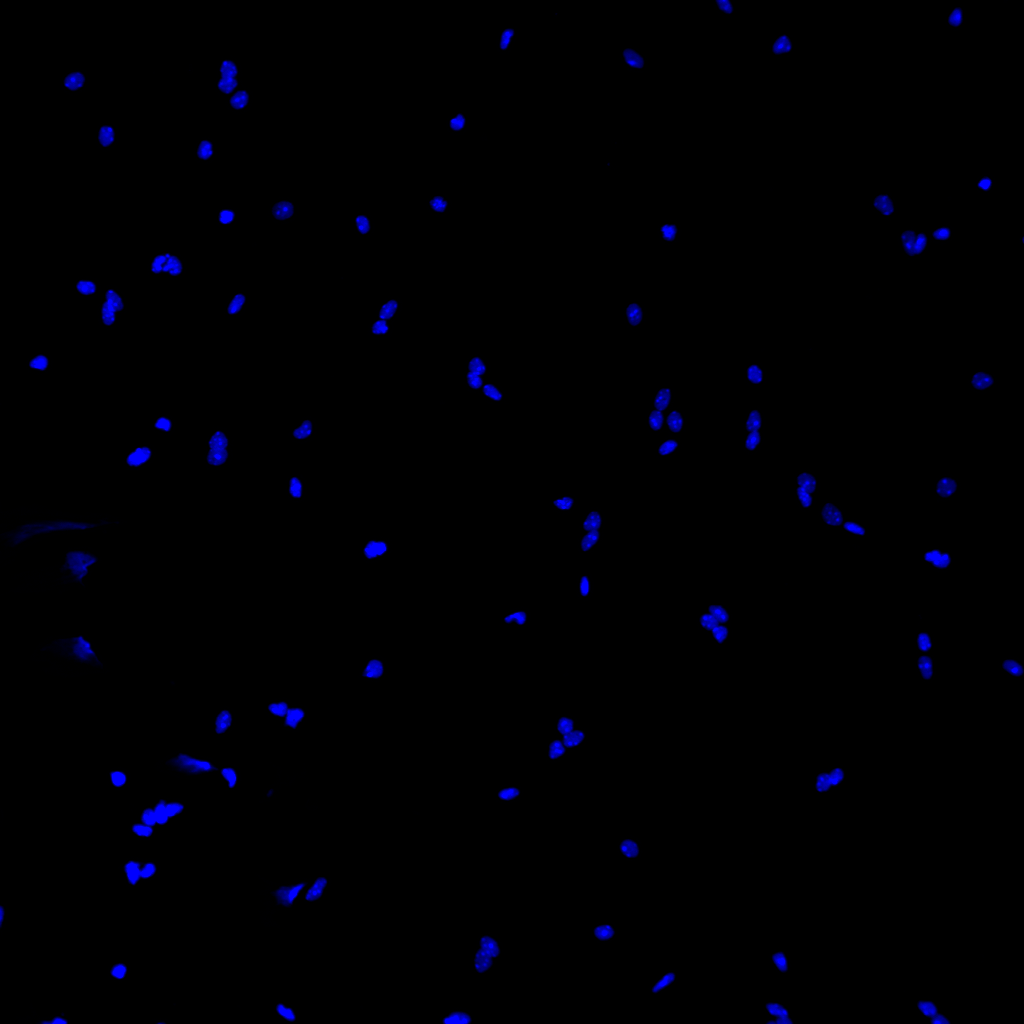

Supplement: S5 File — (ZIP) [file pone.0164217.s005.zip › Some other supplemental files (SOSF)/ICC.TIF/NT-NET/nt-net-200c1.tif]

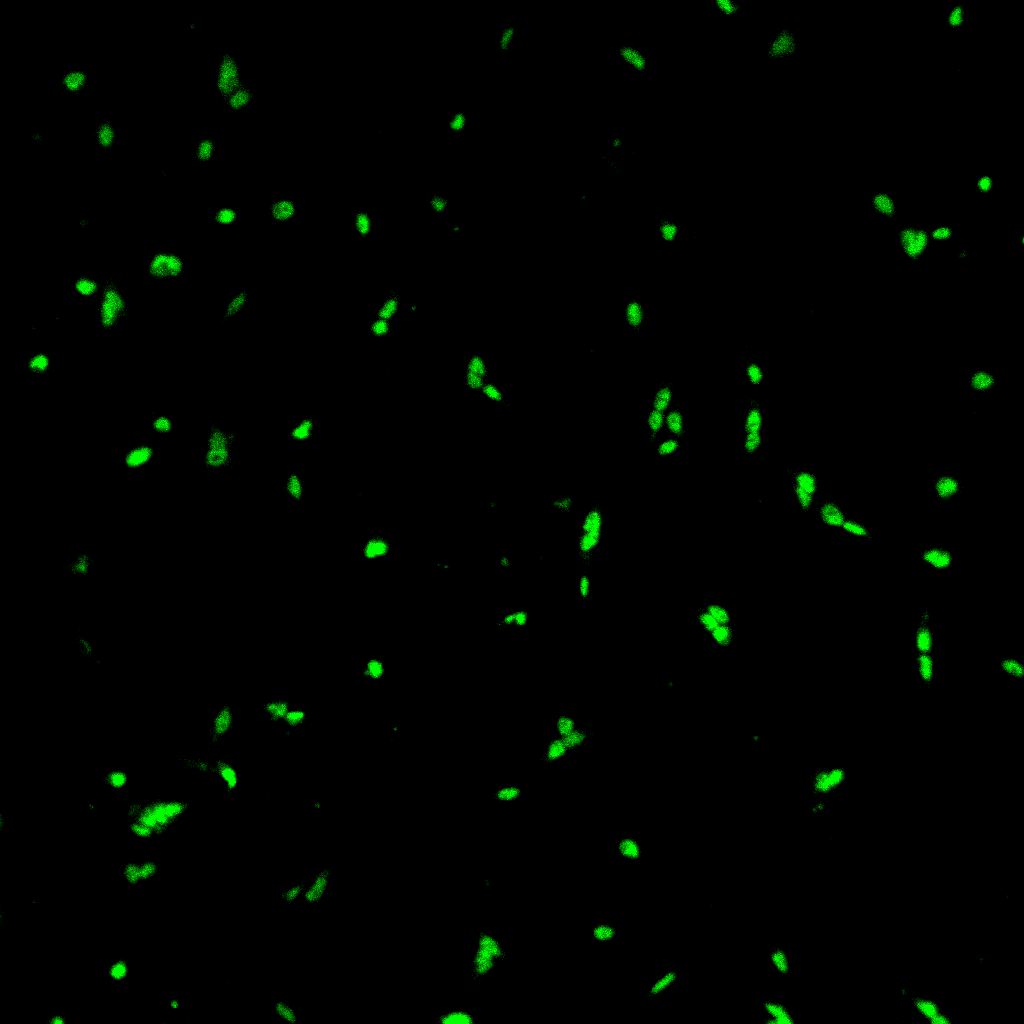

Supplement: S5 File — (ZIP) [file pone.0164217.s005.zip › Some other supplemental files (SOSF)/ICC.TIF/NT-NET/nt-net-200c2.tif]

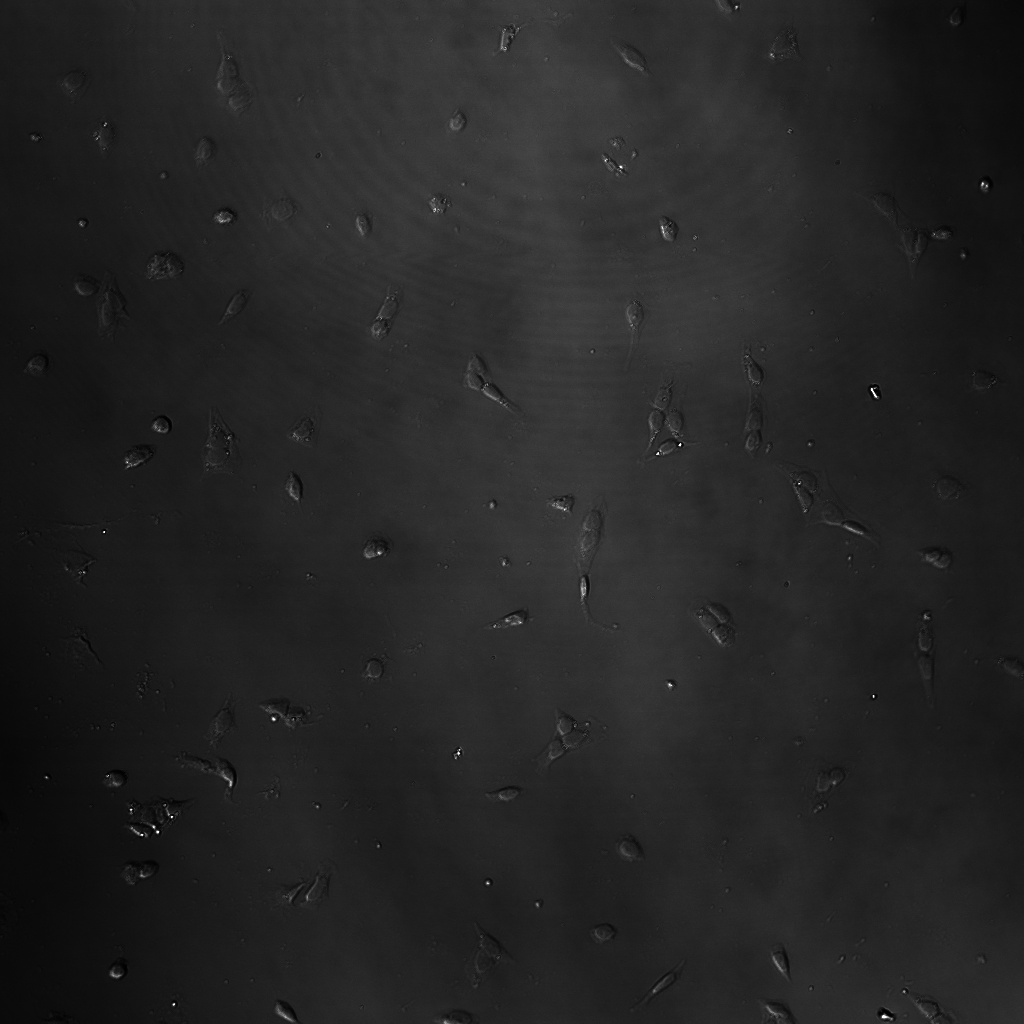

Supplement: S5 File — (ZIP) [file pone.0164217.s005.zip › Some other supplemental files (SOSF)/ICC.TIF/NT-NET/nt-net-200c3.tif]

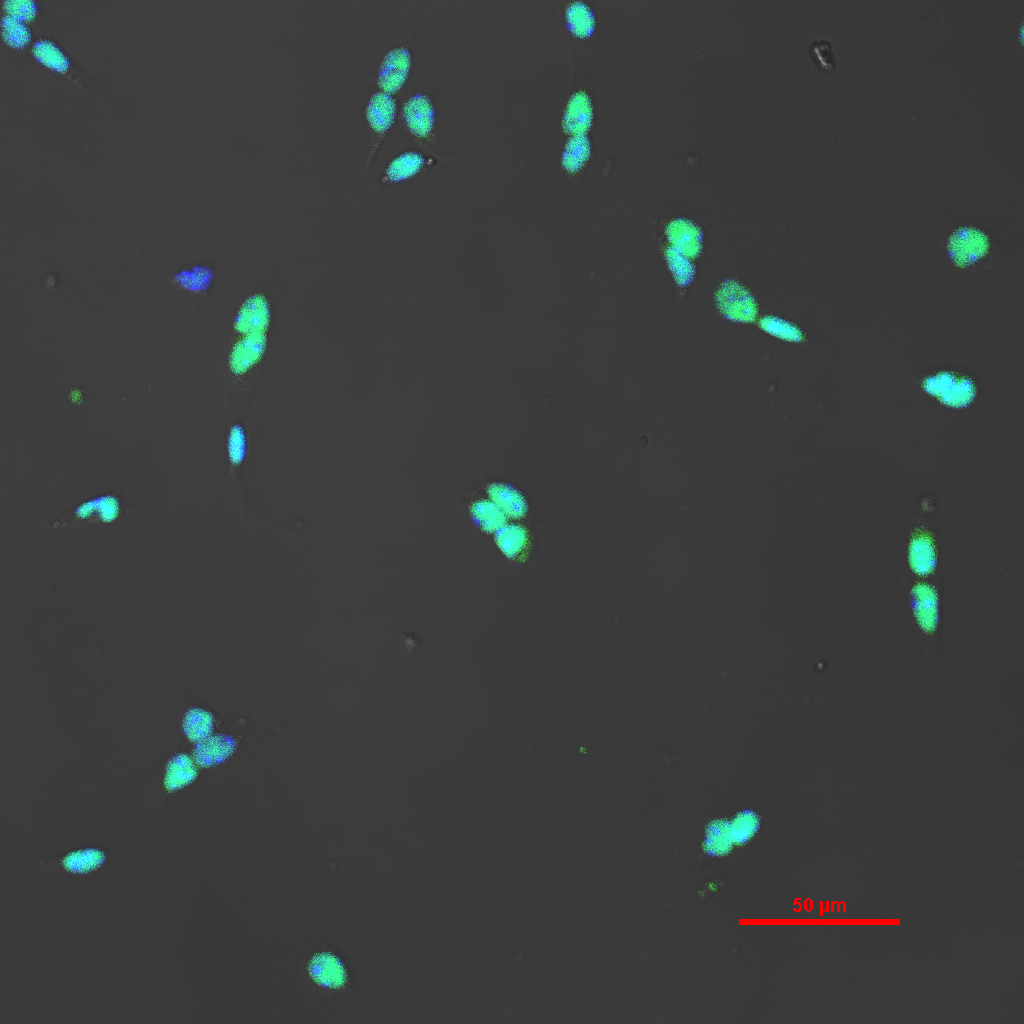

Supplement: S5 File — (ZIP) [file pone.0164217.s005.zip › Some other supplemental files (SOSF)/ICC.TIF/NT-NET/nt-net-400.tif]

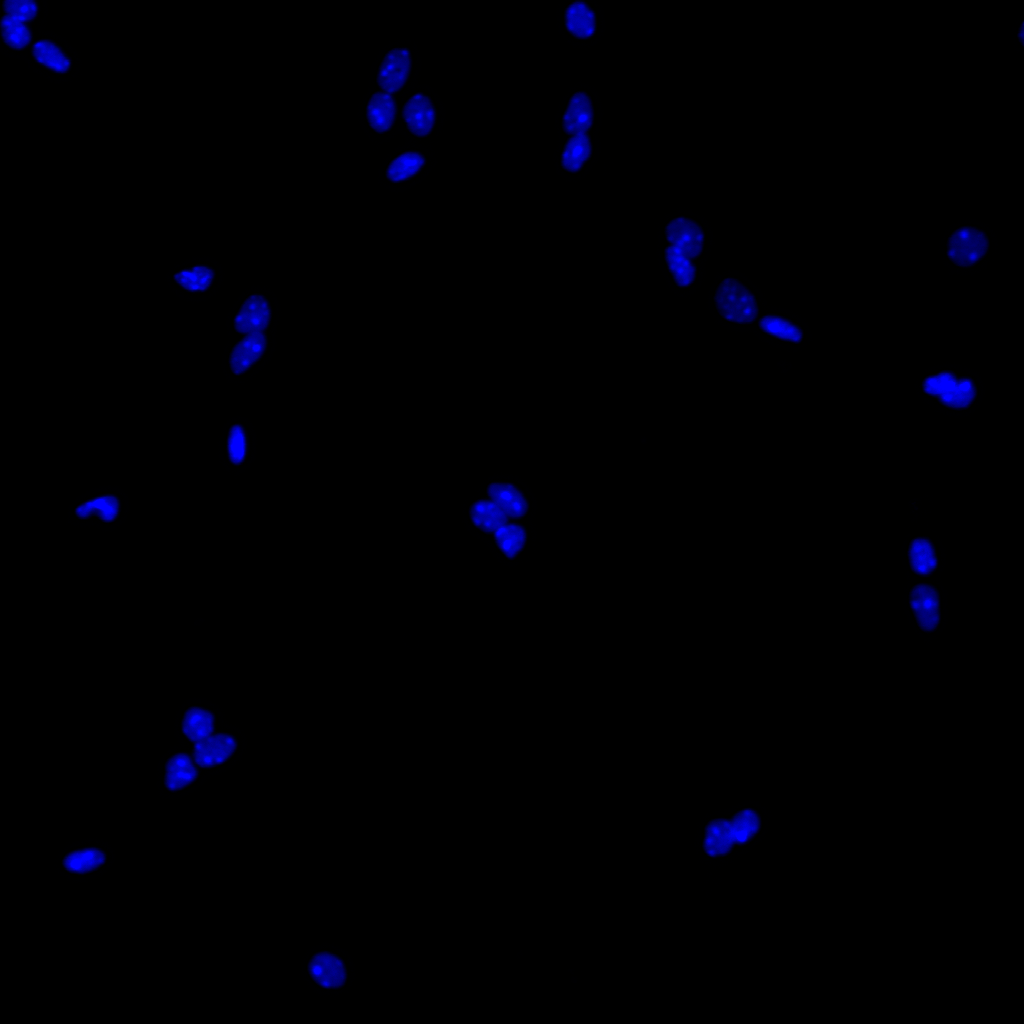

Supplement: S5 File — (ZIP) [file pone.0164217.s005.zip › Some other supplemental files (SOSF)/ICC.TIF/NT-NET/nt-net-400c1.tif]

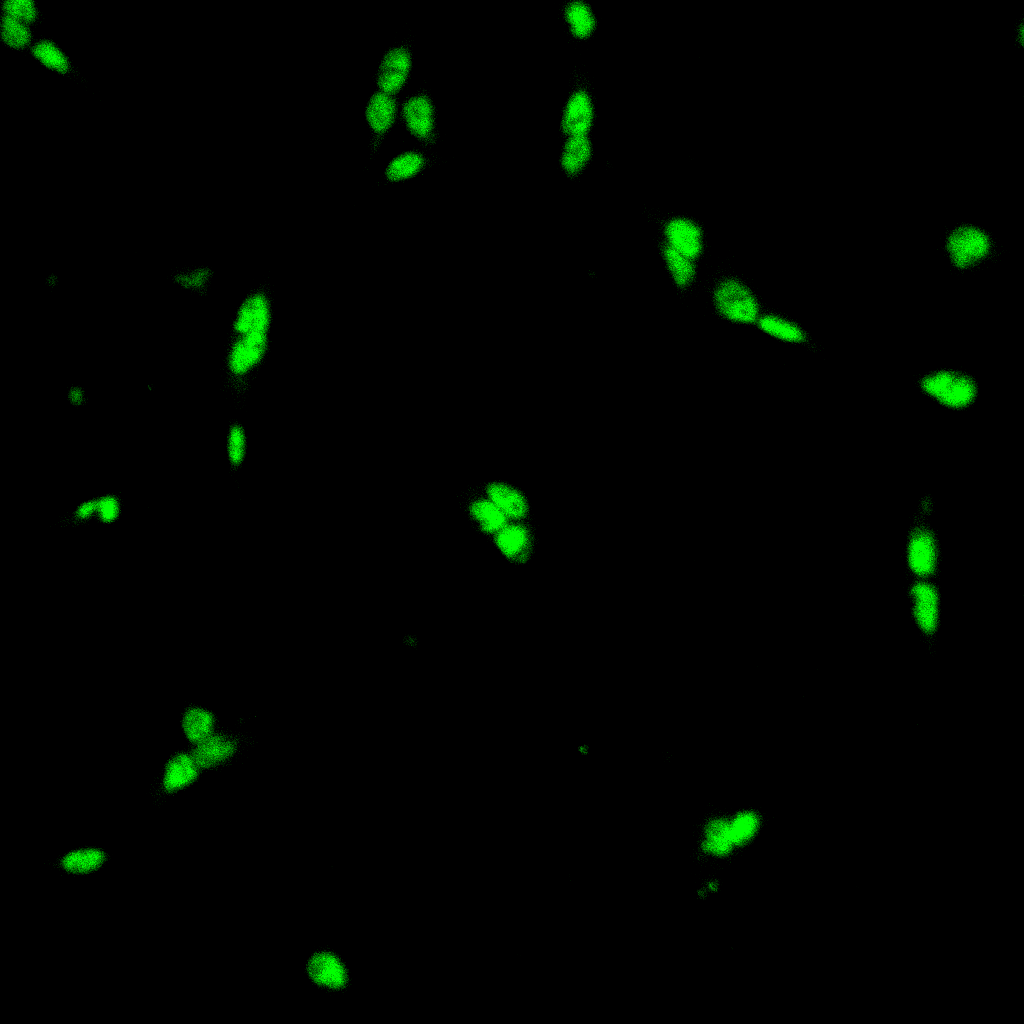

Supplement: S5 File — (ZIP) [file pone.0164217.s005.zip › Some other supplemental files (SOSF)/ICC.TIF/NT-NET/nt-net-400c2.tif]

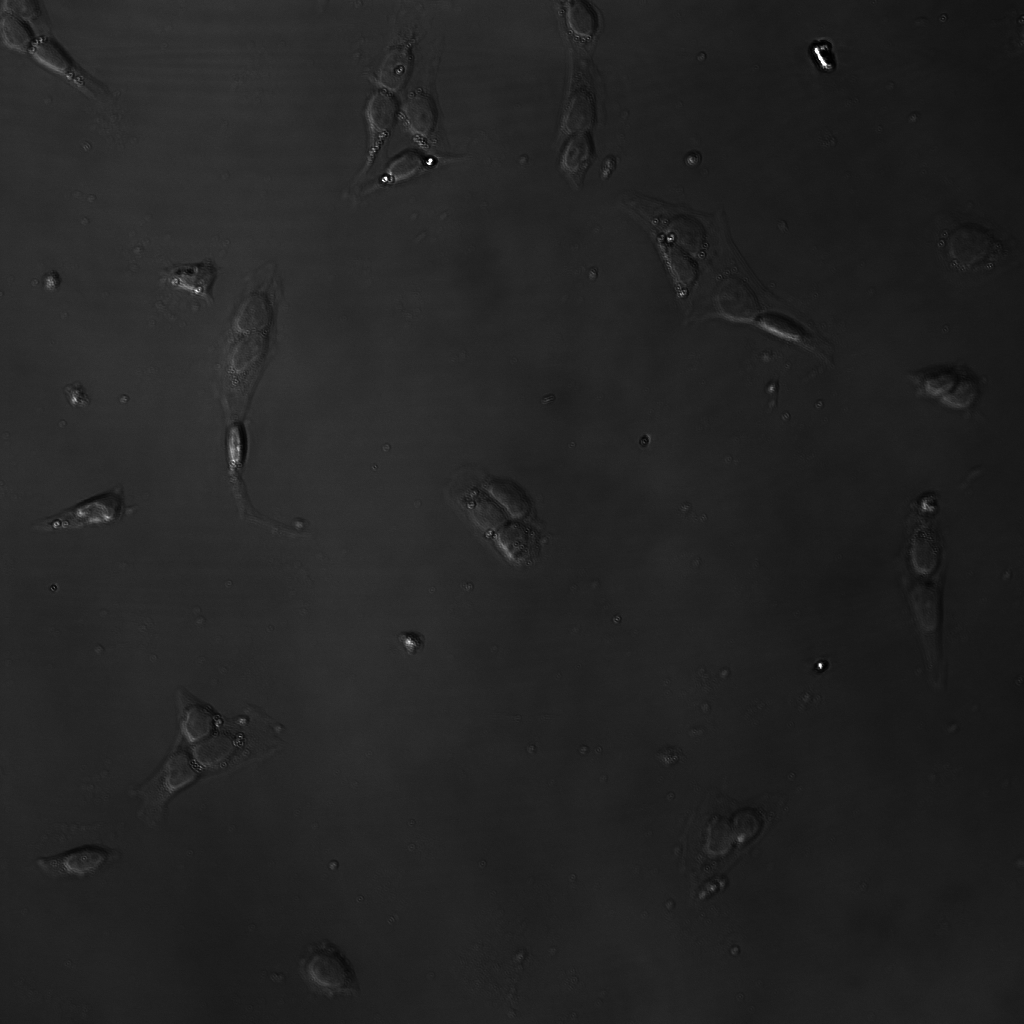

Supplement: S5 File — (ZIP) [file pone.0164217.s005.zip › Some other supplemental files (SOSF)/ICC.TIF/NT-NET/nt-net-400c3.tif]

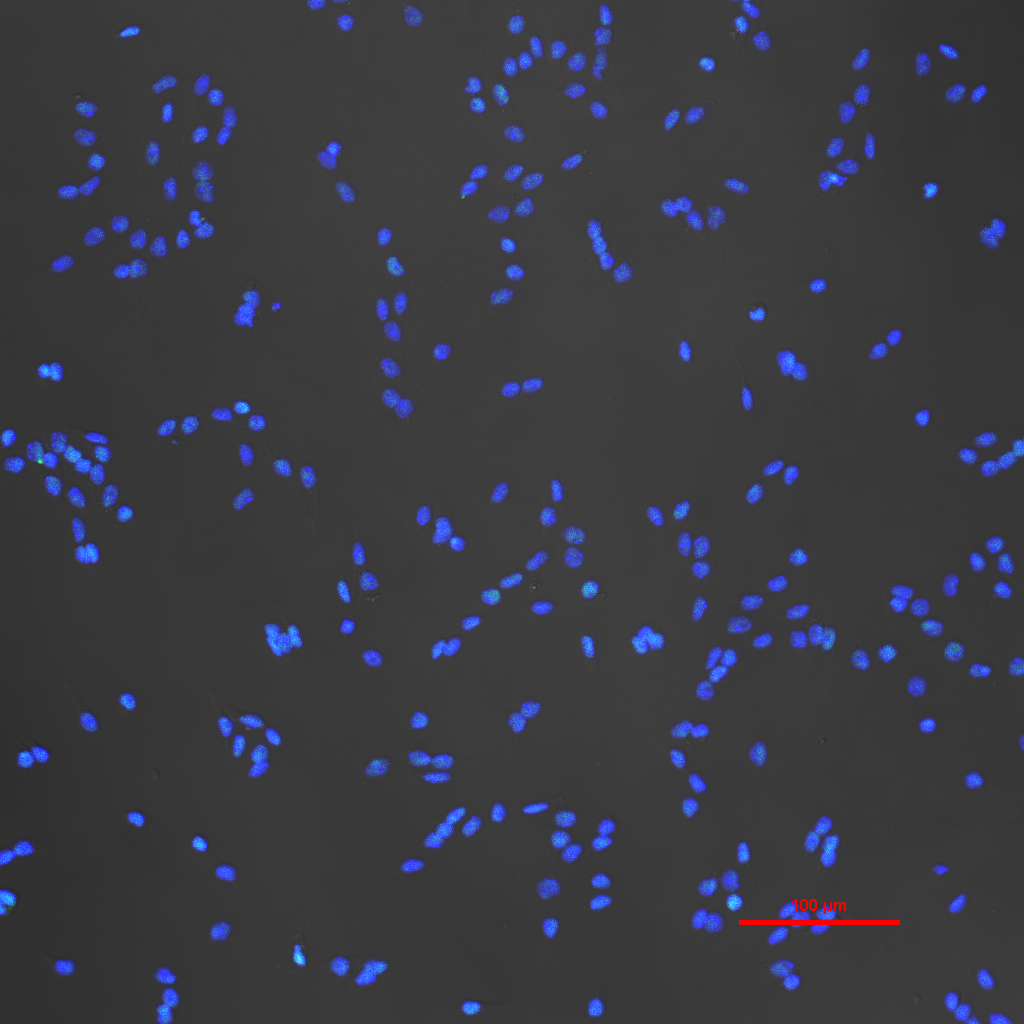

Supplement: S5 File — (ZIP) [file pone.0164217.s005.zip › Some other supplemental files (SOSF)/ICC.TIF/T-ET/t-et-200.tif]

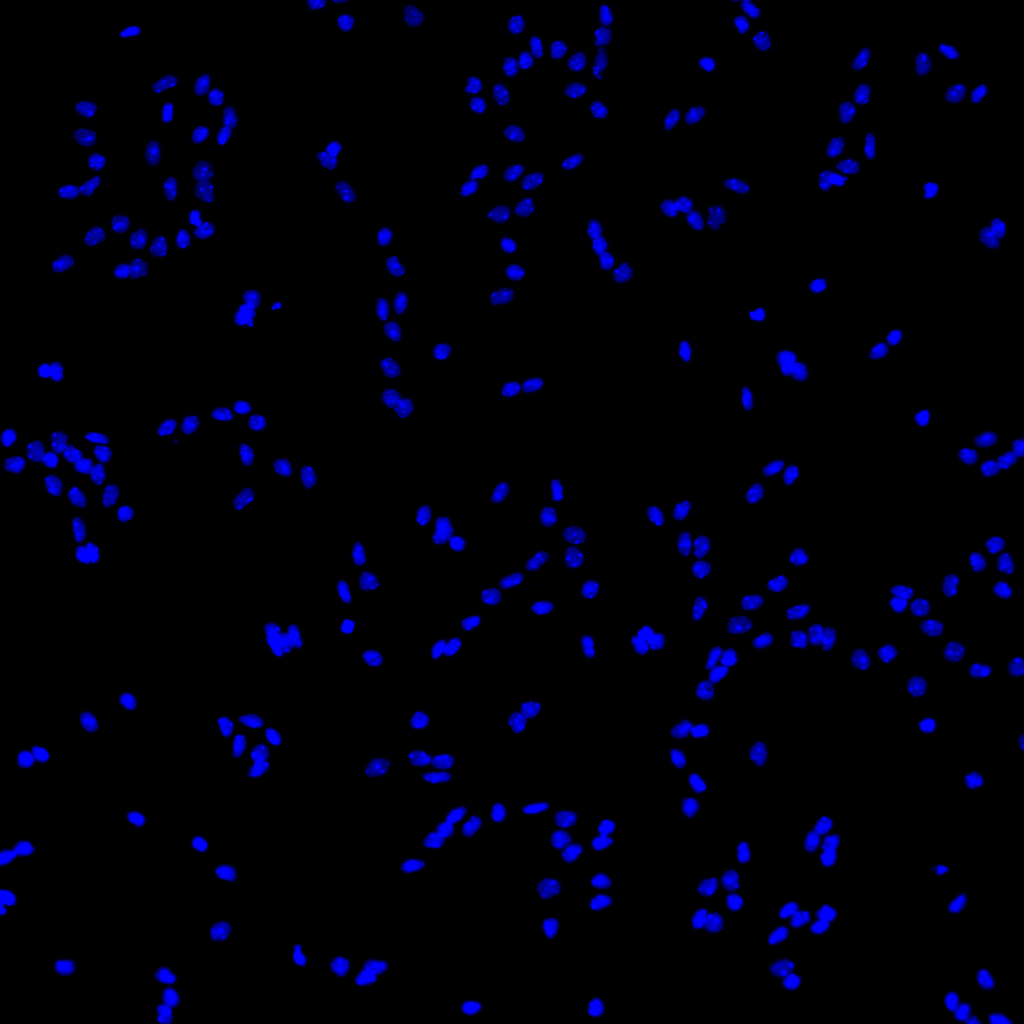

Supplement: S5 File — (ZIP) [file pone.0164217.s005.zip › Some other supplemental files (SOSF)/ICC.TIF/T-ET/t-et-200c1.tif]

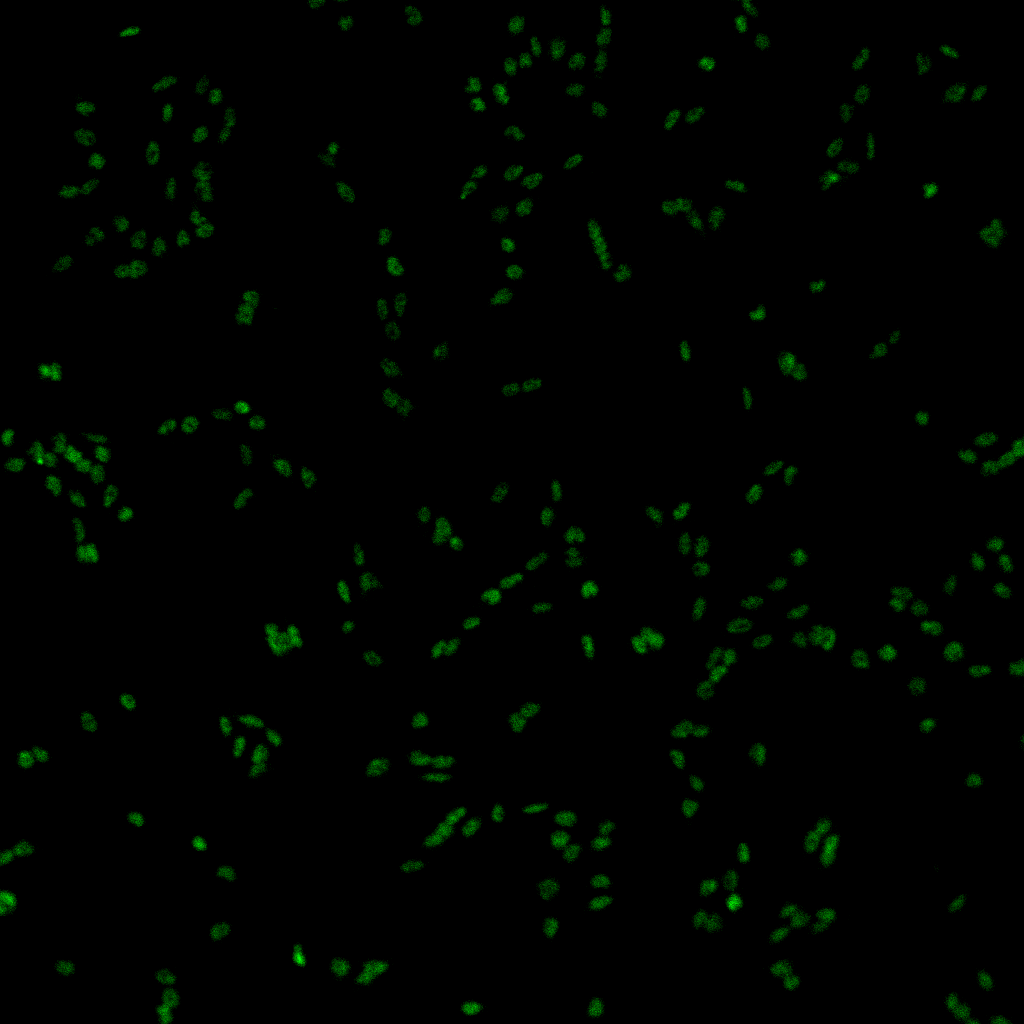

Supplement: S5 File — (ZIP) [file pone.0164217.s005.zip › Some other supplemental files (SOSF)/ICC.TIF/T-ET/t-et-200c2.tif]

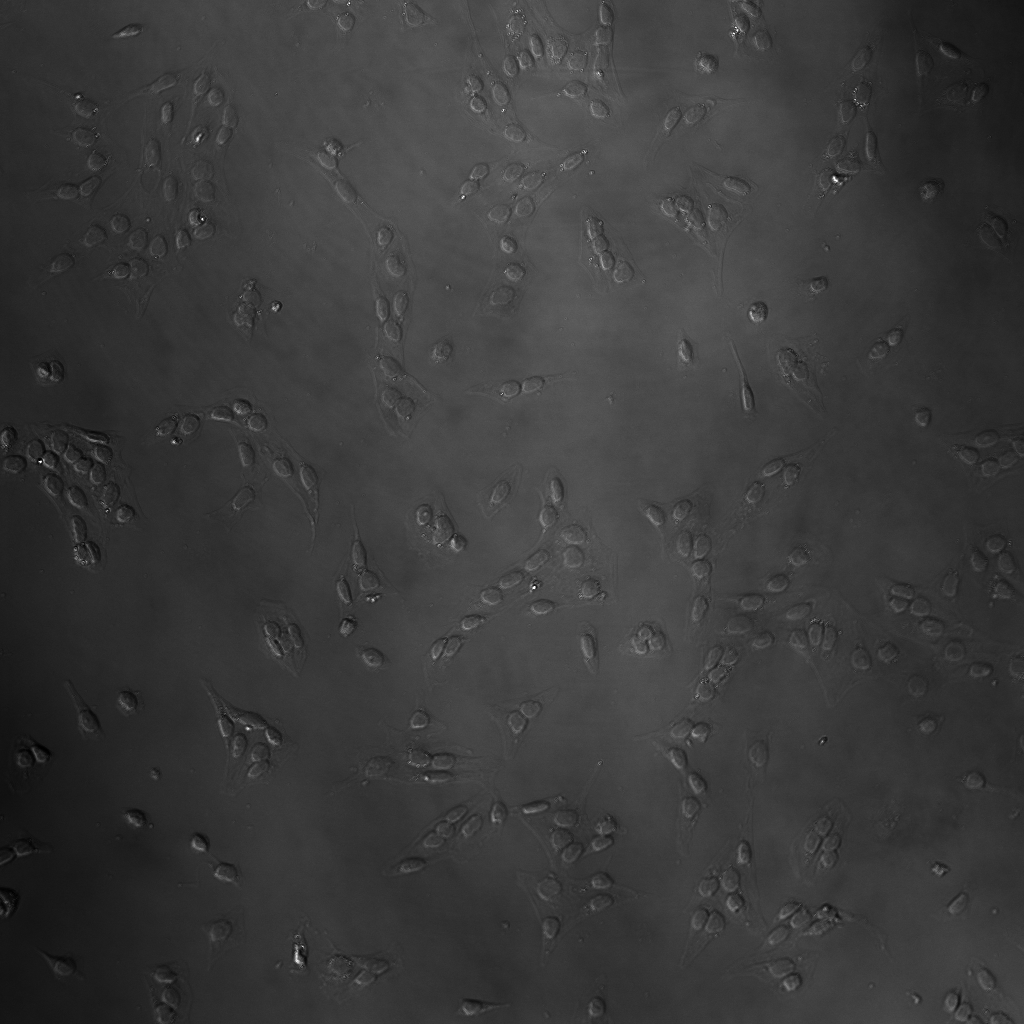

Supplement: S5 File — (ZIP) [file pone.0164217.s005.zip › Some other supplemental files (SOSF)/ICC.TIF/T-ET/t-et-200c3.tif]

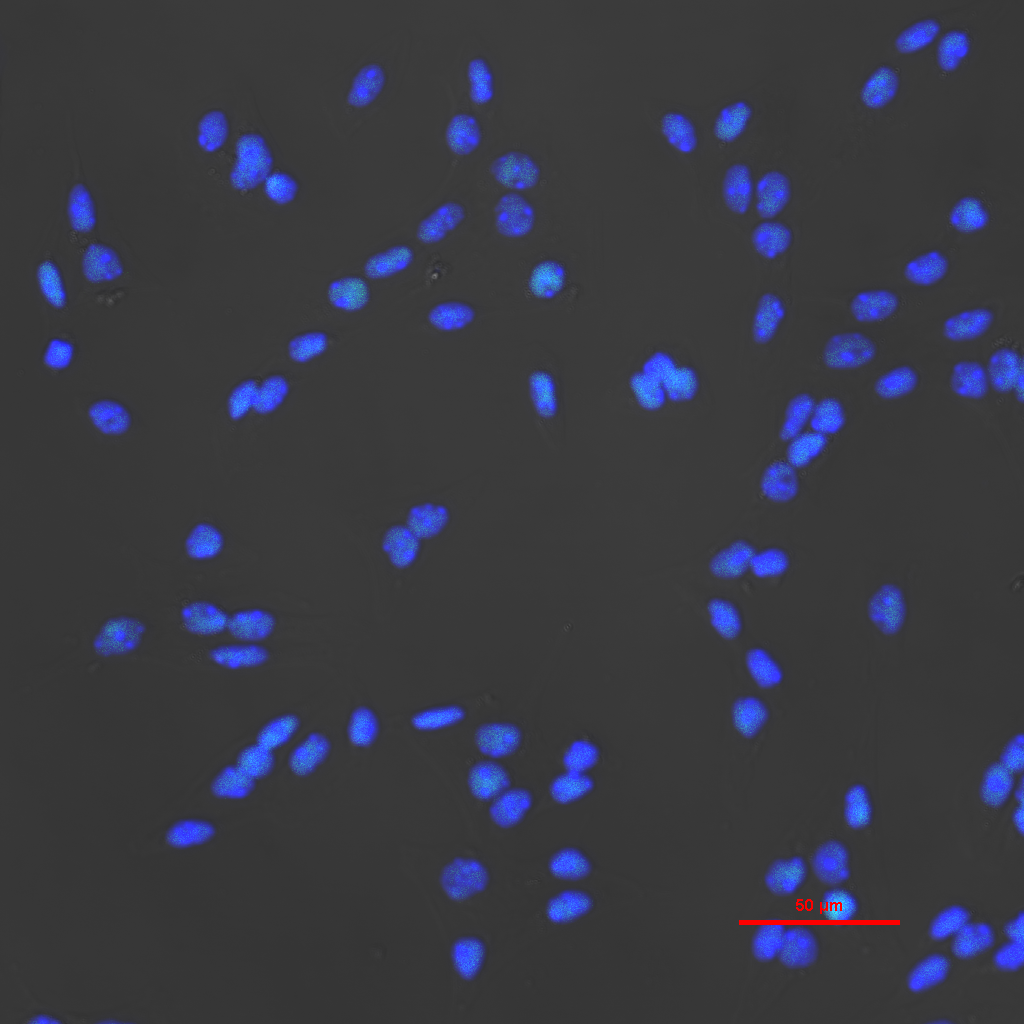

Supplement: S5 File — (ZIP) [file pone.0164217.s005.zip › Some other supplemental files (SOSF)/ICC.TIF/T-ET/t-et-400.tif]

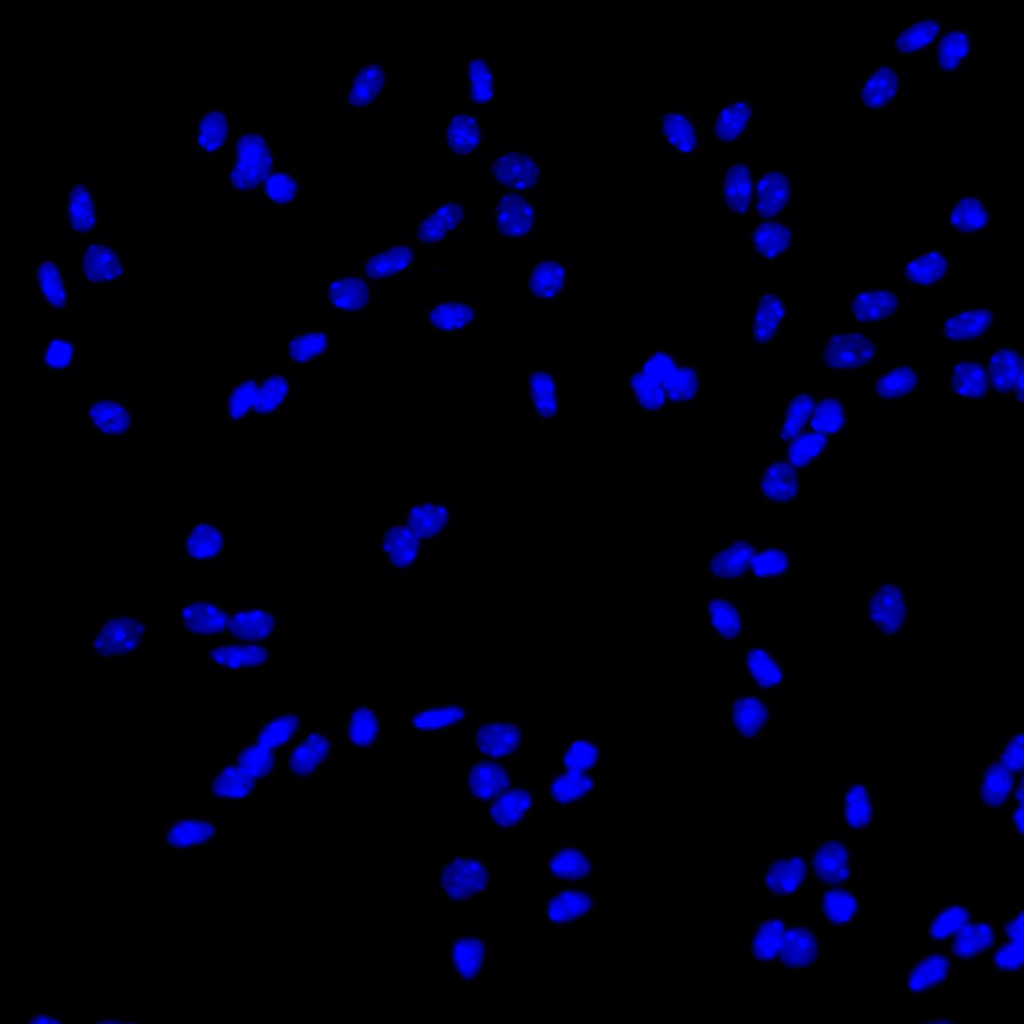

Supplement: S5 File — (ZIP) [file pone.0164217.s005.zip › Some other supplemental files (SOSF)/ICC.TIF/T-ET/t-et-400c1.tif]

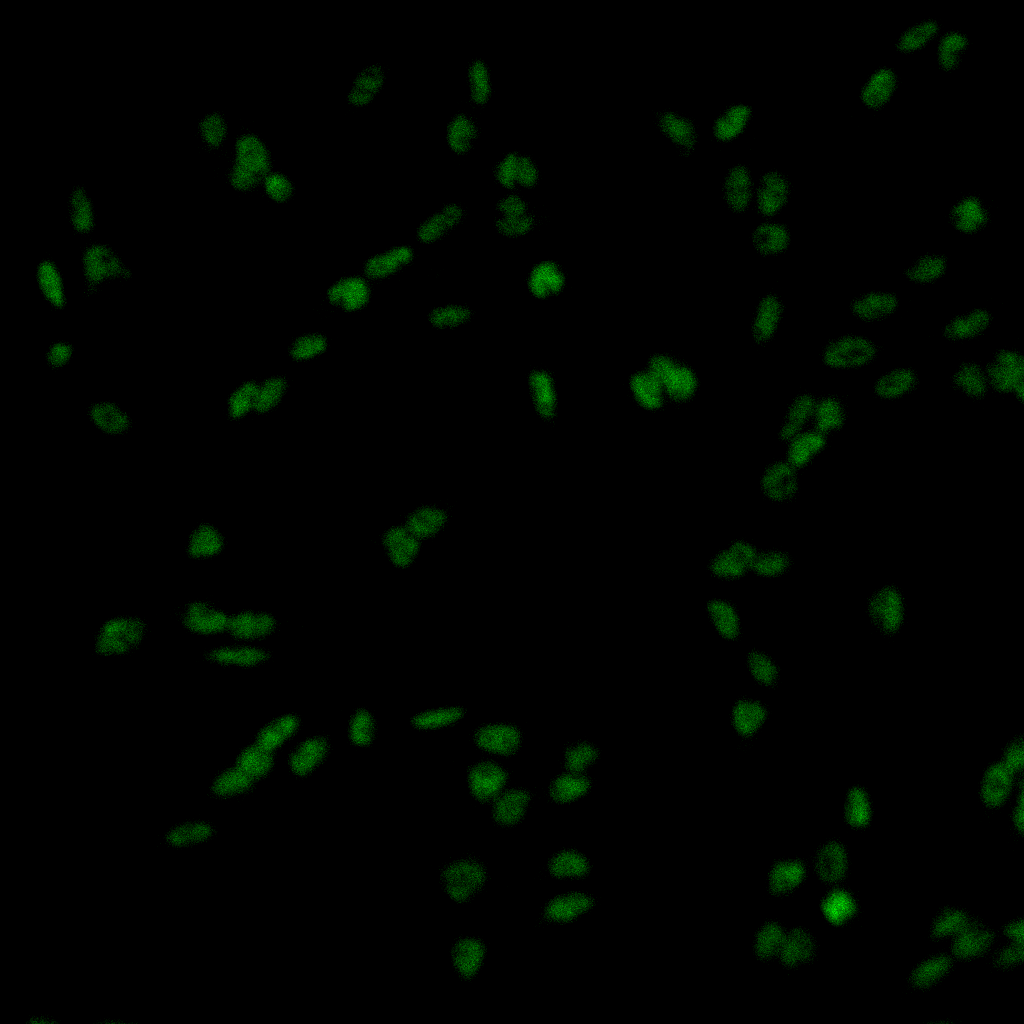

Supplement: S5 File — (ZIP) [file pone.0164217.s005.zip › Some other supplemental files (SOSF)/ICC.TIF/T-ET/t-et-400c2.tif]

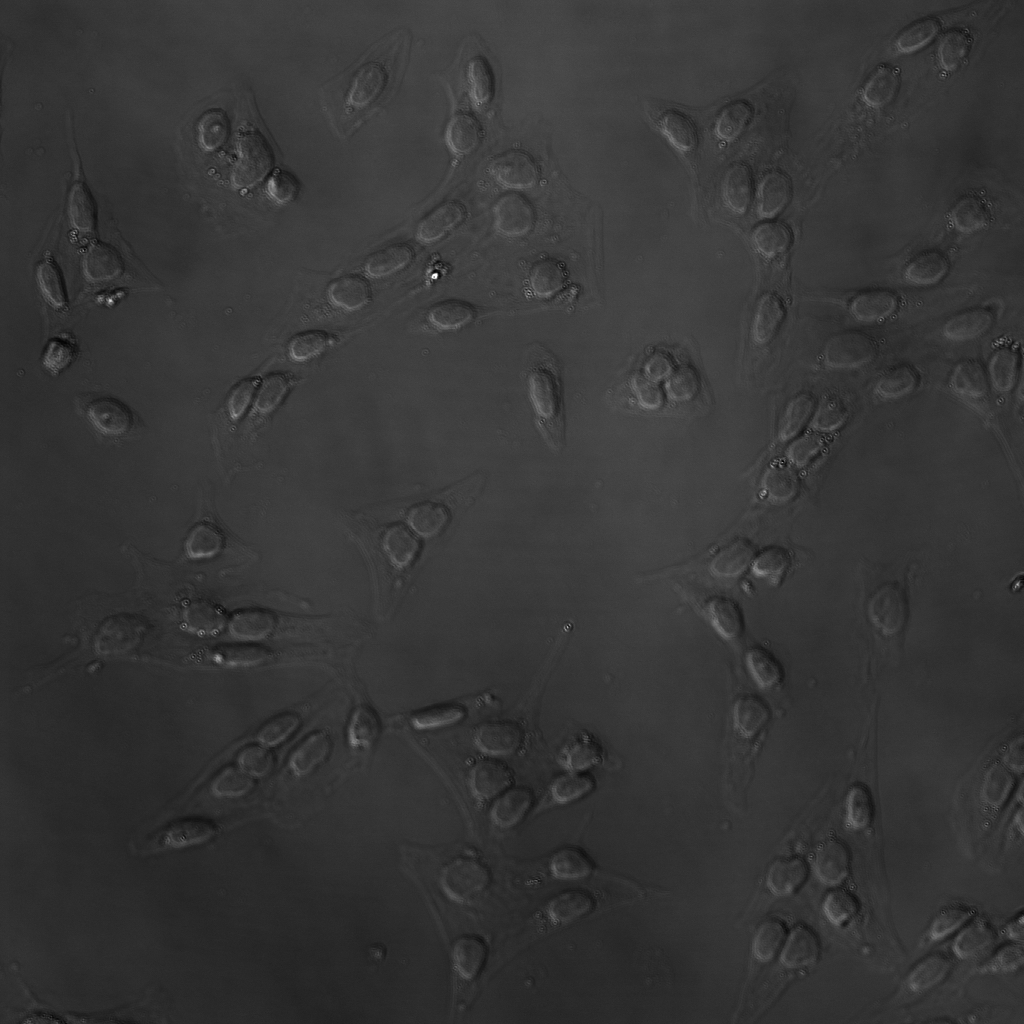

Supplement: S5 File — (ZIP) [file pone.0164217.s005.zip › Some other supplemental files (SOSF)/ICC.TIF/T-ET/t-et-400c3.tif]

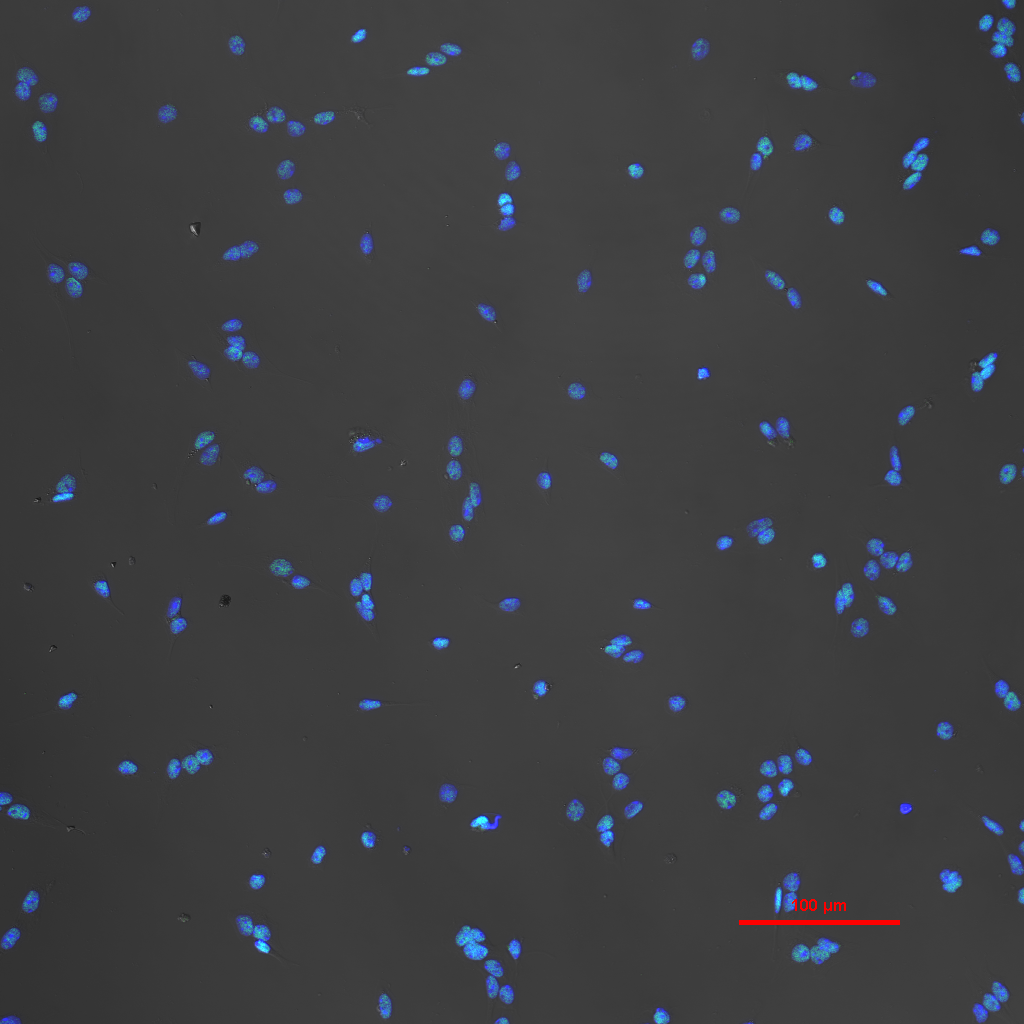

Supplement: S5 File — (ZIP) [file pone.0164217.s005.zip › Some other supplemental files (SOSF)/ICC.TIF/T-NET/t-net-200 (2).tif]

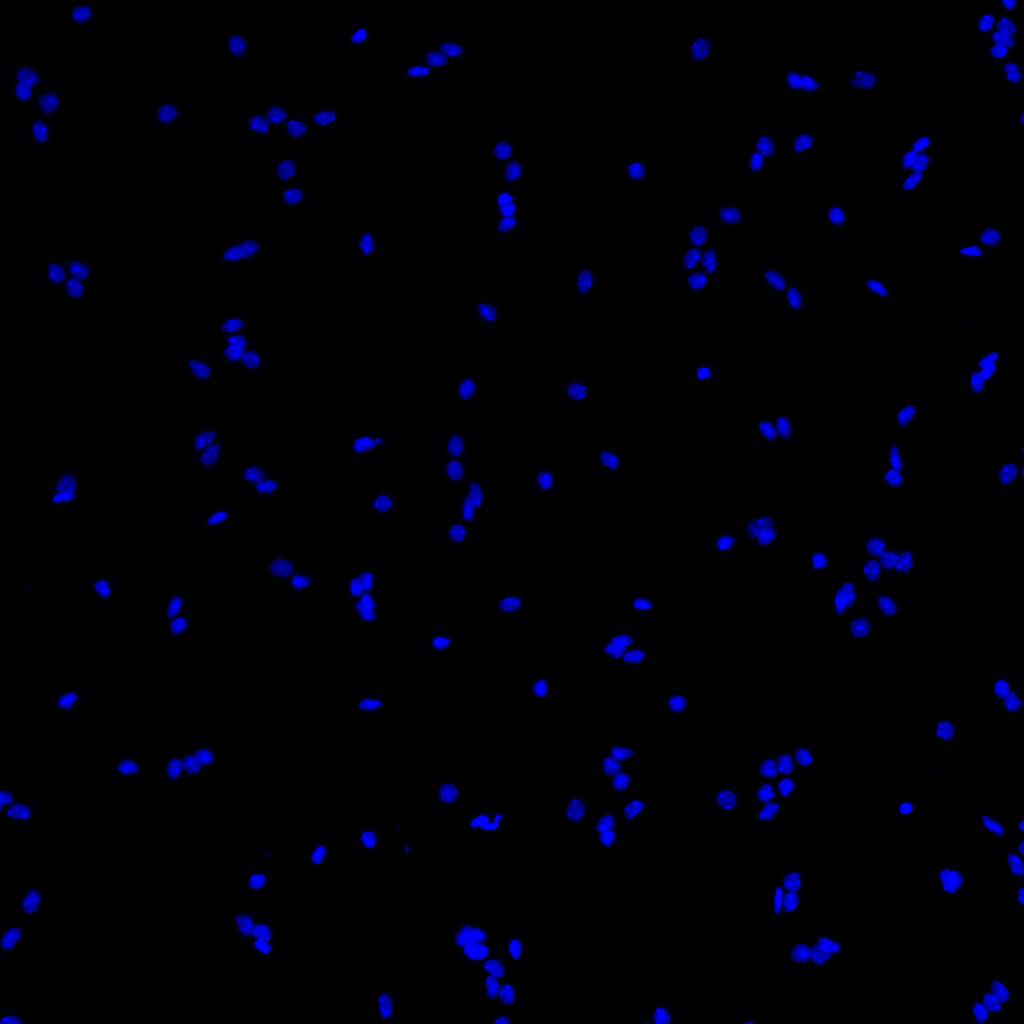

Supplement: S5 File — (ZIP) [file pone.0164217.s005.zip › Some other supplemental files (SOSF)/ICC.TIF/T-NET/t-net-200 (2)c1.tif]

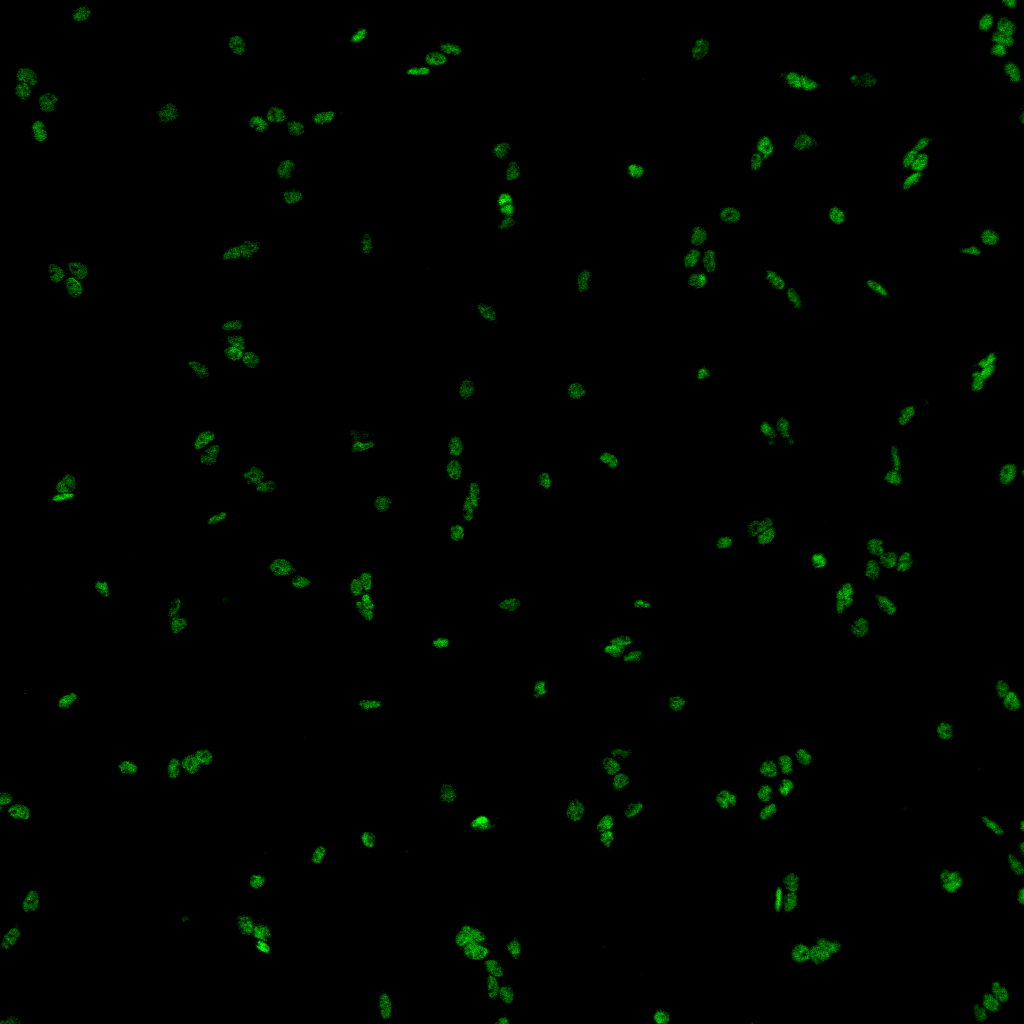

Supplement: S5 File — (ZIP) [file pone.0164217.s005.zip › Some other supplemental files (SOSF)/ICC.TIF/T-NET/t-net-200 (2)c2.tif]

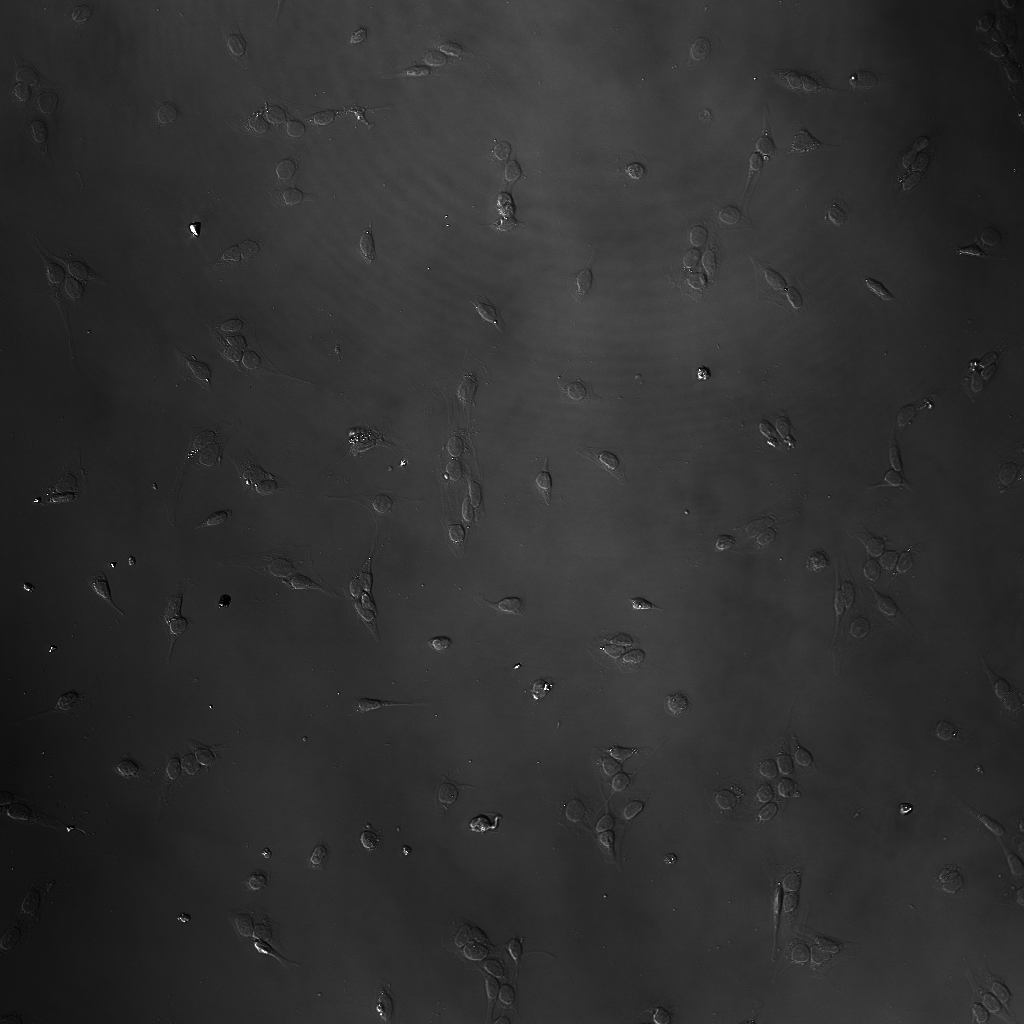

Supplement: S5 File — (ZIP) [file pone.0164217.s005.zip › Some other supplemental files (SOSF)/ICC.TIF/T-NET/t-net-200 (2)c3.tif]

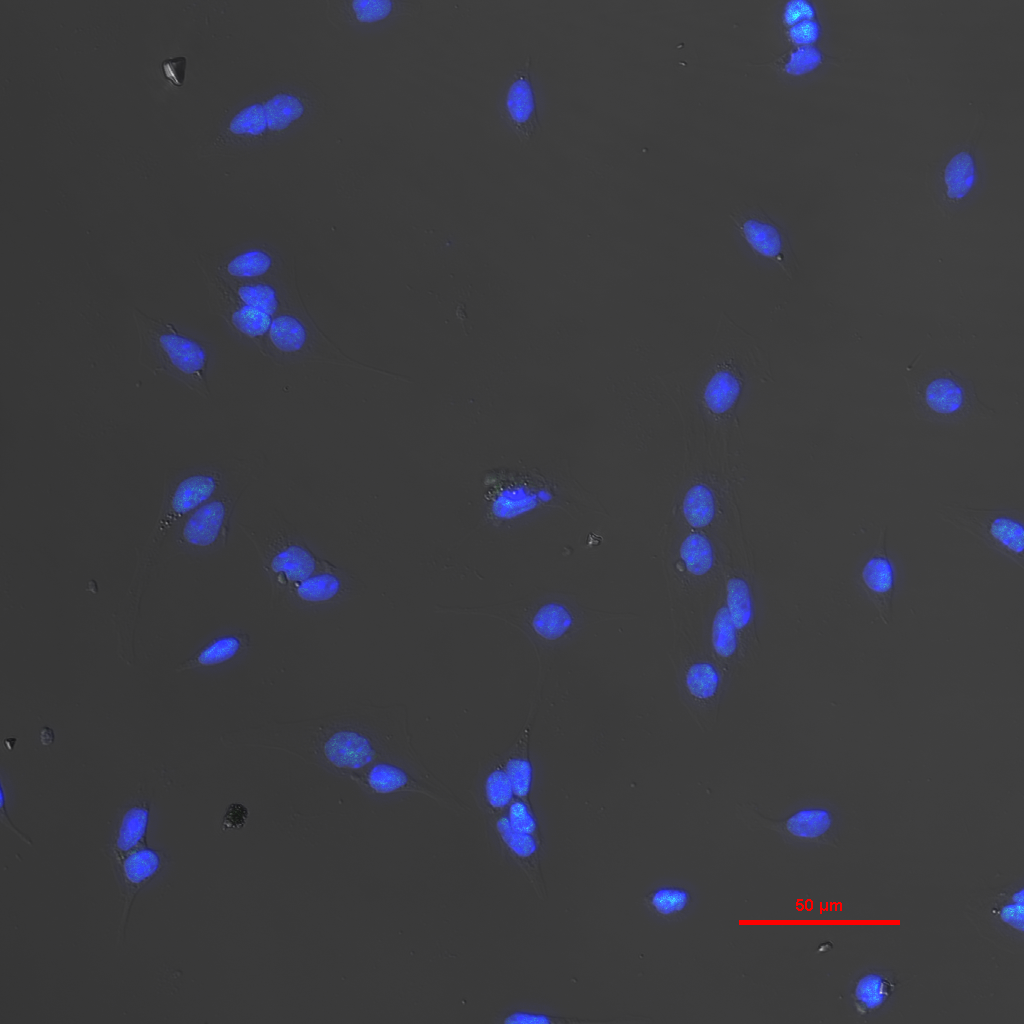

Supplement: S5 File — (ZIP) [file pone.0164217.s005.zip › Some other supplemental files (SOSF)/ICC.TIF/T-NET/t-net-400.tif]

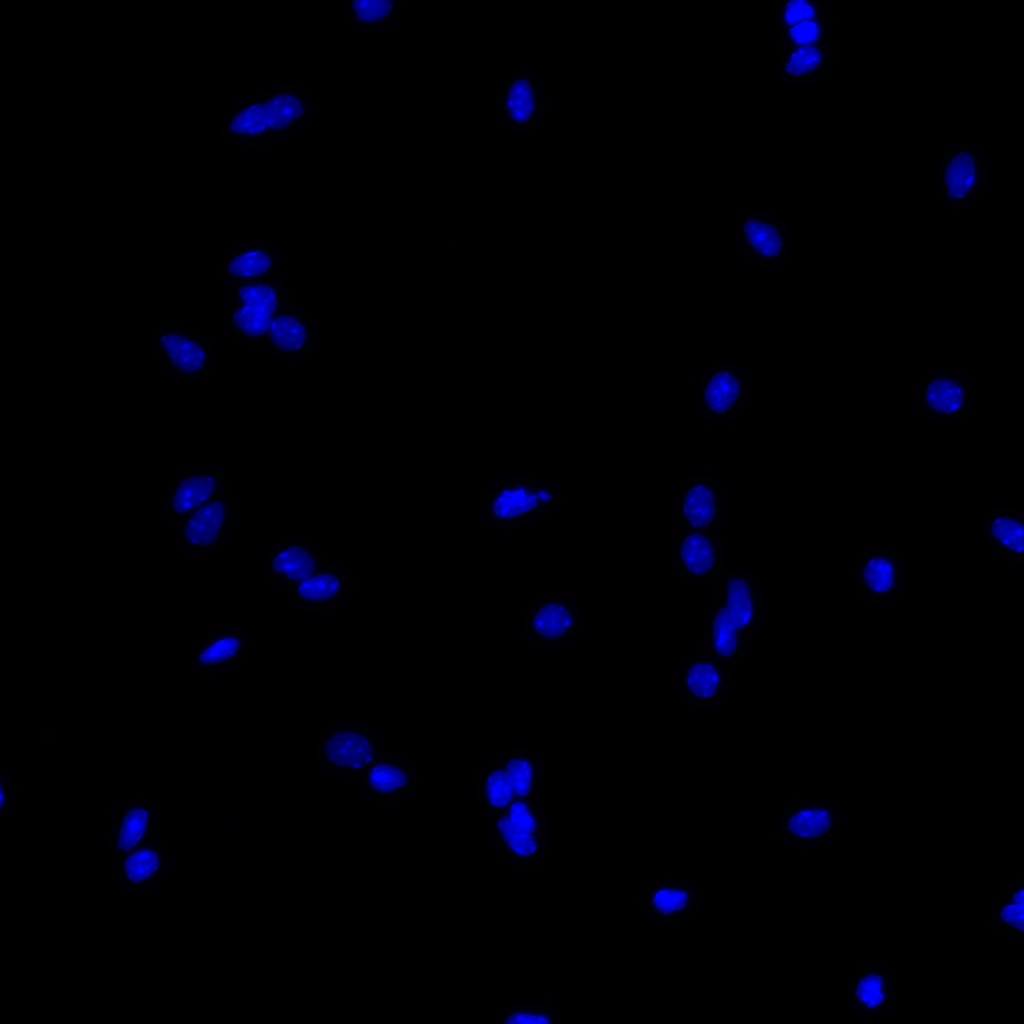

Supplement: S5 File — (ZIP) [file pone.0164217.s005.zip › Some other supplemental files (SOSF)/ICC.TIF/T-NET/t-net-400c1.tif]

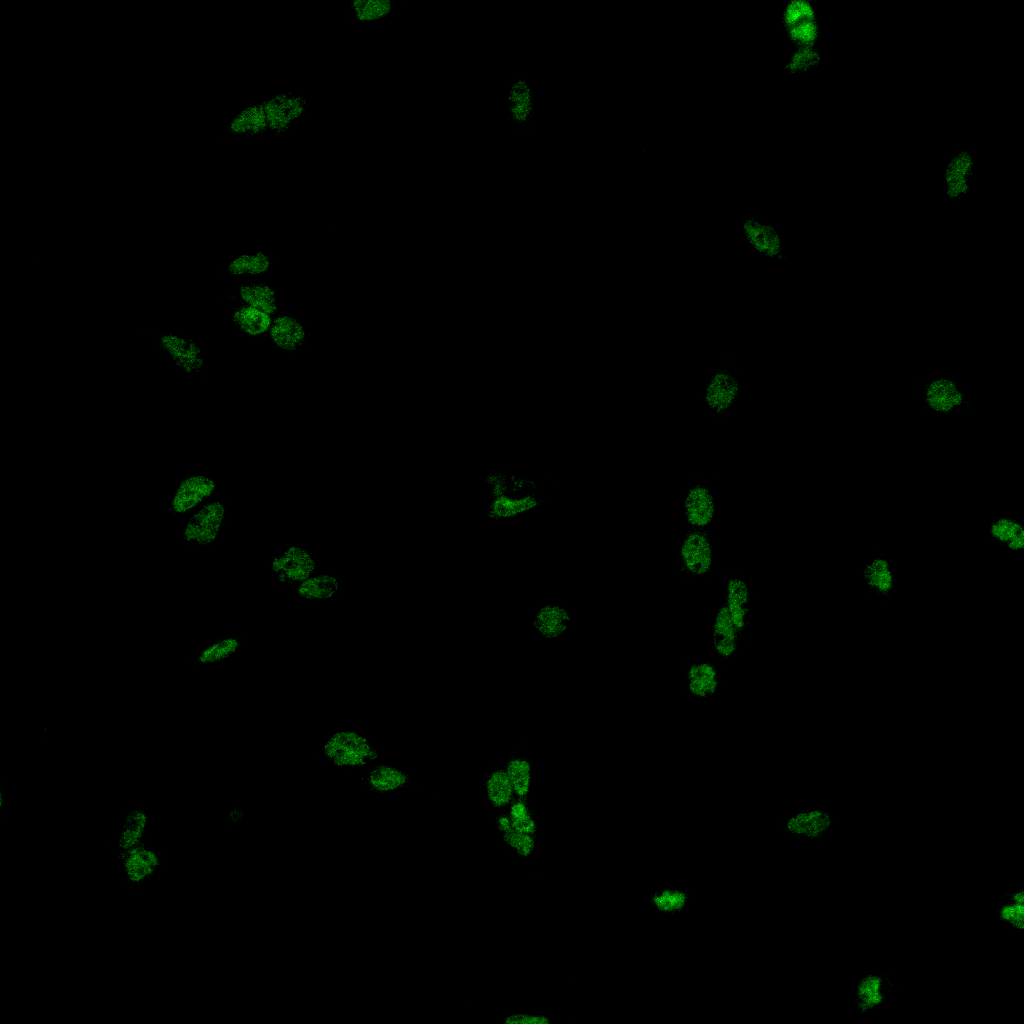

Supplement: S5 File — (ZIP) [file pone.0164217.s005.zip › Some other supplemental files (SOSF)/ICC.TIF/T-NET/t-net-400c2.tif]

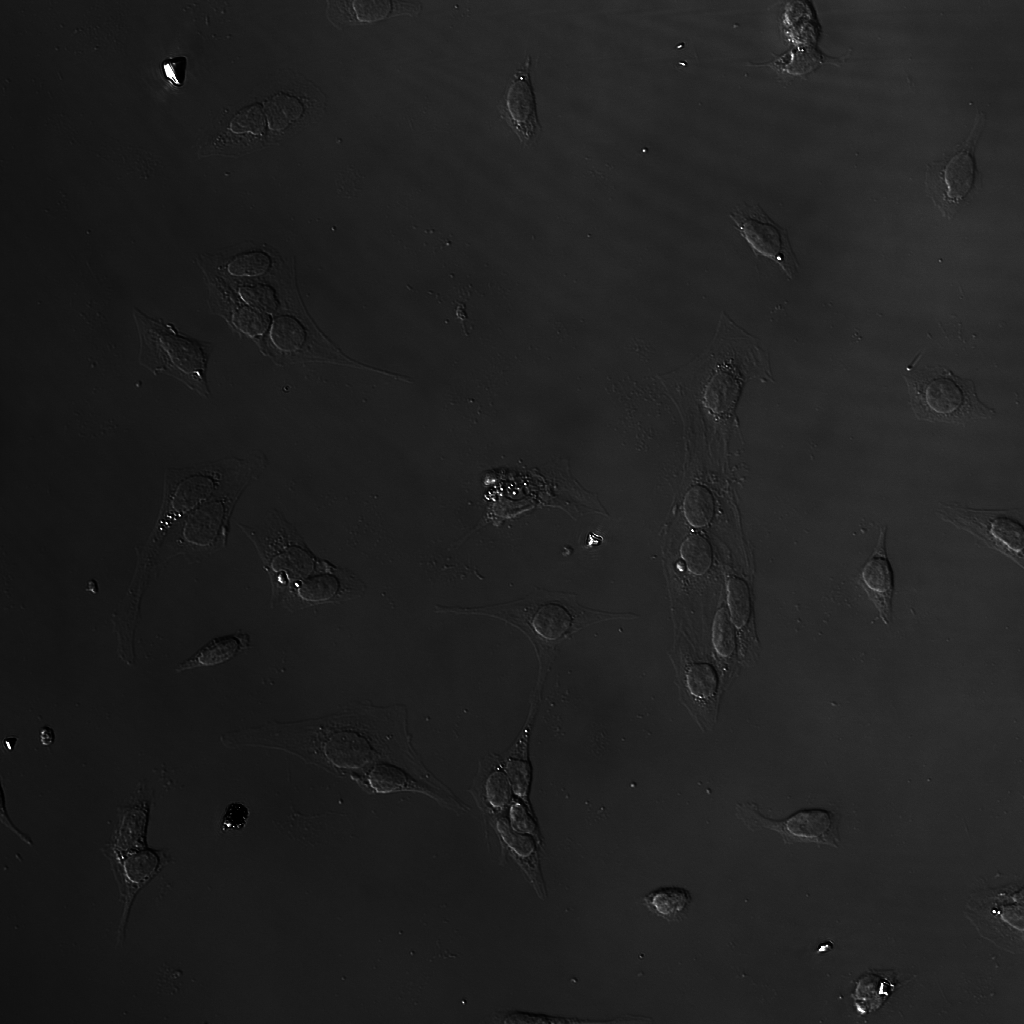

Supplement: S5 File — (ZIP) [file pone.0164217.s005.zip › Some other supplemental files (SOSF)/ICC.TIF/T-NET/t-net-400c3.tif]

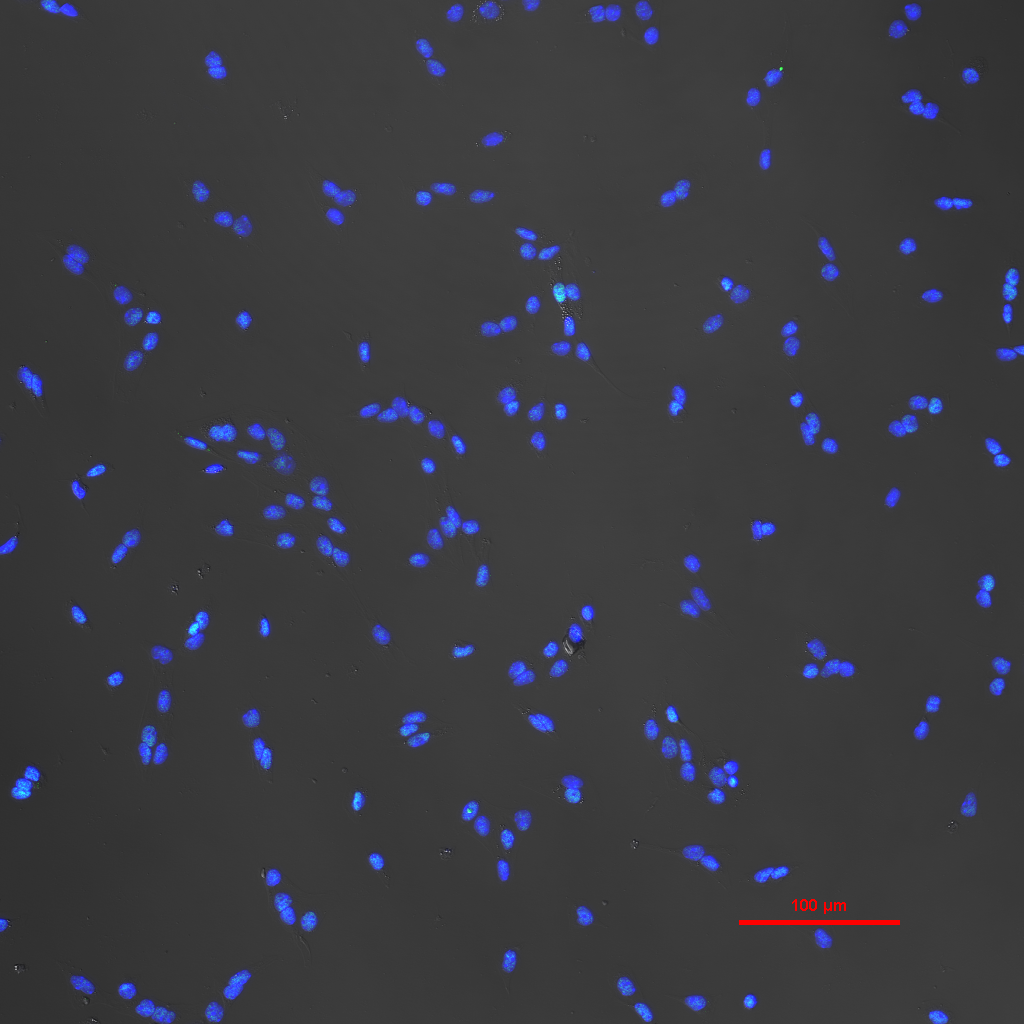

Supplement: S5 File — (ZIP) [file pone.0164217.s005.zip › Some other supplemental files (SOSF)/ICC.TIF/normal/normal-200.tif]

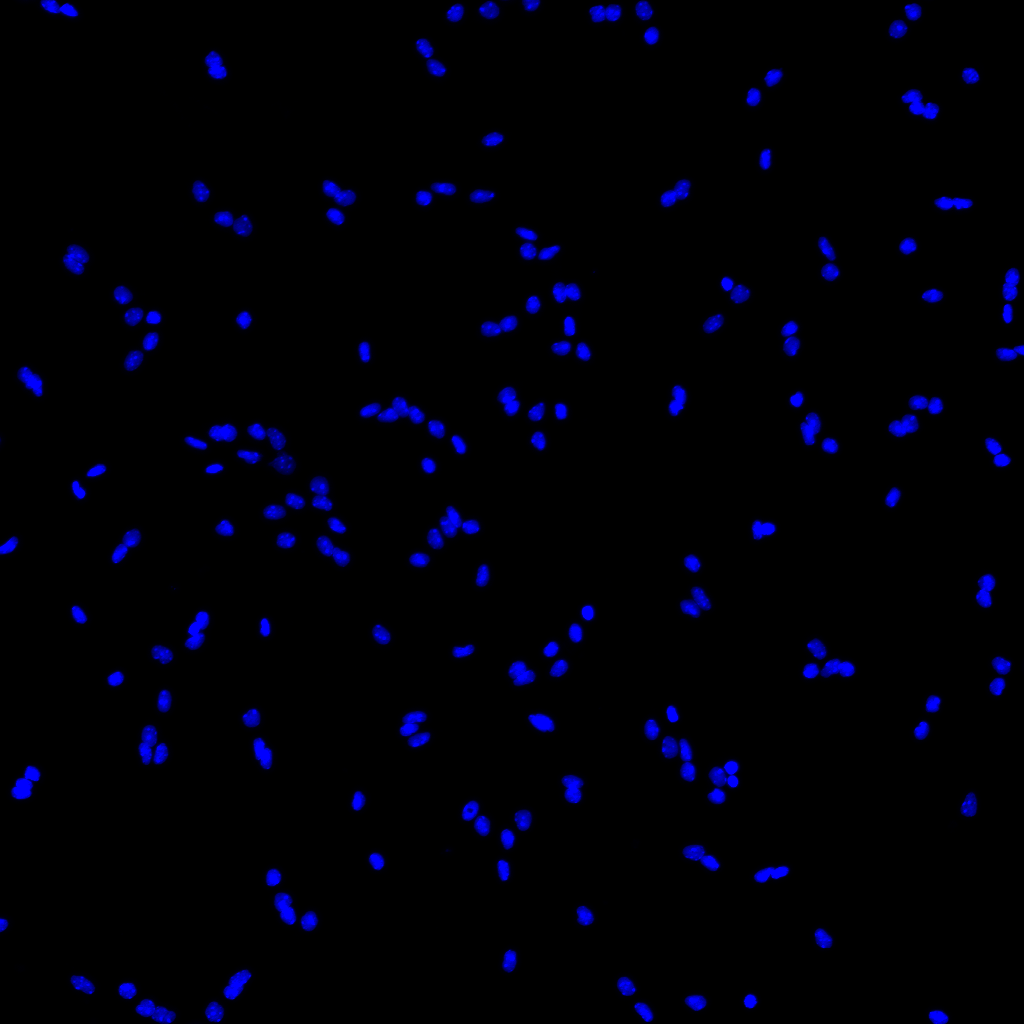

Supplement: S5 File — (ZIP) [file pone.0164217.s005.zip › Some other supplemental files (SOSF)/ICC.TIF/normal/normal-200c1.tif]

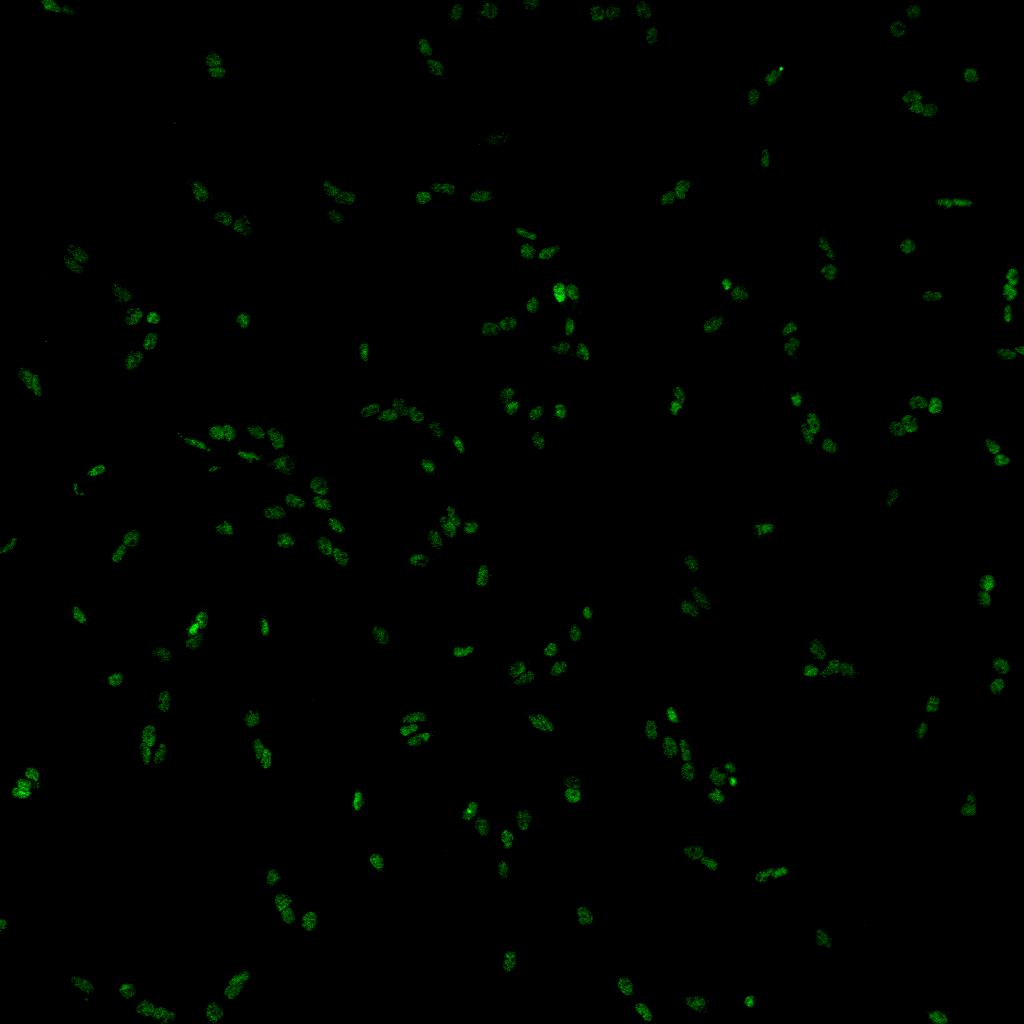

Supplement: S5 File — (ZIP) [file pone.0164217.s005.zip › Some other supplemental files (SOSF)/ICC.TIF/normal/normal-200c2.tif]

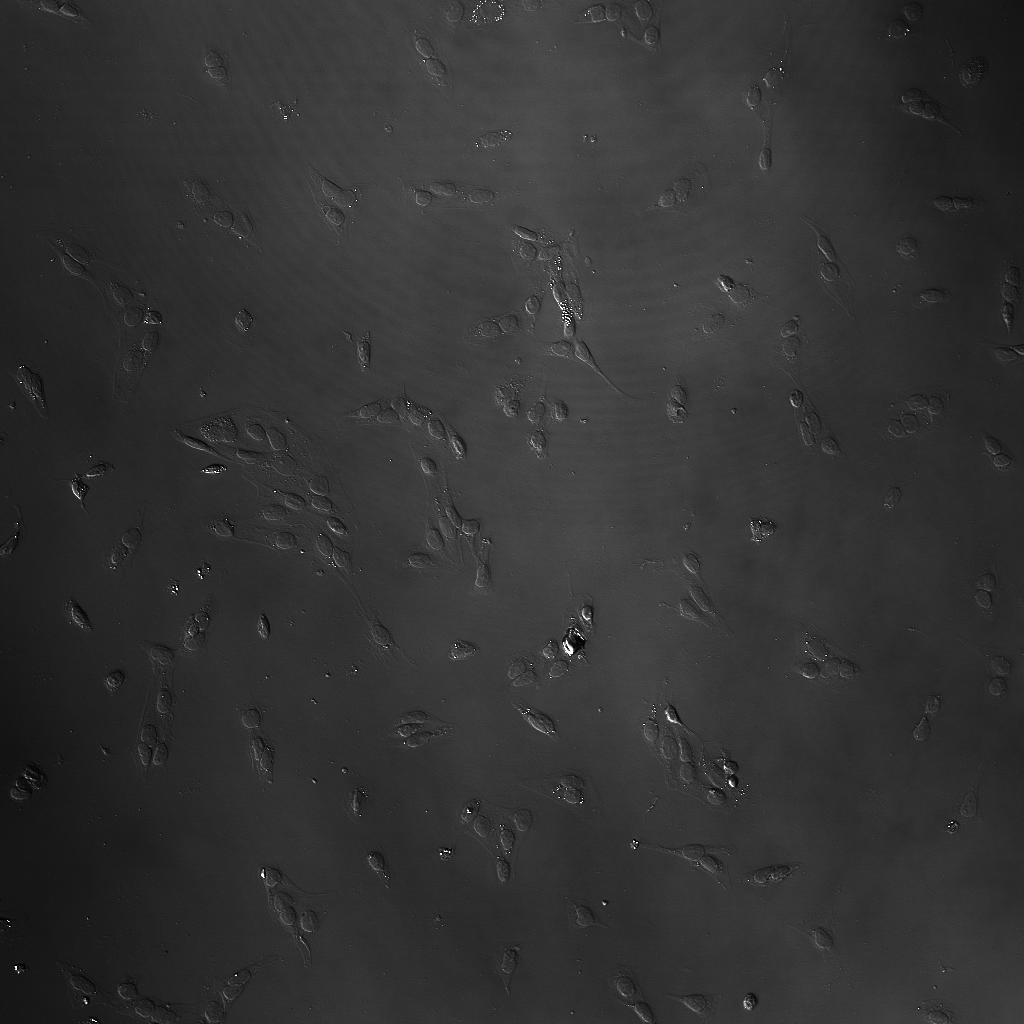

Supplement: S5 File — (ZIP) [file pone.0164217.s005.zip › Some other supplemental files (SOSF)/ICC.TIF/normal/normal-200c3.tif]

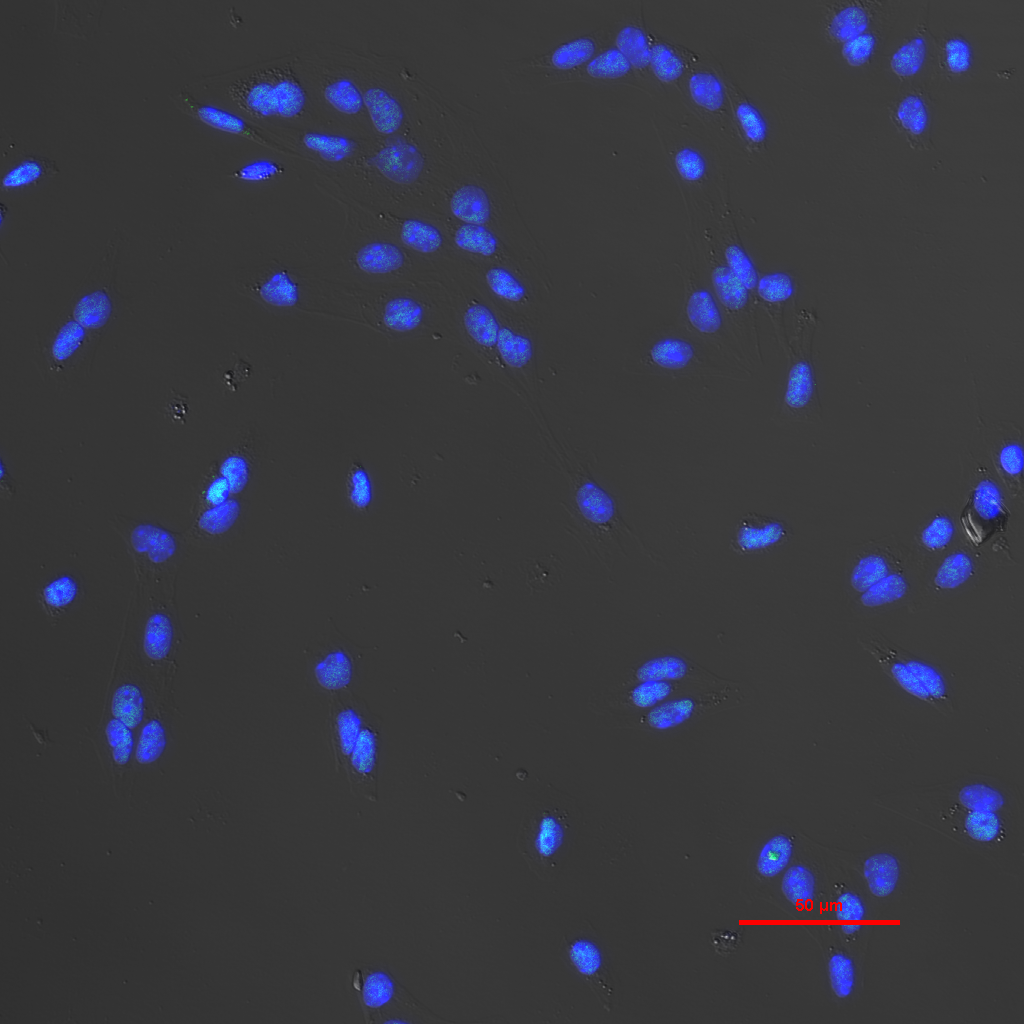

Supplement: S5 File — (ZIP) [file pone.0164217.s005.zip › Some other supplemental files (SOSF)/ICC.TIF/normal/normal-400.tif]

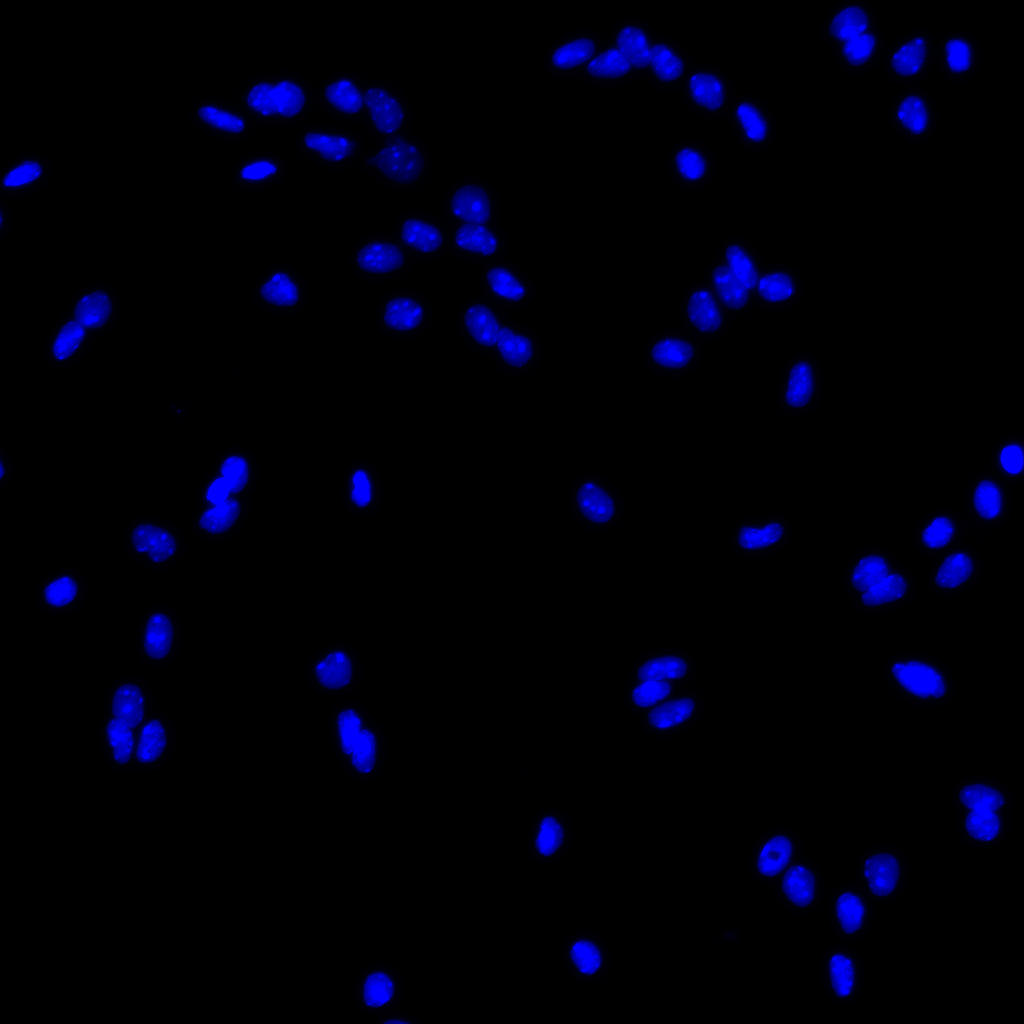

Supplement: S5 File — (ZIP) [file pone.0164217.s005.zip › Some other supplemental files (SOSF)/ICC.TIF/normal/normal-400c1.tif]

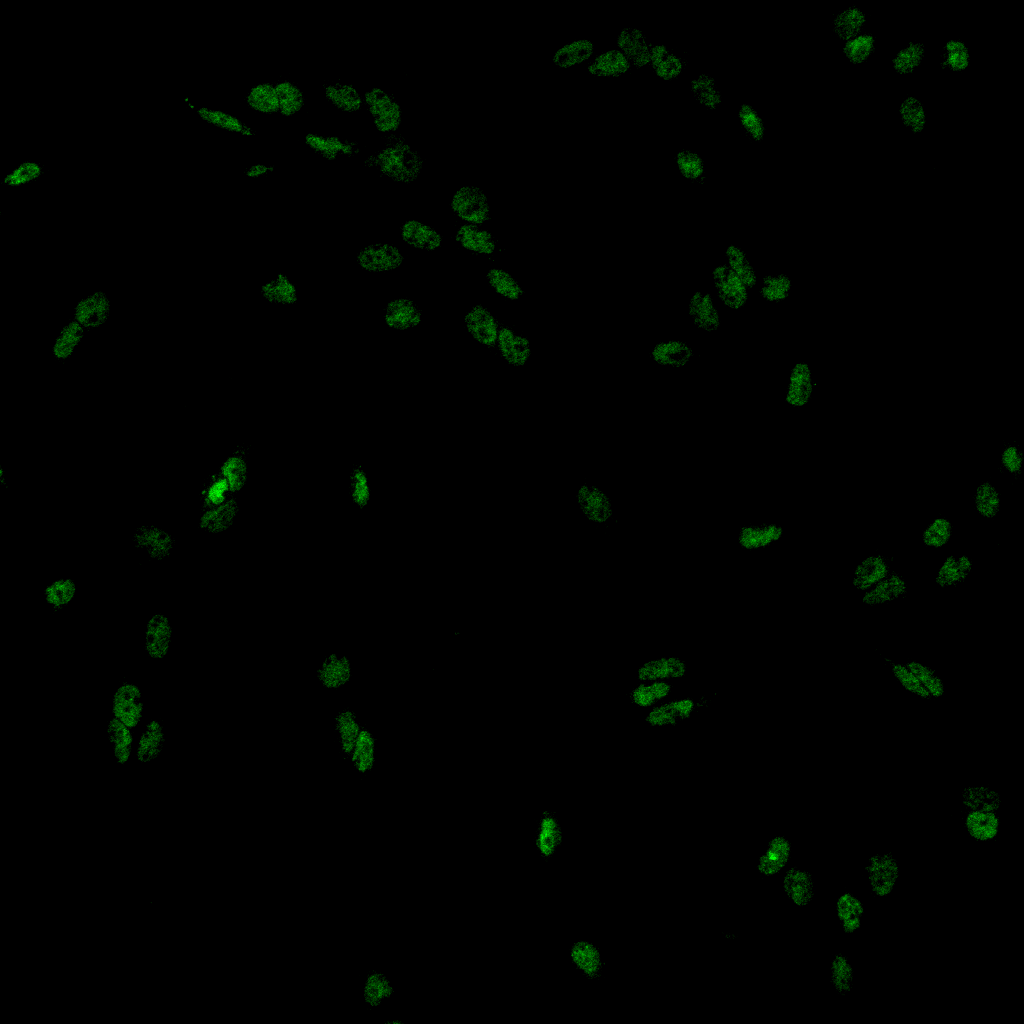

Supplement: S5 File — (ZIP) [file pone.0164217.s005.zip › Some other supplemental files (SOSF)/ICC.TIF/normal/normal-400c2.tif]

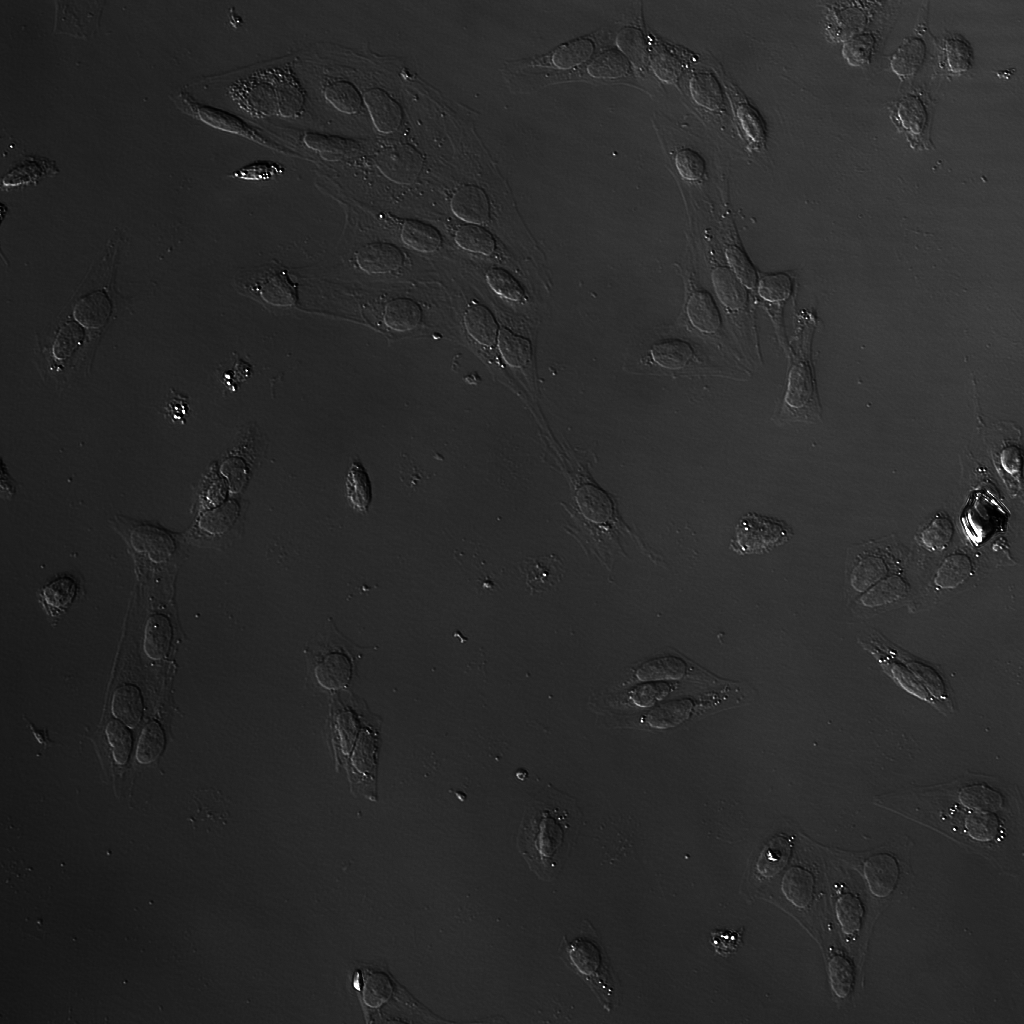

Supplement: S5 File — (ZIP) [file pone.0164217.s005.zip › Some other supplemental files (SOSF)/ICC.TIF/normal/normal-400c3.tif]
